# Identification and Expression Analysis of the Barley (*Hordeum vulgare* L.) Aquaporin Gene Family

**DOI:** 10.1371/journal.pone.0128025

**Published:** 2015-06-09

**Authors:** Runyararo M. Hove, Mark Ziemann, Mrinal Bhave

**Affiliations:** 1 Faculty of Science, Engineering and Technology, Swinburne University of Technology, PO Box 218, Hawthorn, VIC 3122, Australia; 2 Baker IDI Heart and Diabetes Institute, Melbourne, VIC 3004, Australia; Estación Experimental del Zaidín (CSIC), SPAIN

## Abstract

Aquaporins (AQPs) are major intrinsic proteins (MIPs) that mediate bidirectional flux of water and other substrates across cell membranes, and play critical roles in plant-water relations, dehydration stress responses and crop productivity. However, limited data are available as yet on the contributions of these proteins to the physiology of the major crop barley (*Hordeum vulgare*). The present work reports the identification and expression analysis of the barley MIP family. A comprehensive search of publicly available leaf mRNA-seq data, draft barley genome data, GenBank transcripts and sixteen new annotations together revealed that the barley MIP family is comprised of at least forty AQPs. Alternative splicing events were likely in two plasma membrane intrinsic protein (PIP) AQPs. Analyses of the AQP signature sequences and specificity determining positions indicated a potential of several putative AQP isoforms to transport non-aqua substrates including physiological important substrates, and respond to abiotic stresses. Analysis of our publicly available leaf mRNA-seq data identified notable differential expression of HvPIP1;2 and HvTIP4;1 under salt stress. Analyses of other gene expression resources also confirmed isoform-specific responses in different tissues and/or in response to salinity, as well as some potentially inter-cultivar differences. The work reports systematic and comprehensive analysis of most, if not all, barley AQP genes, their sequences, expression patterns in different tissues, potential transport and stress response functions, and a strong framework for selection and/or development of stress tolerant barley varieties. In addition, the barley data would be highly valuable for genetic studies of the evolutionarily closely related wheat (*Triticum aestivum* L.).

## Introduction

Aquaporins (AQPs) belong to the superfamily of membrane channels called the major intrinsic proteins (MIPs). The plant MIPs (often generically called ‘aquaporins’) are typically divided into seven subfamilies: the plasma membrane intrinsic proteins (PIPs), tonoplast intrinsic proteins (TIPs), nodulin-26-like intrinsic proteins (NIPs), small, basic intrinsic proteins (SIPs) [1] and the novel, less common subfamilies of GlpF-like intrinsic protein (GIP1;1) [2], hybrid intrinsic proteins (HIPs) and the uncategorized X intrinsic proteins (XIPs) [3]. Based on the ar/R (aromatic/Arginine) selectivity filter residues, NIPs are typically divided into group I (WVAR; e.g., AtNIP1;1, OsNIP1;1), group II [A(V/I/A)(G/A)R; e.g., AtNIP5;1, OsNIP3;1;] and group III (GSGR; e.g., OsNIP2;1) [4]. Four main subfamilies (PIPs, TIPs, NIPs, SIPs) have been identified in both primitive and higher plants, while HIPs and GIPs have only been identified in mosses [3] and XIPs identified in mosses and several dicotyledonous plants, e.g., tobacco, potato and tomato [5] and soybean [6], but not monocots. The plant MIP families show a high gene multiplicity, with 36 isoforms reported in maize [7], 38 in rice [8], and 66 in soybean [6]. All MIPs exhibit a number of characteristic features (reviewed in [1]), including (i) six trans-membrane helices (TM1-TM6) and five inter-helical loops (LA-LE); (ii) two short helices (HB, HE) that contain the highly conserved Asn-Pro-Ala (NPA) motifs that form the pore that allows a single-file passage of water molecules; (iii) the ar/R selectivity filter, comprised of four residues (one from TM2, one from TM5, two from LE), and shown to significantly influence the nature of transport substrates; (iv) five positions (P1-P5) likely important for discriminating between AQPs and glycerol transporters and (v) a conserved motif Ala-Glu-Phe (AEF or AEFXXT) in TM1, of unclear function.

Plant MIPs have been shown to have significant roles in water homeostasis and response to salinity and drought, and additionally, many isoforms also transport other substrates such as ammonia, silicon, CO_2_ and boron (reviewed in [1, 8]). Reduced expression of certain PIP aquaporins and reduced water uptake under nitrogen supply provided as ammonium rather than nitrate [9] further suggests a mechanism of direct control to reduce ammonium toxicity (sole ammonium being toxic to many plants compared to a mixed-feed with nitrate), although how the nutrient/antinutrient is distinguished and the stimulus is linked to modulations of aquaporin expression and water transport remains unclear. The diverse aquaporins thus play important roles in many life processes of plants such as photosynthesis, nitrogen fixation, nutrient acquisition, reduced uptake and/or detoxification of toxic compounds, and other environmental stress responses, and hold immense genetic potential for crop improvement via varietal selection or transgenic strategies. Drought, salinity and nutrient stresses impede the productivity of barley and/or wheat significantly. However, despite the nutritional and economic importance of these crops, the roles of AQPs as genetic factors that may affect the growth and yield of these crops remain rather poorly understood. In case of wheat, the presence of at least 35 AQPs (PIPs and TIPs only) has been reported [10], with some likely discrepancies in distinction of some genes versus homeologues due to its polyploidy, and its NIP and SIPs are little studied. Similarly, studies on barley have been limited to some individual genes, especially PIPs [11– 15]. This study focusses on analysing the MIP superfamily in barley.

Barley is an important food crop which belongs to grass family Poaceae, subfamily Pooideae and tribe Triticeae, which also includes the larger cereal crop, wheat. However, barley is more adaptable and resilient than wheat [16]. It is more tolerant to drought, salinity and cold, and can be cultivated at higher altitudes and latitudes and farther into deserts than other cereal crops [17]. Barley seems to be able to maintain the root hydraulic conductivity (important for maintenance of transpiration), at least under moderate salinity stress [13] as compared to wheat [18], possibly explaining its better tolerance. Aquaporins such as PIPs have crucial roles in water uptake, its transmembrane transport and osmotic mechanisms. Hence a deeper understanding of these genes in barley will be essential for crop improvement through selective breeding and transgenics, both in barley and wheat. Its close evolutionary relatedness to wheat and its smaller, diploid, now-sequenced genome make it an excellent candidate for this purpose. The findings of the present study have led to possibly the entire AQP family of barley, including the gene sequences, expression patterns and the potential transport substrates of the encoded proteins.

## Materials and Methods

### Identification of barley aquaporin sequences

Barley AQP sequences were identified from the leaf mRNA-seq dataset obtained earlier, the NCBI UniGene database, and the barley draft genome sequence, as follows. For mining the leaf mRNA-seq data (publicly available, deposited as Sequence Read Archive accession number SRA062960 ([19]; including supplemental data) to identify the transcripts representing AQPs, keyword searches were carried out for putative protein encoding sequences annotated as 'aquaporin', 'PIP', 'TIP', 'NIP', 'SIP', 'intrinsic', 'channel', 'transmembrane' and 'nodulin-like' based on their similarity to rice proteins. For accessing the NCBI UniGenes, the accession numbers of cDNAs corresponding to each barley UniGene were retrieved from their profile in NCBI (http://www.ncbi.nlm.nih.gov/unigene/; last accessed December 2014). The cDNAs were translated in Gene Runner (http://www.generunner.net/) and inspected for conserved features such as NPA motifs. The nearest barley relatives of rice AQP genes identified earlier [8] were mined from the NCBI UniGene database. All thus-identified UniGenes were also translated and the barley cDNAs representing these identified from NCBI as above. For searching the barley draft genome, the cDNAs identified in mRNA-seq and the rice AQPs (http://rice.plantbiology.msu.edu/; last accessed December 2014) were subjected to BLASTn against the International Barley Genome Sequencing Consortium (IBGSC) [20] IPK Barley Blast Server (http://webblast.ipk-gatersleben.de/barley/; last accessed December 2014) high confidence CDS (HC_genes_CDS_Seq) and full length cDNA databases (ftp://ftpmips.helmholtz-muenchen.de/plants/barley/public_data/genes/; last accessed December 2014) (e-value threshold <0.01). All data were used to compile a comprehensive list of all identified barley AQP cDNAs. These were then subjected to BLASTn against the IBSC assembly_WGSMorex and assembly_WGSBowman databases (http://webblast.ipk-gatersleben.de/barley/; last accessed December 2014) (e-value <0.01) to obtain their genomic sequences and physical locations. Additionally, the low confidence gene set (LC_genes_CDS_Seq) was also mined to note any other potential AQPs.

### Sequence translations, analyses, alignments, construction of phylogenetic trees

All cDNAs were translated in Gene Runner and the putative protein sequences analysed. The subcellular locations of putative proteins were predicted with WoLF PSORT (http://wolfpsort.org/), PSORT (http://psort.hgc.jp/form.html) and Predotar (http://urgi.versailles.inra.fr/predotar/predotar.html). The DNA and protein sequences were aligned in Bioedit v7.1.3 (http://www.mbio.ncsu.edu/BioEdit/bioedit.html) using CLUSTALW2 (http://www.ebi.ac.uk/Tools/clustalw2/index.html). Phylogenetic trees were constructed based on the nucleotide alignments shown in S1 Fig using MEGA5 (http://www.megasoftaware.net/) and maximum likelihood method, with bootstrapping set at 1000 replications. Alignments of the genomic sequences and cDNAs were conducted using the gene structure display server (GSDS; http://gsds.cbi.pku.edu.cn/) to determine the intron-exon structures.

### Analysis of expression of barley aquaporins

The mRNA-seq data were analysed for relative expression of AQPs in the control and salt-stressed leaf RNAs. Details of each barley EST corresponding to a UniGene (cultivar, tissue, treatment, development stage) were sourced from the NCBI EST database (http://www.ncbi.nlm.nih.gov/nucest/16322814) (data not shown). Also, the probeset IDs corresponding to AQPs were obtained from HarvEST:Barley v1.83 (http://harvest.ucr.edu/) using the ‘search by GenBank number, EST name or unigene number’ tool, and some also from Besse *et al*. [21]. The IDs were used for analysis of their expression profiles in Genevestigator (https://www.genevestigator.com/gv/). The RNA-seq data of the eight tissues related to the IBGSC high confidence gene predictions (ftp://ftpmips.helmholtz-muenchen.de/plants/barley/public_data/expression/README) were used to obtain expression scores for genes with MLOC numbers and generate a heat map of log2 normalised expression.

## Results

### Identification of barley aquaporin genes

The search for AQPs in the SUT barley leaf transcriptome developed earlier [19] [GenBank SRA062960] by keyword and rice locus identified thirty-one UniGenes. The corresponding cDNA numbers to these (e.g., http://www.ncbi.nlm.nih.gov/UniGene/clust.cgi?UGID=2919645&TAXID=4513&SEARCH=Hv.23281) showed the cDNAs represented twenty-two annotated (eleven PIPs, seven TIPs, four NIPs) and eight unannotated genes (Table 1; S1 Table). By mining the IBGSC barley genome project CDS and genomic databases with the cDNAs identified from the MSU rice cDNA database and the barley leaf mRNA-seq data, 36 CDSs and 40 genomic sequences could be identified altogether. By comparing these to the rice and barley cDNAs, 22 genomic sequences could be annotated as above, while sixteen were unannotated, comprised of eight as above and eight additional ones. Additionally, a genomic sequence identified in Bowman and Morex genomes (contig_222714; contig_401367) was not present in the CDS database, and a BLASTn against NCBI revealed that both contigs corresponded to HvTIP5;1 cDNA (AB540227). Based on % identities to other reported barley sequences (Table 1) or to orthologs in rice, maize or sorghum (data not shown), it appears that some annotations in Genbank may need to be revised, i.e., (i) AB540229, currently annotated as HvNIP2;1, seems better annotated as HvNIP2;2; (ii) AB710142 annotated as HvNIP2;2 should be HvNIP2;1; (iii) GU584119 annotated as HvTIP1;3 should be HvTIP1;2; (iv) GU584121 annotated as HvTIP2;1 should be HvTIP2;3 (Table 1). The BLASTn of the unannotated sequences against the Rice MSU cDNA database revealed they represented seven PIP2s (tentatively called *HvPIP2a-g*), three TIPs (*HvTIP3*, *HvTIP4a*, *HvTIP4b*), four NIPs (*HvNIP2*, *HvNIP3a*, *HvNIP3b*, *HvNIP4*) and two SIPs (*HvSIP1*, *HvSIP2*). BLAST queries of PpGIP1;1 [2], PpHIP1;1 [3] and GmXIP1;1 [6] against the barley genome database led to no hits, indicating these subfamilies are absent or highly divergent in barley, consistent with reports of their absence/loss in monocots [3, 5]. Additionally, searches of the low confidence (LC) gene set led to nine sequence, of which one (MLOC_43388.1) was the longest (S2 Table). However, their putative translation products indicated they were partial and lacked NPA motifs, except two sequences (MLOC_37440.1 and MLOC_43388.1) that exhibited one NPA. The LC genes need confirmation and were not included in further analyses. In summary, the methods collectively led to the barley AQP superfamily comprising of at least forty members, twenty four being annotated and sixteen unannotated (Table 1).

**Table 1:** Summary of the identified barley aquaporins.

Barley AQP	GenBank accession numbers	Corresponding barley CDS from genome database^#^	Corresponding barley gDNA contigs from genome database		Chromosomal location*	Method used to identify aquaporins		
						Barley genome database		mRNA-seq
			Bowman	Morex		gDNA	CDS	
**PIPs**								
HvPIP1;1	AB286964; AK249573; X76911	MLOC_80094	contig_863807	contig_88585	2HL	✓	✓	✓
HvPIP1;2	AB275278	-	contig_72237	contig_95629	5HL	✓	x	✓
HvPIP1;3	AB009308; AK251251	-	-	contig_1569089	6HL	✓	✓	✓
HvPIP1;4	AB275279	-	contig_2001921	contig_280723	6HL	✓	x	✓
HvPIP1;5	AB009309; AK360427; AK359326	MLOC_10855	contig_879787	contig_1559936	6HL	✓	✓	✓
HvPIP2;1	AB219366; AB009307; AK250654	MLOC_13871	contig_64927	contig_1566694	6HL	✓	✓	✓
HvPIP2;2	AB377269; AK250563; AK253017	-	contig_1993266	contig_40687	2HS	✓	✓	✓
HvPIP2;2a	-	MLOC_56278	contig_1993266	contig_40687	2HS	✓	✓	x
HvPIP2;3	AB275280; AK376080; AK353861; AK249631	-	contig_9266	contig_140919	2HL	✓	✓	✓
HvPIP2;4	AB219525; AK252600	-	contig_869787	contig_1616200	2HL	✓	✓	✓
HvPIP2;5	AB377270; AK370379; AK370703	MLOC_54419	contig_65610	contig_39125	2HS	✓	✓	✓
HvPIP2;6	-	MLOC_62649	contig_164695	contig_46809	5HS	✓	✓	✓
HvPIP2;7	GU584120; AK359099; AK359187; AK248491	MLOC_552	contig_396978	contig_103970	5HL	✓	✓	✓
HvPIP2;7a	AK359996	-	contig_396978	contig_103970	5HL	✓	✓	✓
HvPIP2;8	AK359199; AK356299; AB808658	MLOC_44991	contig_16921	contig_275925	5HL	✓	✓	✓
HvPIP2;9	AK361545; AK361542	MLOC_61081	contig_1576537	contig_45084	6HS	✓	✓	✓
HvPIP2;10	AK373720	MLOC_72670	contig_869109	contig_62088	7HS	✓	✓	✓
HvPIP2;11	-	MLOC_17384	contig_282313	contig_1576537	4HL	✓	✓	x
HvPIP2;12	-	MLOC_18325	contig_72035	contig_1580129	5HL	✓	✓	x
**TIPs**								
HvTIP1;1	AB540221; X80266; AK359670; AK367756	MLOC_73301	contig_2001586	contig_63334	4HL	✓	✓	✓
HvTIP1;2	AB540226; **GU584119** ^a^; AK355942; AK372282; AK253104; AK367251	MLOC_58872	contig_844644	contig_43071	3HL	✓	✓	✓
HvTIP2;1	AB540222; AK250814; AK251090	MLOC_66094	contig_9681	contig_51093	6HL	✓	✓	✓
HvTIP2;2	AB540223; AK363660	-	contig_222714	contig_401367	2HL	✓	✓	✓
HvTIP2;3	AB540224; EU872296; **GU584121** ^b^; AB261102; AK248215; AK249965	MLOC_22808	contig_1985016	contig_161234	7HL	✓	✓	✓
HvTIP3;1	AB540228; AK376769	MLOC_51183	contig_16911	contig_368665	1H	✓	✓	✓
HvTIP3;2	AK373620	MLOC_72436	contig_46720	contig_61640	-	✓	✓	✓
HvTIP4;1	AB540225; AK364960; AK368258; AK358374	MLOC_71237	contig_268752	contig_59399	4HL	✓	✓	✓
HvTIP4;2	-	MLOC_71267	contig_12177	contig_5946	3HS	✓	✓	x
HvTIP4;3	-	MLOC_69640	contig_872426	contig_56741	3HS	✓	✓	x
HvTIP5;1	AB540227	-	contig_222714	contig_401367	2HL	✓	x	x
**NIPs**								
HvNIP1;1	AB540230; AK356027	-	contig_848627; contig_135880	contig_2551848; contig_95435	7HS	✓	✓	✓
HvNIP1;2	AB540231; AK365010	MLOC_36500	contig_1982025; contig_75289	contig_2554420; contig_2546891	5HL	✓	✓	✓
HvNIP2;1	GQ496520; AK363953; GQ496519; AB447482; ^c^ **AB710142**	MLOC_67894	contig_1985750	contig_53853	6HL	✓	✓	✓
HvNIP2;2	AB447484; **AB540229** ^d^	-	contig_62067	contig_45067	7HS	✓	x	✓
HvNIP2;3	AK360552; AK357908	-	contig_202862	contig_1633799	-	✓	✓	✓
HvNIP3;1	-	MLOC_64918	contig_71289	contig_49507	1H	✓	✓	✓
HvNIP3;2	-	MLOC_14646	contig_221593	contig_1568583	3HL	✓	✓	x
HvNIP4;1	AK373249	MLOC_62234	contig_15550	contig_46327	3HS	✓	✓	x
**SIPs**								
HvSIP1;1	AK355004; AK252830	-	contig_422844; contig_2006162; contig_889326	contig_157697; contig_27; contig_53238	4HS	✓	✓	✓
HvSIP2;1	AK364835; AK364572	-	contig_1983253	contig_48750	4HL	✓	✓	✓

Barley AQPs annotated in this study are underlined;

^‘✓’^ indicates present and

^‘x’^ indicates absent;

^‘-’^: no information available.

^#^ The corresponding barley CDS was obtained through Blast searches against the IPK Barley Blast Server (http://webblast.ipk-gatersleben.de/barley/) high confidence CDS (HC_genes_CDS_Seq) and full length cDNA databases.

*Chromosomal location data was obtained from the IBGSC barley genome database. The Genbank accession numbers likely to have been currently annotated incorrectly are shown in **bold**.

^a^annotated as HvTIP1;3 instead of HvTIP1;2;

^b^annotated as HvTIP2;1 instead of HvTIP2;3;

^c^annotated as HvNIP2;2 instead of HvNIP2;1;

^d^annotated as HvNIP2;1 instead of HvNIP2;2.

### Phylogenetic analyses, new annotations and predicted intron-exon structures

**Fig 1:**
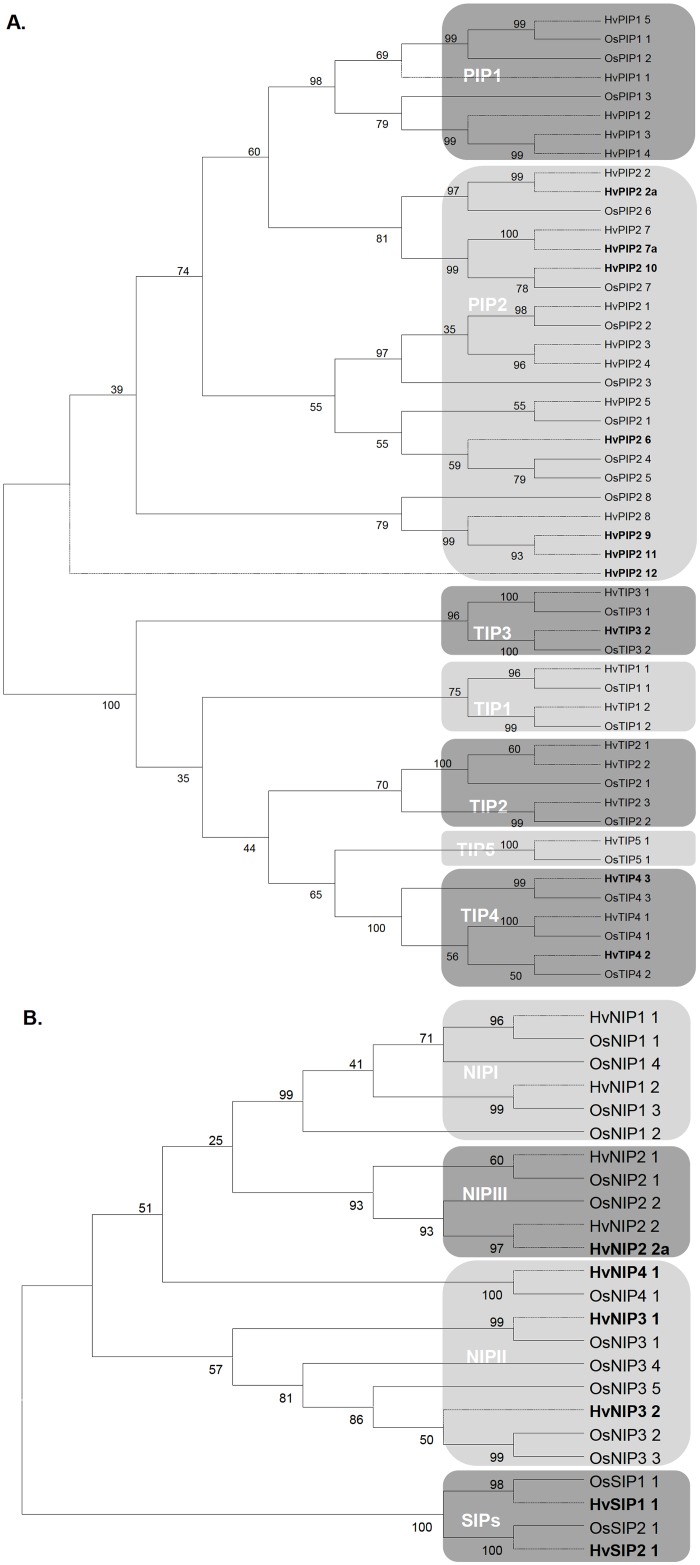
Phylogenetic trees of putative barley and rice aquaporin sequences. A. PIPs and TIPs; B. NIPs and SIPs. The phylogenetic trees were constructed in MEGA4.1 (maximum likelihood with bootstraps) based on the sequence alignments shown in S1 Fig. The different sub-families and groups are highlighted in different shades of grey. The AQPs annotated in this study are shown in **bold**. The branches corresponding to barley AQPs are shown as dashed lines while those for rice are solid lines.

The phylogenetic analysis based on the nucleotide sequence alignments (S1 Fig) showed that the forty AQPs clustered into the four major sub-families (PIPs, TIPs, NIPs, SIPs) in relation to rice orthologs, and that all unannotated PIPs clustered with OsPIP2s (Fig 1). *HvPIP2c* clustered with *OsPIP2;8* and the previously annotated HvPIP2;8 [22] and was annotated as *HvPIP2;9*. A BLASTx of *HvPIP2b* against NCBI identified *Sorghum bicolor SbPIP2;6* (XM_002461888/XM_002461891) as the closest ortholog, hence it was annotated as *HvPIP2;6*, instead of the GU989200 currently annotated so in NCBI. *HvPIP2d* possessed two insertions (115 bp and 116 bp) identical to two introns in *HvPIP2;7* genomic sequence. Likewise, *HvPIP2e* displayed an 86 bp insertion identical to an *HvPIP2;2* intron; hence these were annotated as *HvPIP2;7a* and *HvPIP2;2a*, respectively. The respective translations revealed a frame-shift at amino acid 246, and premature stop codons. Thus these two may be either mis-annotated as cDNA in NCBI and barley genome database, or may undergo alternative splicing, or may be pseudogenes. Based on the closest rice orthologs, other unannotated barley genes were renamed *HvPIP2;10* (*HvPIP2a*), *HvPIP2;11* (*HvPIP2f*), *HvPIP2;12* (*HvPIP2g*), *HvTIP3;2* (*HvTIP3*); *HvTIP4;2* (*HvTIP4b*), *HvTIP4;3* (*HvTIP4a*), *HvNIP3;1* (*HvNIP3a*), *HvNIP3;2* (*HvNIP3b*) and *HvNIP4;1* (*HvNIP4*). *HvNIP2* clustered with *HvNIP2;2* (Fig 1) and the sequences differed at both DNA and amino acid levels, hence *HvNIP2* was annotated as *HvNIP2;3*. *HvSIP1* and *HvSIP2* represent *HvSIP1;1* and *HvSIP2;1*, respectively, of Besse *et al*. [21] (Table 1). Pairwise identity scores were then obtained by alignments of the rice and barley cDNAs and putative protein sequences to identify the closest orthologs (S3 Table).

Comparison of the cDNAs (from leaf mRNA-seq data [19] and IBGSC barley genome project) and the available complete genomic sequences (from IBGSC barley genome project) using GSDS indicated that the HvPIPs had zero to three introns, as noted in rice OsPIPs [8] but unlike the typically three introns in dicot PIPs, e.g., soybean GmPIPs [6] (Table 2; AQPs which lack complete gDNA sequences are not included). The HvPIPs exhibited a diversity in intron sizes (71 to 1,245 bp), while most introns of TIPs were <200 bp (Table 2). Similar to soybean [6], two introns were common for HvTIPs, except HvTIP1s and *HvTIP4;3*. The NIP introns varied greatly in length (79 to 4,765 bp) and NIPIIIs showed the highest intron number (four). All introns displayed the standard GT/AG splice junctions except for the first intron of *HvPIP2;7* and *HvPIP2;10* which exhibited GC/AG.

**Table 2:** Intron-exon structures of the identified barley aquaporin genes.

Barley AQP	Gene length (bp)	Gene Structure										
		Number of		Intron and exon sizes (bp)								
		Exon	Intron	E1	I1	E2	I2	E3	I3	E4	I4	E5
**PIPs**												
*HvPIP1;1*	1,644	4	3	334	101	296	122	141	554	96		
*HvPIP1;2*	1,082	3	2	642	91	141	112	96				
*HvPIP1;4*	1,088	3	2	642	95	141	114	96				
*HvPIP1;5*	2,790	4	3	337	990	296	292	141	635	99		
*HvPIP2;1*	2,974	4	3	318	116	301	1,245	142	741	111		
*HvPIP2;2*	950	2	1	735	86	129						
*HvPIP2;2a*	954	1		954								
*HvPIP2;3*	2,214	3	2	615	895	141	446	117				
*HvPIP2;5*	2,179	3	2	618	1,212	141	94	114				
*HvPIP2;6*	1,076	3	2	609	92	141	123	111				
*HvPIP2;7*	1,107	3	2	307	115	437	116	132				
*HvPIP2;7a*	1,107	1		1,107								
*HvPIP2;8*	876	1		876								
*HvPIP2;9*	879	1		879								
*HvPIP2;10*	1,219	4	3	337	71	299	105	141	128	138		
*HvPIP2;11*	879	1		879								
*HvPIP2;12*	840	1		840								
**TIPs**												
				**E1**	**I1**	**E2**	**I2**	**E3**	**I3**	**E4**	**I4**	**E5**
*HvTIP1;1*	1,146	2	1	133	393	620						
*HvTIP1;2*	880	2	1	133	121	626						
*HvTIP2;1*	951	3	2	127	119	251	82	372				
*HvTIP2;2*	960	3	2	127	86	251	124	372				
*HvTIP2;3*	937	3	2	130	98	248	92	369				
*HvTIP3;1*	1,036	3	2	154	145	248	99	390				
*HvTIP3;2*	1,160	3	2	139	105	248	266	402				
*HvTIP4;1*	1,378	3	2	142	164	254	440	378				
*HvTIP4;2*	1,668	3	2	127	791	251	130	369				
*HvTIP4;3*	851	2	1	127	95	629						
*HvTIP5;1*	984	3	2	127	108	251	87	411				
**NIPs**												
				**E1**	**I1**	**E2**	**I2**	**E3**	**I3**	**E4**	**I4**	**E5**
*HvNIP1;2*	2,300	4	3	306	512	423	95	62	691	211		
*HvNIP2;1*	2,983	5	4	150	110	225	1,427	195	122	62	436	256
*HvNIP2;2*	3,025	5	4	168	91	225	1,365	195	129	62	537	253
*HvNIP3;1*	5,881	4	3	225	4,765	426	104	62	103	196		
*HvNIP3;2*	827	3	2	429	79	62	109	148				
*HvNIP4;1*	1,410	3	2	147	154	416	405	288				
**SIPs**												
*HvSIP2;1*	3,529	3	2	318	2,150	258	629	174				

AQPs where the intron-exon structures could not be determined due to the lack of complete gDNA sequences are not included in the table.

### Characteristic features and potential transport abilities of the putative aquaporins

The putative protein sequences of the barley AQPs showed conservation of both NPA motifs in all isoforms except for HvNIP3;1 which exhibited NPS and NPV, and HvSIP1;1 and HvSIP2;1 exhibited Leu or Thr at the Ala of first NPA (Table 3; S2 Fig). The ar/R substrate selectivity filter residues for PIPs were F-H-T-R, similar to those in maize [7], wheat and rice [8, 10]. The product of HvPIP2;7a lacked both NPAs and had unusual ar/R residues due to the frameshift (see above). The HvTIPs exhibited six different combinations, i.e., H-I-A-V (TIP1s), H-I-G-R (TIP2s), H-I-A-R (TIP3s), Q-T-A-R (HvTIP4;1), H-V-A-R (HvTIP4;3) and Q-V-A-R (HvTIP5;1), confirming the variability in TIPs in other plants [6, 8]. HvNIP2;1 and HvNIP2;2 showed G-S-G-R, similar to other NIPIIIs such as OsNIP2;1 and OsNIP2;2 [8]. The HvNIPI residues were W-V-A-R, characteristic of NIPIs in rice [8] and soybean [6]. The HvNIP3;2 presented a new combination (V-I-A-R). The P1-P5 positions deemed important for discriminating between authentic ‘aquaporins’ and glyceroporins [23], were more conserved at P2-P4 in all subfamilies (Table 3).

**Table 3:** Key structural features and predicted non-aqua transport substrates of putative barley aquaporin proteins.

Barley AQP	NPA motif	Ar/R selectivity filter				P1—P5					Subcellular location	Predicted transport substrate^a^	Rice ortholog (% identity)*	
	LB/LE	TM2	TM5	LE1	LE2	P1	P2	P3	P4	P5			cDNA	Protein
**PIPs**														
HvPIP1;1	NPA/ NPA	F	H	T	R	Q	S	A	F	W	PM	boron, CO_2_, urea	OsPIP1;2 (89.6)	OsPIP1;1 (92.3)
HvPIP1;2	NPA/ NPA	F	H	T	R	Q	S	A	F	W	PM	boron, CO_2_, urea, water ^8^	OsPIP1;3 (85.7)	OsPIP1;3 (89.3)
HvPIP1;3	NPA/ NPA	F	H	T	R	Q	S	A	F	W	PM	boron ^1^, CO_2_, urea	OsPIP1;3 (82.8)	OsPIP1;3 (88.0)
HvPIP1;4	NPA/ NPA	F	H	T	R	Q	S	A	F	W	PM	boron ^1^, CO_2_, urea	OsPIP1;3 (83.2)	OsPIP1;3 (88.6)
HvPIP1;5	NPA/ NPA	F	H	T	R	Q	S	A	F	W	PM	boron, CO_2_, urea	OsPIP1;1 (92.3)	OsPIP1;1 (94.1)
HvPIP2;1	NPA/ NPA	F	H	T	R	Q	S	A	F	W	PM	boron, CO_2_^2; 5^, urea, water ^7^	OsPIP2;2 (87.5)	OsPIP2;2 (88.2)
HvPIP2;2	NPA/ NPA	F	H	T	R	M	S	A	F	W	PM	CO_2_^5^, glycerol, urea, water ^7; 8^	OsPIP2;1 (78.0)	OsPIP2;1 (72.2)
HvPIP2;2a	NPA/ NPA	F	H	T	R	M	S	A	Y	L	PM	glycerol, urea	OsPIP2;6 (78.3)	OsPIP2;6 (75.5)
HvPIP2;3	NPA/ NPA	F	H	T	R	Q	S	A	F	W	PM	boron, CO_2_^5^, urea, water ^7^	OsPIP2;3 (90.2)	OsPIP2;3 (89.3)
HvPIP2;4	NPA/ NPA	F	H	T	R	Q	S	A	F	W	PM	boron, urea, water ^7^	OsPIP2;3 (90.1)	OsPIP2;3 (88.3)
HvPIP2;5	NPA/ NPA	F	H	T	R	Q	S	A	F	W	PM	boron, CO_2_^5^, urea, water^7; 8^	OsPIP2;1 (90.1)	OsPIP2;1 (90.0)
HvPIP2;6	NPA/ NPA	F	H	T	R	Q	S	A	F	W	PM	boron, CO_2_, urea	OsPIP2;4 (51.6)	OsPIP2;4 (51.0)
HvPIP2;7	NPA/ NPA	F	H	T	R	A	S	A	F	W	PM	CO_2_, water ^8^	OsPIP2;7 (75.0)	OsPIP2;7 (77.4)
HvPIP2;7a	-	F	-	-	-	-	-	-	-	-	-	-	OsPIP2;7 (48.5)	OsPIP2;7 (44.1)
HvPIP2;8	NPA/ NPA	F	H	T	R	H	S	A	F	W	PM	-	OsPIP2;5 (71.0)	OsPIP2;5 (68.0)
HvPIP2;9	NPA/ NPA	F	H	T	R	H	S	A	F	W	PM	-	OsPIP2;5 (68.3)	OsPIP2;5 (63.0)
HvPIP2;10	NPA/ NPA	F	H	T	R	M	S	A	F	W	PM	CO_2_, glycerol, urea	OsPIP2;7 (76.9)	OsPIP2;7 (63.8)
HvPIP2;11	NPA/ NPA	F	H	T	R	H	S	A	F	W	PM	-	OsPIP2;1 (68.3)	OsPIP2;3 (66.4)
HvPIP2;12	NPA/ NPA	F	H	T	R	H	S	A	F	W	PM	-	OsPIP2;1 (74.2)	OsPIP2;1 (70.0)
**TIPs**														
HvTIP1;1	NPA/ NPA	H	I	A	V	T	S	A	Y	W	PM/V	H_2_O_2_, urea, water ^8^	OsTIP1;1 (89.3)	OsTIP1;1 (89.6)
HvTIP1;2	NPA/ NPA	H	I	A	V	T	S	A	Y	W	V	H_2_O_2_, urea, water ^8^	OsTIP1;2 (87.3)	OsTIP1;2 (90.8)
HvTIP2;1	NPA/ NPA	H	I	G	R	T	S	A	Y	W	V/PM	ammonia, formamide, H_2_O_2_	OsTIP2;1 (89.3)	OsTIP2;1 (90.7)
HvTIP2;2	NPA/ NPA	H	I	G	R	T	S	A	Y	W	PM	ammonia, formamide, H_2_O_2_	OsTIP2;1 (68.6)	OsTIP2;1 (88.3)
HvTIP2;3	NPA/ NPA	H	I	G	R	T	S	A	Y	W	V/PM	ammonia, formamide, H_2_O_2_, water ^8^	OsTIP2;2 (91.2)	OsTIP2;2 (90.7)
HvTIP3;1	NPA/ NPA	H	I	A	R	T	V	A	Y	W	PM/M	H_2_O_2_	OsTIP3;1 (88.0)	OsTIP3;1 (88.3)
HvTIP3;2	NPA/ NPA	H	I	A	R	S	A	A	Y	W	Chl	H_2_O_2_	OsTIP3;2 (84.1)	OsTIP3;2 (81.3)
HvTIP4;1	NPA/ NPA	Q	T	A	R	T	S	A	Y	W	V/PM	-	OsTIP4;1 (81.1)	OsTIP4;1 (80.9)
HvTIP4;2	NPA/ NPA	H	T	A	R	T	S	A	Y	W	V/PM	glycerol, urea	OsTIP4;2 (75.0)	OsTIP4;2 (70.2)
HvTIP4;3	NPA/ NPA	H	T	A	R	A	S	A	Y	W	PM	-	OsTIP4;3 (63.7)	OsTIP4;3 (52.8)
HvTIP5;1	NPA/ NPA	Q	V	A	R	S	S	A	Y	W	Chl	-	OsTIP5;1 (86.1)	OsTIP5;1 (79.2)
**NIPs**														
HvNIP1;1	NPA/ NPA	W	V	A	R	F	T	A	Y	V	PM	Glycerol, water ^6^	OsNIP1;1 (83.1)	OsNIP1;1 (84.9)
HvNIP1;2	NPA/ NPA	W	V	A	R	F	T	A	Y	I	PM	glycerol, arsenite ^6^ , water ^6^	OsNIP1;3 (69.9)	OsNIP1;3 (67.8)
HvNIP2;1	NPA/ NPA	G	S	G	R	L	T	A	Y	F	PM/ER	antimony, arsenite, boron ^3^, silicon ^4^, urea, water ^6^	OsNIP2;1 (84.0)	OsNIP2;1 (78.9)
HvNIP2;2	NPA/ NPA	G	S	G	R	L	T	A	Y	F	PM/Chl	antimony, arsenite, boron, silicon, urea, water ^6^	OsNIP2;2 (88.5)	OsNIP2;2 (87.0)
HvNIP2;3	NPA/ NPA	G	S	G	R	L	T	A	Y	F	PM/Chl	antimony, arsenite, boron, silicon, urea	OsNIP2;2 (87.6)	OsNIP2;2 (87.7)
HvNIP3;1	NPS/ NPV	A	I	G	R	F	T	A	Y	L	V	antimony, arsenite, boron	OsNIP3;1 (71.6)	OsNIP3;1 (88.5)
HvNIP3;2	NPA/ NPA	V	I	A	R	Y	T	A	Y	L	PM/ER	-	OsNIP3;3 (55.7)	OsNIP3;3 (58.9)
HvNIP4;1 **SIPs**	NPA/ NPA	C	G	G	R	M	S	A	Y	V	PM	-	OsNIP4;1 (72.2)	OsNIP4;1 (59.8)
HvSIP1;1	NPT/ NPA	L	V	P	N	M	A	A	Y	W	ER	-	OsSIP1;1 (83.0)	OsSIP1;1 (85.0)
HvSIP2;1	NPL/ NPA	S	H	G	S	F	A	A	Y	W	PM/V	-	OsSIP2;1 (83.7)	OsSIP2;1 (74.5)

^‘-’^ Indicates no information available.

^a^Potential substrate transported predicted using the signature sequences developed earlier [31]. The underlined substrates have been experimentally proven for the particular barley AQPs: ^1^[48], ^2^[50], ^3^[49], ^4^[51], ^5^[40], ^6^[26], ^7^[13], ^8^[21].

*Only the highest % identity score of each sequence is listed. The identity scores were obtained based on alignments of the cDNAs and putative protein sequences by pairwise identities in BioEdit (S3 Table). The AQPs annotated in this study are shown in **bold**. Sub-cellular locations: Chl (chloroplast), ER (endoplasmic reticulum), M (mitochondria), PM (plasma membrane) and V (vacuole).

The radish RsPIP1s have lower water permeability than RsPIP2s, and a loop E residue in RsPIP1;3 (Ile244) compared to RsPIP2;2 (Val235) is suggested to be relevant [24]. All HvPIP1s possessed Ile while the HvPIP2s had Val (S2 Fig), suggesting the HvPIP1s may have lower water permeability. Further, the difference between OsPIP1s (lower water permeability) and OsPIP2s (higher water permeability) was attributed to a residue in TM2, where OsPIP1s have Ala and OsPIP2s have Ile/Val [25]. At this position, HvPIP1;1 and HvPIP1;5 had Ala, but all HvPIP2s as well as HvPIP1;2, HvPIP1;3, HvPIP1;4 had Val (S2 Fig), suggesting the latter group may be permeable to water and supporting the observation of Besse *et al*. [21] that HvPIP1;2 is permeable to water. Some of the HvTIPs and HvNIPs also possessed a Val here (e.g. HvTIP2;1, HvTIP2;3, HvNIP1;1, HvNIP1;2), suggesting this residue may be functionally important for water transport of TIPs and NIPs. In line with this suggestion, a recent study observed that HvNIP1;1, HvNIP1;2, HvNIP2;1 and HvNIP2;2 all of which possessed the Val residue in TM2 (S2 Fig), were water transporters [26].

AQPs are known to function as tetramers and a highly conserved Cys (Cys80 in ZmPIP2;1) in loop A of PIP1s and PIP2s is shown to be essential for formation of disulphide bonds between PIP monomers, increasing the oligomer stability [27]. Alignments showed conservation of this Cys in all HvPIP1s and PIP2s (S2 Fig), suggesting a similar role for it in these, but its absence in TIPs, NIPs and SIPs suggests other residue(s) may be involved in this role. All PIPs were predicted to localise to the plasma membrane, as the subfamily name suggests and consistent with localisation of HvPIP2;1 [12]. However, an N-terminal diacidic motif Asp-Ile/Val-Glu [28, 29] found important for exit of newly synthesised PIPs from the ER to plasma membrane was noted only in HvPIP2;1, HvPIP2;3 and HvPIP2;4. Further, the subfamily names do not always indicate location (reviewed in [8]). In congruence with this, the TIPs were predicted to localise to the plasma membrane, mitochondria and chloroplast, in addition to the expected tonoplast (Table 3). Some NIPs were predicted to localise to the plasma membrane and HvSIP1;1 was predicted in the endoplasmic reticulum. All HvPIPs except HvPIP2;7 and HvPIP2;10 possessed a His corresponding to His193 in SoPIP2;1 considered important for pH-dependent gating [30] (S2 Fig), but the TIPs, NIPs and SIPs did not show this residue. Gating of SoPIP2;1 by phosphorylation of Ser115 and Ser274 has been shown, with blocking by Leu197 [30]. All three equivalent residues were observed for most HvPIPs. However, most TIPs possessed a Thr at Ser115, lacked Ser274, and only in HvTIP4;3 and HvTIP5;1 showed Leu197. All NIPs (except HvNIP3;2) had a Ser at Ser262 in GmNOD26 involved in phosphorylation. Alignments with AtPIP2;1 showed a conserved Ser in most HvPIP2s corresponding to S280 and S283, which are phosphorylated under salt stress [31] (S2 Fig).

Our earlier analysis of plant AQPs with non-aqua transport substrates had revealed distinct signature sequences and ‘specificity determining positions’ (SDPs) for each substrate group [32]; hence the putative barley AQPs were analysed for these. A number of isoforms were predicted to have the potential to transport micronutrients and molecules involved in plant growth and vigour (boron also being toxic in high concentrations) (Table 3). The potential to transport urea (many PIPs, TIPs and NIPs) and boron (many HvPIPs but no TIPs) was widespread, but that for ammonia was restricted to some TIPs (HvTIP2;1 HvTIP2;2; HvTIP2;3), CO_2_ to HvPIPs (all except HvPIP2;7), and silicon to NIPs (HvNIP2;1, HvNIP2;2; HvNIP2;2). The potential to transport the signalling molecule H_2_O_2_ seemed restricted to the TIPs (Table 3). NIPs had the most diverse predicted substrates, as found in other studies, including the potentially toxic arsenite.

### Modulation of expression of barley aquaporin genes

In mRNA-seq, gene expression level is generally measured as the number of sequence reads mapping to a gene [33]. In the present study, the normalised sequence reads (base mean expression) were used. Most PIPs and some TIPs exhibited higher expression levels than NIPs and SIPs (S3 Fig), in line with Arabidopsis [34]. *HvPIP1;4* had the most abundant transcripts in control plants, which nearly doubled upon exposure to salt stress (fold change (FC) +1.93; S1 Table). FC of ≥ +1.5 or ≤ -1.5 is considered notable [35], hence in the present context, such genes may have roles in osmotic regulation under salinity stress. Several other *PIP*s also showed notable changes, the largest being noted for *HvPIP1;3* (FC +2.93) but that in *HvPIP1;2* (FC +1.97) being statistically significant. Among the TIPs, *HvTIP1;2* and *HvTIP4;1* were the most abundant AQP transcripts in control plants, but only *HvTIP4;1* displayed a statistically significant decline (FC -15.89) under salinity, suggesting a critical role for it. *HvTIP1;1* and *HvTIP2;3* showed moderate expression and up-regulation (FC +1.62, +2.60, respectively). *HvTIP2;1*, *HvTIP2;2*, *HvTIP3;1* and *HvTIP3;2* exhibited very low expression levels, but *HvTIP3;2* was significantly up-regulated (FC +2.40). *HvNIP2;2* was the most abundant NIP, but expressed at much lower levels than many PIPs and TIPs, and showed little change. The five other NIPs exhibited low-moderate expression and only *HvNIP2;1* showed notable down-regulation. *HvSIP2;1* had higher and more differential expression than *HvSIP1;1*. In summary, thirteen genes showed up-regulation and five showed a decrease under salinity, of which the changes in *HvTIP4;1* and *HvPIP1;2* were statistically significant (p-value <0.05).

The ESTs corresponding to the forty genes above were analysed and grouped as follows; apex, callus, epidermis, flower (anther, carpel, inflorescence and pistil), leaf, maternal, root, seed (caryopsis, coleoptile, embryo, endosperm, pericarp, testa), shoot, and spike (rachis) (S4 Table). Based on the total number of ESTs, the PIPs (2,043) and TIPs (1,260) appeared to have much higher expression than the NIPs (51) and SIPs (40), confirming other reports [34]. The PIPs and TIPs also typically showed higher expression in the leaf (PIPs: 285; TIPs: 112), root (546; 305), seed (215; 417) and shoot (798; 281) compared to other tissues (S4 Fig). Some ESTs were noted in response to stresses such as cold, drought, salinity or waterlogging (S5 Table). The heat-map generated for the IBGSC RNA-seq data of eight tissues related to the high-confidence gene predictions supported high expression levels of PIPs and TIPs in shoots (including tillers), inflorescences and developing grain, but not roots and germinating seed (S5 Fig). HvTIP1;1, HvTIP2;1, HvTIP2;3 and HvPIP1;1 were highly expressed in a number of tissues. In many tissues the expression levels of NIPs were lower than or the same as the TIPs and PIPs, except for high levels of HvNIP3;1 (developing grain) and HvNIP4;1 (inflorescence). A number of isoform-specific patterns were also noted.

Further, microarrays have been employed to monitor gene expression under salt stress in barley cv. Maythorpe and Golden Promise, the latter being more effective in its Na+ exclusion ability [36]. Analysis of this data in Genevestigator demonstrated that ten sequences (*HvPIP1;1*, *HvPIP1;5*, *HvPIP2;2*, *HvPIP2;5*, *HvTIP1;1*, *HvTIP2;3*, *HvNIP1;1*, *HvNIP1;2*, *HvNIP2;3*, *HvNIP4;1*) showed similar trends (up- or down-regulation) in both cultivars, but others differed (Table 4; S1 Table). Differences were also noted in the expression of some isoforms between the root and shoot of a variety (e.g., *HvPIP2;1*, *HvPIP2;4* of Golden Promise). Further, comparison of the microarray data (cv. Maythorpe and Golden Promise) to leaf mRNA-seq (cv. Hindmarsh) indicated inter-cultivar differences in expression of many isoforms, while eight genes showed a common trend (e.g., *HvPIP2;1*, *HvTIP1;2*, *HvNIP1;1*). This may be due to differences in the sensitivity of the techniques, innate inter-cultivar differences, and/or experimental factors (Golden Promise and Maythorpe salt-stressed for 5 days; Hindmarsh for 12 hours). However, *HvTIP4;1* exhibited the largest change (Maythorpe shoot FC -2.50, Hindmarsh FC -15.89), reinforcing its importance.

**Table 4:** Analysis of reported microarray data for expression of barley aquaporins under salinity stress.

Barley AQP	HarvEST Unigene No.^	Probeset ID	Response to salinity stress			
			Golden Promise		Maythorpe	
			Shoot	Root	Shoot	Root
**PIPs**						
HvPIP1;1	13831	Contig1225_s_at; HW09I11u_s_at	-1.43	**-1.51**	**-1.79**	**-1.50**
HvPIP1;2	13853	Contig1228_s_at	-1.10	-1.16	+1.28	-1.28
HvPIP1;3	13837	Contig1239_s_at	-1.14	-1.00	+1.16	+1.02
HvPIP1;4	13845	Contig1219_s_at**	-1.08	-1.00	+1.19	-1.19
HvPIP1;5	13757	Contig1230_at**	-1.10	-1.26	-1.22	**-1.52**
HvPIP2;1	13861	Hv08C12u_x_at**	+1.03	-1.05	+1.01	+1.02
HvPIP2;2	13833	Contig1216_s_at	-1.17	-1.31	-1.25	**-1.53**
HvPIP2;3	13857	Contig1223_at	+1.40	+1.03	**+1.95**	-1.12
HvPIP2;4	13852	EBem09_SQ003_F16_s_at	+1.06	-1.00	+1.40	-1.08
HvPIP2;5	13825	Contig1222_s_at	**-1.50**	-1.40	-1.42	**-1.74**
HvPIP2;6	13694	-	-	-	-	-
HvPIP2;7	8763	Contig19393_at	**+1.99**	-1.00	+1.10	-1.00
HvPIP2;8	13870	-	-	-	-	-
HvPIP2;9	43384	HV_CEb0007N06r2_at	-1.22	+1.01	-1.08	-1.03
**TIPs**						
HvTIP1;1	14110	HS07J06u_s_at	-1.08	-1.09	-1.13	-1.14
HvTIP1;2	14114	HVSMEf0019H18r2_at	+1.04	-1.15	+1.33	-1.18
HvTIP2;1	14113	Contig1310_at**	+1.24	**-1.55**	+1.05	**-1.53**
HvTIP2;2	14105	Contig1308_at	+1.13	**-1.76**	+1.10	**-1.87**
HvTIP2;3	14109	Contig1315_s_at	-1.28	**-1.65**	-1.15	**-1.81**
HvTIP3;1	1488	Contig3772_at; HT03K14r_s_at	+1.15	+1.07	+1.01	-1.00
HvTIP3;2	-	EBem10_SQ003_I02_at	-1.17	-1.04	+1.08	+1.08
HvTIP4;1	16370	Contig7377_s_at	**+1.70**	**+1.54**	**-2.50**	-1.08
HvTIP4;3	38155	HF03B07r_at	+1.07	+1.08	+1.11	-1.01
HvTIP5;1	28056	AF254799_CDS-2_at	+1.04	-1.09	-1.02	+1.07
**NIPs**						
HvNIP1;1	5627	Contig14229_at	+1.04	+1.03	+1.02	+1.11
HvNIP1;2	23039	Contig19214_at	+1.17	**+1.50**	+1.09	**+1.50**
HvNIP2;1	31860	-	-	-	-	-
HvNIP2;2	16339	Contig5632_at Contig5632_s_at	-1.07	-1.28	+1.22	**-1.50**
HvNIP2;3	16340	Contig5634_at	+1.17	+1.07	+1.23	+1.16
HvNIP3;1	7157	Contig16901_at	-1.12	+1.42	+1.05	+1.33
HvNIP4;1	-	Contig19489_at	+1.20	+1.09	+1.07	+1.04
**SIPs**						
HvSIP1;1	2171	Contig6340_at	-1.15	+1.29	+1.10	+1.24
HvSIP2;1	-	Contig19630_at	-1.21	+1.05	-1.20	-1.07

Microarray data obtained from Genevestigator (https://www.genevestigator.com/gv/plant.jsp).

^Unigene number obtained from HarvEST v.1.83;

**Probeset IDs obtained from [21], other probeset IDs acquired from HarvEST: barley. ‘-’ indicates no information available.

FCs indicative of notable up or down regulation (FC ≥ +1.5) are shown in bold, FCs indicative of no differential expression are shown in normal text. AQPs lacking microarray data are not shown.

## Discussion

In recent years, the function of plant AQPs has been extended from transport of water alone to the transport of other substrates and responding to diverse physiological processes and environmental stresses (reviewed in [1]). However, while significant information exists on the AQP families in rice and soybean, a complete picture of this family is missing in barley and wheat, two of the most important cereal crops. In this work, we address this gap by providing comprehensive analysis of what we believe is the full gene set of the barley AQPs superfamily, including new annotations of sixteen transcripts, removal of some redundancies and possible inaccuracies of four annotations (HvNIP2;1, HvNIP2;2, HvTIP1;2 and HvTIP2;3) (Table 1). The annotation of HvNIP2;1 may be regarded as somewhat equivocal, as the sequences AB447482 (registration 14-JUL-2008; Ma, direct submission) and GQ496520 (18-AUG-2009; Sutton *et al*., direct submission) were annotated as HvNIP2;1, followed by registration of AB540229 (13-JAN-2010; Ligaba *et al*., direct submission;) also as HvNIP2;1. However, AB540229 is 100% identical to AB447484 and not to the former two. Our annotations of HvNIP2;1 and HvNIP2;2 are based on the dates of registration as well as % identities to the other annotated barley sequences (listed in Table 1) and to the orthologs in rice [37], maize [7] and/or sorghum (SbNIP2;1 - EF373651; SbNIP2;2 - EF408053). Hence we suggest that AB540229 represents HvNIP2;2, and likewise, AB710142 (annotated as HvNIP2;2; 02-APR-2012, Shibasaka *et al*, direct submission; also see [26]) represents HvNIP2;1. These suggestions are open to further reports and/or Genbank updates. In all, forty genes (nineteen PIPs, eleven TIPs, eight NIPs, two SIPs) were identified, making a significant advancement on the twenty-three (ten PIPs, eight TIPs, three NIPs, two SIPs) estimated by Katsuhara and Hanba [11] and twenty-five (eleven PIPs, eight TIPs, four NIPs, two SIPs) estimated by Besse *et al*. [21]. This family size is consistent with maize (36; [7]), rice (38; [8]) and soybean (66; [6]). Subject to confirmation, nine more sequences identified in the low confidence dataset may also belong. The AQP diversity in plants is attributed to the higher degree of compartmentalization of plant cells and the ability to fine-tune water control *in situ*, under different environmental conditions [34]. The gene identifications and analyses are also relevant to wheat, a genetically close relative; e.g., a previous study could not amplify TIP5 from wheat, with primers based on rice TIP5 [10]; the data on HvTIP5 will address this limitation. A wheat NIP is reported to be involved in salinity tolerance [38]; the present work will enable analysis of its orthologue in barley.

The regulation of AQPs under salinity stress has been well-reported (reviewed in [1, 8]). In barley, response to salinity has been studied mainly in PIPs [11–13]. The present study is unique in targeting all leaf AQPs together. Eighteen of the forty genes showed modulations considered meaningful (FC >1.5; [35]) (S1 Table). The changes in *HvTIP4;1* and *HvPIP1;2* were statistically significant (p-value < 0.05; S1 Table), indicative of their key roles in stress response, particularly noteworthy *HvTIP4;1* which exhibited the largest change. The expression of *HvPIP1;2* in the leaf was unclear in other studies (EST data, S4 Table; [21]). However, this isoform was found significantly up-regulated (S3 Fig; S1 Table), and predicted to be a CO_2_ transporter (Table 3). Pérez-López *et al*. [39] demonstrated that elevated CO_2_ moderates the effects of oxidative stress caused by salinity in barley. Therefore a surge in *HvPIP1;2* may be protective, and other predicted (HvPIP1;1–1;5; HvPIP2;6, HvPIP2;7, HvPIP2;10; current study) and confirmed (HvPIP2;1–2;3, HvPIP2;5; [40]) CO_2_ transporters may also have similar roles. Their genes and expression patterns thus need to be compared in tolerant versus sensitive lines.

The isoform-specific response of barley AQPs noted here support the observations in maize [41]. Tissue-specific responses were also observed, e.g., *HvPIP1;2* was down-regulated in the root [11, 13], but up-regulated in leaf mRNA-seq (S3 Fig; S1 Table). Differences were also noticed in results from different techniques; e.g., *HvPIP2;1* was found largely unchanged in the microarray (Table 4), but down-regulated in roots [12, 13] and up-regulated in shoots by real-time PCR [12] and leaf mRNA-seq [19] and in IBGSC shoot + tillers RNA-Seq data (S5 Fig). In addition, the response of aquaporins to stimuli is often species-dependent. For example, PIP1;1 from rice was shown to be down-regulated in response to 150 mM NaCl [42], while its ortholog in barley (HvPIP1;1) was up-regulated (our current study). The expression of AQPs during water stress is reported to be dependent on cultivars [43] as well as time course and intensity of stress [41]. Thus investigations of wider germ-plasm for allelic differences are essential for assessing the genetic versus environmental factors (including experimental techniques) to such studies.

An N-terminal diacidic motif Asp-Ile-Glu in ZmPIP2;4 and ZmPIP2;5 [28] or Asp-Val-Glu in AtPIP2;1 [29], found important for exit of PIPs from the ER to plasma membrane, was noted in HvPIP2;1, HvPIP2;3 and HvPIP2;4, suggesting they may use this mechanism. It is unclear how the other HvPIPs lacking this motif but also predicted to be plasma membrane-located would be exported. For PIP1s, hetero-tetramer formation with PIP2s has been suggested as means of ER export [28]. The plant cell vacuole is associated with turgor regulation, osmotic adjustment, storage, pH regulation, cell signalling and protein degradation. HvTIP4;1 was predicted to be vacuolar (Table 3), similar to AtTIP4;1 [44]. OsTIP4;1 has been confirmed to be water-permeable and its transcripts fluctuate, possibly in response to turgor status [45], and the orthology suggests that HvTIP4;1 may play a similar role. In addition, other TIPs predicted to be localised to the vacuole could also play this role.

AQP activity can be regulated transcriptionally, and/or post-translationally by pH, phosphorylation and vesicle trafficking [1]. Protonation of a His in loop D of PIPs is shown to be important for pH-dependent gating during flooding (e.g., His 193 in SoPIP2;1; [30]). All HvPIPs except HvPIP2;7 and HvPIP2;10 possessed a His corresponding to His193 (S2 Fig), but the TIPs, NIPs and SIPs did not, suggesting HvPIPs may be regulated by pH, but not other isoforms. Tornroth-Horsefield *et al*. [30] also demonstrated the gating of SoPIP2;1 by phosphorylation of Ser115 and Ser274, with Leu197 as the key blocking residue. The equivalents of Ser115, Ser274, and Leu197 in most HvPIPs (S2 Fig) suggest a similar regulation. In contrast, most TIPs lacked these Sers, and Leu197 occurred only in HvTIP4;3 and HvTIP5;1, suggesting the gating may not apply. The Ser at Ser262 in GmNOD26 [46] was noted in all NIPs (except HvNIP3;2) suggesting their phosphorylation-mediated regulation also. The regulation of AQPs is critical in maintaining the plant water status during normal and stress conditions, and this ability could be employed in the development of transgenic plants. AtPIP2;1 is mono- and di-phosphorylated at two C-terminal sites, S280 (i.e. S274 of SoPIP2;1) and S283 under salt stress [31]. Alignments with AtPIP2;1 showed a conserved Ser at S280 and S283 in most HvPIP2s (S2 Fig), suggesting that these, although not exhibiting transcriptional modulations under salt stress, may be regulated post-translationally.

In recent years, plant AQPs, predominantly NIPs, have been shown to be permeable to twelve different substrates, many of which are of physiological significance, e.g., ammonia and CO_2_ [1], and the signalling molecule hydrogen peroxide [47], but some are toxic, e.g., arsenite. Hence, the involvement of the barley AQPs in transporting any non-aqua substrates was predicted using the specificity determining positions identified earlier [32]. Based on these predictions, a number of PIP isoforms have the potential to transport boron, CO_2_ and urea which are important for plant development (boron also being toxic in high concentrations), the TIPs share the urea and ammonia transport potential but not CO_2_, and transport of the signalling molecule H_2_O_2_ seems restricted to TIPs (Table 3). Some of these are experimentally proven substrates, e.g., boron for HvPIP1;3 and HvPIP1;4 [48] and HvNIP2;1 [49]; CO_2_ for HvPIP2;1–2;3 and HvPIP2;5 [50, 40]; arsenite for HvNIP1;2 [26], and silicon for HvNIP2;1 [51]. Many NIPs appear to have the potential to transport diverse substrates including the micronutrient silicon, but also the toxic arsenite. Further, in our previous work [32] the transport of non-aqua substrates was predicted to generally compromise the water permeability of such AQPs; however, this did not seem to apply to AtTIP1;1, AtTIP2;1, AtNIP1;1, HvPIP2;1, OsPIP2;4, OsPIP2;6, OsPIP2;7, OsTIP1;2, PtNIP1;1 and TaTIP2;2. Thus, their orthologs in barley may also be water transporters.

*HvPIP1;3* was found up-regulated in response to boron, while *HvPIP1;4* was unchanged [48]. In the leaf mRNA-seq data, both of these were up-regulated under salinity stress (S3 Fig; S1 Table). Down-regulation of HvNIP2;1 is suggested to induce boron tolerance [48]; this isoform was found down-regulated by salinity in our study [19]. Orthologs often retain their function during evolution; thus HvNIP2;1 may transport the substrates shown for OsNIP2;1, i.e., boron, silicon and urea [52] and arsenite and antimony [53], and may not necessarily show significant differential expression under salinity stress (alone) (see below). Boron toxicity and salinity both significantly reduce crop yield [54], and may occur together, e.g., in parts of Western Australia. Thus isoforms such as HvPIP1;3 and HvNIP2;1 would be invaluable for identification/development of lines tolerant to both stresses. HvPIP1;1, HvPIP1;2 and HvPIP2;1 are also of interest due to being salt-responsive and possible boron transporters, and HvPIP1;5, HvPIP2;3 HvPIP2;5, HvNIP2;2 and the newly identified HvNIP3;1 are also likely boron transporters. Urea is abundant in nature and application of urea fertilisers is a common agricultural practice; hence there is a need for plants to ‘load and unload’ urea [44], and some of the barley isoforms may be urea transporters. Silicon is found to alleviate abiotic and biotic stresses and improve light interception and canopy photosynthesis, and HvNIP2;1 and HvNIP2;2 being silicon transporters ([55] and within) was supported by our predictions. Isoforms that transport antimony or arsenite could be valuable in their detoxification. The rice orthologs could assist in testing the functions of barley genes, and the sequences and physical locations of barley genes are important for marker development and studying wheat orthologs.

## Conclusions

Forty AQPs is the largest number reported for barley so far. The data acquired on their coding sequences, innate tissue and/or cultivar specific variations in expression, modulations under salinity, and the potential roles of various genes in transport of other substrates and in salinity and other stress responses, have collectively yielded a wealth of molecular information. This will be vital for assessing and/or transgenically developing germplasm of barley and possibly of wheat with improved abiotic stress tolerance and yield.

## Supporting Information

S1 Fig Alignments of barley and rice nucleotide sequences. (DOCX)

S2 Fig Alignments of putative aquaporin protein sequences of barley. (DOCX)

S3 Fig Expression levels of barley aquaporins from leaf mRNA-Seq data. (DOCX)

S4 Fig Summary of ESTs of aquaporin sub-families in different tissue types. (DOCX)

S5 Fig Heat map of expression levels of aquaporins from the barley genome project RNA-seq data for 8 tissues. (DOCX)

S1 Table Barley aquaporins identified from leaf mRNA-seq data. (DOCX)

S2 Table AQP-like sequences identified from the low confidence gene set of the IBGSC barley genome project. (DOCX)

S3 Table Pairwise identities between barley and rice aquaporins. (DOCX)

S4 Table Tissue types of ESTs representing barley aquaporins. (DOCX)

S5 Table Treatment conditions of ESTs representing the barley aquaporins. (DOCX)
