# Supplementary material for: Identification and Expression Analysis of the Barley (Hordeum vulgare L.) Aquaporin Gene Family
Source: PLoS One. 2015 Jun 9;10(6):e0128025. doi: 10.1371/journal.pone.0128025 (PMC4461243; doi:10.1371/journal.pone.0128025)
Supplement: S1 Fig — (DOCX) [file pone.0128025.s001.docx]

**A. PIPs**

10 20 30 40 50 60 70 80 90 100 110 120

....|....|....|....|....|....|....|....|....|....|....|....|....|....|....|....|....|....|....|....|....|....|....|....|

**HvPIP1;1**  ATGGAGGGCAAGGAGGAGGACGTGCGCCTCGGGGCGAACAAGTACTCGGAGCGGCAGCCCATCGGCACGGCGGCGCAGGGGTCCG------AGGACAAGGACTACAAGGAGCCCCCGCCG

**HvPIP1;2**  ATGGAGGGCAAGGAGGAGGACGTGCGTCTGGGCGCGAACCGGTACTCGGAGCGGCAGCCGATTGGGACGGCGGCGCAGGGCGGCGGCGCGGACGAGAAGGACTACAAGGAGCCGCCGCCG

**HvPIP1;3**  ATGGAGGGCAAGGAGGAGGACGTGCGCCTGGGCGCGAACCGCTACTCGGAGCACCAGCCCATCGGCACGGCGGCGCAGGGCGGCGGAGCCGACGAGAAGGACTACAAGGAGCCGCCCCCG

**HvPIP1;4**  ATGGAGGGCAAGGAGGAGGACGTGCGCCTTGGCGCGAACCGCTACTCGGAACGCCAGCCGATCGGCACGGCGGCGCAGGGCGGCGGCGCCGACGAAAAGGACTACAAGGAGCCGCCCCCG

**HvPIP1;5**  ATGGAGGGGAAGGAGGAGGACGTGCGGCTGGGCGCCAACAGGTACTCGGAGCGGCAGCCCATCGGGACGGCGGCGCAGGGCGGCGGGG---ACGACAAGGACTACAAGGAGCCGCCGCCG

**HvPIP2;1**  ------------------------------------------ATGGCCAAGGACATTGAGGCG---GCACCCC---AAGGGGGCGAGTTCTCCAGCAAGGACTACTCGGACCCGCCCCCG

**HvPIP2;2**  ---------------------------------------ATGGCCAAGGAGGTGAGCGAGGAGCCGGAGCACGCCGCGC-------CGGCGCGCAAGGAC--TACTCGGACCCGCCGCCG

**HvPIP2;3**  ------------------------------------------ATGGCCAAGGACATCGAGGCGGCGCCCCCAG------GCGGCGAGTACGGGGCCAAGGACTACTCCGACCCGCCGCCG

**HvPIP2;4**  ------------------------------------------ATGGCCAAGGACATCGAGGCGGCGCCACCCG------GCGGGGAGTACGCGGCCAAGGACTACTCCGACCCGCCGCCG

**HvPIP2;5**  ------------------------------------------ATGGCGAAGGACGAGGTGATGGAGACCGGCGGCGGCG---GCGACTTCGCGGCCAAGGACTACACGGACCCGCCGCCG

**HvPIP2;6**  ------------------------------------------ATGGGCAAAGAGGTGGACGTGTCTGCCCTCGAGGCCG---GCGGC------GCCCGGGACTACTCCGACCCTCCACCG

**HvPIP2;7**  ---------------------------------------ATGTCCAAGGAGGAGGTGATCGCCGGAGGCGACACGGCGGACGTGGCCGTGGAGAAGGCGCCGTACTGGGACCCGCCGCCG

**HvPIP2;10**  ------------ATGCCGTCGCCGATCTTGCCGGCAAAGGAGGTGGAGGAGGTGGTCACCGCCAACGAAGAGGTGACAGACATTATCGTCCAGAGGGTGCCGTACTGGGACCCTCCGGCG

**HvPIP2;8**  ---------------------ATGACTATGGCCGCAGCACAAGGCAAGCTGAGTCCGGATGCCATCGACAACGAAGTCATCAGCAACGGCAGCGCCAAGGACTACCTCGACCCTCCTCCG

**HvPIP2;9**  ---------------------------ATGGCCGCTGCACA--GCAGGGCAAGCATAGTAGA----GACG------CCCATGGTAGCAACGACACCAAGGACTACCTCGACCCTCCCGCG

**HvPIP2;7a**  ---------------------------------------ATGTCCAAGGAGGAGGTGATCGCCGGAGGCGACACGGCGGACGTGGCCGTGGAGAAGGCGCCGTACTGGGACCCGCCGCCG

**HvPIP2;11**  ---------------------------ATGGCCGCTGGAGATGGCAAGCTGAATACGGAGGGC---AACGTTAGTTCCGATACTACCATGAGCACCAAGGACTATCTGGACCCTCCTCCG

**HvPIP2;12**  ---------------------------------------------------------------------ATGGTGCCCAACAACAACACCGTCGACAAGGACTACCGTGACCCTCGCCCG

**HvPIP2;2a**  ---------------------------------------ATGGCCAAGGAGGTGAGCGAGGAGCCGGAGCACGCCGCGC-------CGGCGCGCAAGGAC--TACTCGGACCCGCCGCCG

**OsPIP1;1**  ATGGAGGGGAAGGAGGAGGACGTGCGGCTGGGGGCGAACAGGTACTCGGAGAGGCAGCCGATAGGGACGGCGGCGCAGGGCGCCGGGG---ACGACAAGGACTACAAGGAGCCGCCGCCG

**OsPIP1;2**  ATGGAGGGGAAGGAGGAGGATGTCCGGCTGGGAGCCAACAAGTTCTCGGAGAGGCAGCCGATCGGCACGGCGGCGCAGGGCTCCG------ACGACAAGGACTACAAGGAGCCGCCGCCG

**OsPIP1;3**  ATGGAGGGGAAGGAGGAGGATGTGAGGCTGGGGGCGAACAGGTACACGGAGAGGCAGCCGATCGGGACGGCGGCGCAGGG-GGCGGAG-----GAGAAGGACTACCGGGAGCCGCCGGCG

**OsPIP2;1**  ------------------------------------------ATGGGGAAGGACGAGGTGATGGAGAGCGGCGGCGCCGCCGGCGAGTTCGCGGCCAAGGACTACACGGACCCGCCGCCG

**OsPIP2;2**  ------------------------------------------ATGGCGAAAGACATTGAGGCGTCGGCGCCGG---AGGGCGGCGAGTTCTCGGCGAAGGACTACACCGACCCGCCGCCG

**OsPIP2;3**  ------------------------------------------ATGGCGAAGGACATTGAGGCGGCGGCGGCGGCGGAGGGCGGGGAGTACATGGCGAAGGACTACTCCGACCCGCCGCCG

**OsPIP2;4**  ------------------------------------------ATGGGCAAAGAGGTGGACGTGTCCACTCTCGAGGCCG---GCGGC------GCCCGGGACTACATCGACCCGCCGCCG

**OsPIP2;5**  ------------------------------------------ATGGGCAAAGAGGCCGACGT---------CGAGGCCG---GCGGC------GTCCGGGACTACGAGGACCCGCCGCCG

**OsPIP2;6**  ---------------------------------------ATGTCGAAGGAGGTGAGCGAGGAGCCGGAGCACGT-GCG------------GCCCAAGGAC--TACACCGACCCGCCGCCG

**OsPIP2;7**  ---------------------------ATGGCGTCGAAGGAGGAGGTGGCCGTGGAGACGGTGGAGGGCGGAGCGGCGG-------CGGC--GAAGGCGCCGTACTGGGACCCGCCGCCG

**OsPIP2;8**  ------------------------------------------------------ATGGCTGCA---GGCA------GCGGCAGCGGCAGCAATCCTAAGGACTACCAGGATCCTCCTCCC

130 140 150 160 170 180 190 200 210 220 230 240

....|....|....|....|....|....|....|....|....|....|....|....|....|....|....|....|....|....|....|....|....|....|....|....|

**HvPIP1;1**  GCGCCGCTGTTCGAGCCCGGCGAGCTCAAGTCGTGGTCCTTCTACCGCGCCGGCATCGCCGAGTTCATGGCCACCTTCCTCTTCCTCTACGTCACCATCCTCACCGTCAT-GGG---CTA

**HvPIP1;2**  GCACCGCTGTTCGAGGCGGAGGAGCTGACGTCGTGGTCCTTCTACCGCGCGGGGATCGCCGAGTTCCTGGCCACCTTCCTGTTCCTGTACATCAGCGTGCTCACGGTGAT-GGGCGTGGT

**HvPIP1;3**  GCCCCCTTCTTCGAGGCCGGGGAGCTCACCTCCTGGTCCTTCTACCGGGCAGGGATCGCCGAGTTCCTGGCCACCTTCCTCTTCCTCTACATCTCGGTGCTCACCGTGAT-GGGCGTGGT

**HvPIP1;4**  GCGCCGCTCTTCGAGGCGGAGGAGCTCTCCTCCTGGTCCTTCTACCGGGCAGGGATCGCCGAGTTCCTGGCCACCTTCCTCTTCCTCTACATCTCGGTGCTCACCGTGAT-GGGCGTGGT

**HvPIP1;5**  GCGCCGCTCTTCGAGCCCGGGGAGCTCAAGTCCTGGTCCTTCTACCGCGCCGGCATCGCCGAGTTCATCGCCACCTTCCTCTTCCTCTACGTCACCGTGCTCACCGTCAT-GGG---CGT

**HvPIP2;1**  GCGCCGATCGTGGACTTCGAGGAGCTGACCAAGTGGTCGCTGTACCGCGCGGTGATCGCCGAGTTCGTCGCCACGCTCCTCTTCCTCTACATCACCGTGGCCACCGTCATCGGGTACAAG

**HvPIP2;2**  GCGCCGCTGTTCGACATGGGGGAGCTCCGGATGTGGTCCTTCTACCGGGCGCTCATCGCCGAGTTCGTCGCCACGCTGCTCTTCCTCTACATCACCGTCGCCACCGTCATCGGCTACAAG

**HvPIP2;3**  GCGCCGCTCTTCGACGCGGAGGAGCTGACCAAGTGGTCCCTGTACCGCGCGGTGATCGCCGAGTTCGTGGCCACGCTGCTCTTCCTCTACATCACCGTGGCCACCGTCATTGGGTACAAG

**HvPIP2;4**  GCGCCGCTCTTCGACGCCGAGGAGCTCACCAAGTGGTCCCTGTACCGCGCGGTGATCGCCGAGTTCGTGGCCACGCTGCTCTTCCTCTACATCACCGTGGCCACCGTCATCGGGTACAAG

**HvPIP2;5**  GCCCCGCTGGTGGACGCGGCGGAGCTGGCGTCGTGGTCGCTGTACCGCGCCGTCATCGCCGAGTTCATCGCCACGCTGCTCTTCCTCTACATCACCGTGGCCACGGTCATCGGGTACAAG

**HvPIP2;6**  GCGCCTCTTGTGGACGTCGATGAGCTGGGCAGGTGGTCTCTGTACCGCGCGGTGATCGCGGAGTTCGTGGCCACGCTGCTCTTCCTCTACATCACGGTGTCCACCGTCATCGGGTACAAG

**HvPIP2;7**  GCACCTCTGCTGGACACGAGCGAGCTGACCCGGTGGTCACTGTACCGCGCGGTCATCGCCGAGTTCGTGGCCACGCTCATCTTCCTCTACGTCAGCCTCGCCACCGTCATCGGCTACAAG

**HvPIP2;10**  GTCCGGGCGCTGGACACGAGCGAGCTGAGCACGTGGTCCCTGTACCGCGCCCTCATCGGCGAGTTCACGGCCTCCCTCATCCTCCTCTACGTCAGCATCGCCACCGTCATCGGATACCGG

**HvPIP2;8**  GCGCCGCTGGTCGACGCCGGCGAGCTGGGCAAGTGGTCTCTGTACCGTGCCGTCATCGCTGAGTTCACTGCCACGCTGCTCTTCGTCTACGTCGCCGTGGCCACCGTGGTCGGACACAAG

**HvPIP2;9**  GTCCGGCTGTTCGACGCCGGTGGGCTGGGCCAGTGGTCCCTGTACCGCGCCATCATCGCCGAGTTCACCGCCAGCCTGCTCTTCGTCTACGTCTCCATCGCCACCGTGATCGGCCACAAG

**HvPIP2;7a**  GCACCTCTGCTGGACACGAGCGAGCTGACCCGGTGGTCACTGTACCGCGCGGTCATCGCCGAGTTCGTGGCCACGCTCATCTTCCTCTACGTCAGCCTCGCCACCGTCATCGGCTACAAG

**HvPIP2;11**  ACGCCGCTGGTGGACGCCGGCGAGCTGGGCAAGTGGTCTTTGTACCGGGCCACCATTGCCGAGTTCACCGCCACCCTCCTCTTCGTCTACGTCGCCGTGGCCACCGTAATCGGCCACAAG

**HvPIP2;12**  GCGCCGCTCATCAACGCCGGCGAGCTGGGCAAGTGGTCACTCTGGCGCGCCGTCATCGCCGAGTTCACCGCCACGCTCCTCTTCGTCTACGTCACCGTCGCCACCGTCATCGGCCACAAG

**HvPIP2;2a**  GCGCCGCTGTTCGACATGGGGGAGCTCCGGATGTGGTCCTTCTACCGGGCGCTCATCGCCGAGTTCGTCGCCACGCTGCTCTTCCTCTACATCACCGTCGCCACCGTCATCGGCTACAAG

**OsPIP1;1**  GCGCCGCTGTTCGAGCCAGGGGAGCTCAAGTCGTGGTCTTTCTACCGGGCCGGGATCGCCGAGTTCGTCGCCACCTTCCTCTTCCTCTACATCACCATCCTCACCGTCAT-GGG---GGT

**OsPIP1;2**  GCGCCGCTGTTCGAGCCAGGGGAGCTCAAGTCGTGGTCCTTCTACCGCGCAGGGATCGCCGAGTTCATGGCCACCTTCCTCTTCCTTTACATCACCGTCCTCACCGTCAT-GGG---GGT

**OsPIP1;3**  GCGCCGGTGTTCGAGGTGGAGGAGCTGACGTCGTGGTCGTTCTACCGGGCGGGGATCGCGGAGTTCGTGGCGACGTTCCTGTTCCTGTACATCAGCATCCTGACGGTGAT-GGG---GGT

**OsPIP2;1**  GCGCCGCTGATCGACGCGGCGGAGCTGGGGTCGTGGTCGCTGTACCGCGCCGTCATCGCCGAGTTCATCGCCACGCTGCTGTTCCTGTACATCACCGTGGCCACGGTGATCGGGTACAAG

**OsPIP2;2**  GCGCCGCTGATCGACGTGGAGGAGCTGACCAAGTGGTCGCTGTACCGCGCGGTGATCGCGGAGTTCATCGCGACGCTCCTCTTCCTGTACATCACGGTGGCCACCGTGATCGGGTACAAG

**OsPIP2;3**  GCGCCGCTGATCGACGCGGAGGAGCTGACCAAGTGGTCCCTGTACCGCGCGGTGATCGCCGAGTTCGTGGCCACGCTGCTCTTCCTCTACATCACGGTGGCCACGGTCATCGGGTACAAG

**OsPIP2;4**  GCGCCGCTCGTCGACGTCGACGAGCTGGGCAAGTGGTCGCTGTACCGCGCGCTGATCGCCGAGTTCGTCGCCACGCTGCTCTTCCTCTACGTCACGGTGGCCACGGTGATCGGGTACAAG

**OsPIP2;5**  GCGCCTCTCGTCGACATCGACGAGCTCGGGAGGTGGTCGCTGTACCGCGCCGTCATCGCCGAGTTCGTGGCCACGCTGCTGTTCCTGTACGTCACGGTGGCCACGGTGATCGGGTACAAG

**OsPIP2;6**  GCGCCGCTGTTCGACGTCGGCGAGCTCCGGCTGTGGTCCTTCTACCGGGCGCTCATTGCGGAGTTCATCGCCACGCTCCTGTTCCTATACATCACCGTCGCCACCGTCATTGGGTACAAG

**OsPIP2;7**  GCGCCGCTGCTGGACACGAGCGAGCTGGGTAAGTGGTCGCTGTACCGTGCCCTCATCGCGGAGTTCATGGCGACGCTCATCTTCCTCTACGTGAGCATCGCCACCGTGATCGGGTACAAG

**OsPIP2;8**  GCGCCGCTAGTGGACACCGGCGAGCTAGGTAAGTGGTCACTGTATCGCGCCGCCATTGCGGAGTTCACAGCCACGCTGCTGTTGGTGTGCATCAGTGTGAGCACCGTGATCGGTGAGAAG

250 260 270 280 290 300 310 320 330 340 350 360

....|....|....|....|....|....|....|....|....|....|....|....|....|....|....|....|....|....|....|....|....|....|....|....|

**HvPIP1;1**  CAGCGG---CGC--------------------CGCCTCCAAGTGCGCCACCGTCGGCATCCAGGGCATCGCCTGGTCCTTCGGCGGCATGATCTTCGCCCTCGTCTACTGCACCGCCGGC

**HvPIP1;2**  GGGCAACCCCTC--------------------CGGCTCCAAGTGCGGCACCGTGGGCATCCAGGGCATCGCCTGGAGCTTCGGGGGCATGATCTTCGTGCTCGTCTACTGCACCGCCGGC

**HvPIP1;3**  GGGCAACCCGTC--------------------GGGGTCCAAGTGCGGCACGGTCGGCATCCAGGGCATCGCGTGGAGCTTCGGCGGCATGATCTTCGTGCTCGTCTACTGCACCGCCGGC

**HvPIP1;4**  GGGCAACCCGTC--------------------GGGGTCCAAGTGCGGCACCGTGGGCATCCAGGGCATCGCGTGGAGCTTCGGCGGCATGATCTTCGTGCTCGTCTACTGCACCGCCGGC

**HvPIP1;5**  CTCCAA---GGC--------------------CCCCTCCAAGTGCGCCACCGTCGGCGTCCAGGGCATCGCCTGGTCCTTCGGCGGCATGATCTTCGCGCTCGTCTACTGCACCGCCGGA

**HvPIP2;1**  CACCAGTCCGACCCCACCG---TCAACACCACCGACGCGGCCTGCAGCGGCGTCGGCATCCTCGGCATAGCATGGGCCTTCGGCGGCATGATCTTCGTCCTCGTCTACTGCACCGCCGGC

**HvPIP2;2**  GTGCAGTCGGCGGCC------------------GACCCG---TGCGCCGGCGTCGGGGTGCTCGGCATCGCCTGGGCCTTCGGCGGCATGATCTTCGTCCTCGTCTACTGCACCGCCGGC

**HvPIP2;3**  CACCAGGCGGACCCCGCCGGCCCCAATGCTGCCGACGCGGCGTGCAGCGGCGTCGGCATCCTCGGCATCGCCTGGGCGTTCGGCGGCATGATCTTCGTCCTCGTCTACTGCACCGCCGGT

**HvPIP2;4**  CACCAGGCGGACCCCGCCGGCCCCAACGCCGCCGACGCGGCCTGCAGCGGCGTGGGAATCCTGGGCATCGCCTGGGCGTTCGGCGGCATGATCTTCGTCCTCGTCTACTGCACCGCCGGT

**HvPIP2;5**  CACCAGACGGACCCCGCGGTGAACAGCGCCGCCGACGCGGCGTGCGGCGGCGTGGGCGTGCTCGGCATCGCGTGGGCCTTCGGCGGCATGATCTTCGTCCTCGTCTACTGCACCGCCGGC

**HvPIP2;6**  CACCAGACGGAC---GCGTCGGCCTCGGGCCCCGACGCGGCGTGCGGCGGGGTGGGCGTCCTCGGCATCGCGTGGGCGTTCGGGGGCATGATCTTCGTCCTCGTCTACTGCACCGCCGGC

**HvPIP2;7**  AGCCAGTCCTCCGCC-----------------CAGCCC----TGCACCGGCGTGGGGTACCTCGGCGTCGCCTGGGCCTTCGGCGCCACCATCTTCGTCCTCGTCTACTGCACCGGCGGC

**HvPIP2;10**  AACCAGTCCTCCGCCG---------------CCGACGAGCGGTGCACCGGCGTCGGCTACCTCGGCGTCGCCTGGTCCTTCGGCGCCACCGTCTCCGTCCTCGTCTACTCCACCAGCGGC

**HvPIP2;8**  CGCCAGACCGAC------------------G---CCCAGGCGTGCAGCGGCGCCGGCGTGCTGGGCATCGCGTGGGCCTTCGGCGGCACGATCGCCGTCCTCGTCTACTGCACCGCCGGC

**HvPIP2;9**  CGCCAGACGGAC------------------G---CGGACGCGTGCAGCGGCGCCGGCGTGCTGGGCATCGCGTGGGCCTTCGGCGGCATGATCGCCGTCCTCGTCTACTGCACCGCCGGC

**HvPIP2;7a**  AGCCAGTCCTCCGCC-----------------CAGCCC----TGCACCGGCGTGGGGTACCTCGGCGTCGCCTGGGCCTTCGGCGCCACCATCTTCGTCCTCGTCTACTGCACCGGCGGC

**HvPIP2;11**  CGCCAAACCGAC------------------G---CCCAAGCGTGCAGCGGCACCGGCGTGCTGGGCATCGCCTGGGCCTTCGGCGGCATGATCGCTGTCCTCGTCTACTGCACCGCCGGC

**HvPIP2;12**  CGCCAGACGGAC------------------GGCACCGTCGGCTGCGGCGGCGCGGGCATCCTCGGCATCGCGTGGGCCTTCGGCGGCATGATCTTCGTCCTCGTCTACTGCACCGCCGGC

**HvPIP2;2a**  GTGCAGTCGGCGGCC------------------GACCCG---TGCGCCGGCGTCGGGGTGCTCGGCATCGCCTGGGCCTTCGGCGGCATGATCTTCGTCCTAGTCTACTGCACCGCCGGC

**OsPIP1;1**  CTCCAA---GTC--------------------CTCCTCCAAGTGCGCCACCGTCGGCATCCAGGGCATCGCCTGGTCCTTCGGAGGCATGATCTTCGCGCTCGTCTACTGCACCGCCGGC

**OsPIP1;2**  CAACAA---CTC--------------------CACCTCCAAGTGCGCCACCGTCGGCATCCAGGGCATCGCCTGGTCCTTCGGCGGCATGATCTTCGCCCTCGTCTACTGCACCGCCGGC

**OsPIP1;3**  GAACAA---GTC--------------------GGCGTCCAAGTGCGCCACCGTGGGGATCCAGGGCATCGCGTGGTCGTTCGGCGGCATGATCTTCGCGCTCGTCTACTGCACCGCCGGC

**OsPIP2;1**  CACCAGACGGAC---GCGTCGGCCTCCGGCGCCGACGCGGCGTGCGGCGGCGTGGGCGTGCTCGGCATCGCGTGGGCGTTCGGCGGCATGATCTTCATCCTGGTCTACTGCACCGCCGGC

**OsPIP2;2**  CACCAGTCGGACGCCACCG---TCAACACCACCGACGCCGCCTGCAGCGGCGTCGGCATCCTCGGCATCGCCTGGGCCTTCGGCGGCATGATCTTCATCCTCGTCTACTGCACCGCCGGC

**OsPIP2;3**  CACCAGTCGGACCCGGGCG---CGAACGCCGCCGACGCCGCGTGCAGCGGCGTCGGCATCCTCGGCATCGCGTGGGCGTTCGGCGGCATGATCTTCATCCTCGTCTACTGCACCGCCGGC

**OsPIP2;4**  CACCAGACGGAC---GCCGCCGTGAACGGCGCCGACGCGGCGTGCGGCGGTGTGGGCGTCCTCGGCATCGCGTGGGCGTTCGGCGGCATGATCTTCATCCTCGTCTACTGCACCGCCGGC

**OsPIP2;5**  CACCAGACGGAC---GCGTCGGCGTCCGGCGACGACGCGGCGTGCGGCGGCGTGGGCGTCCTCGGCATCGCGTGGGCGTTCGGCGGCATGATCTTCATCCTGGTGTACTGCACCGCCGGC

**OsPIP2;6**  GTGCAGTCGTCCGCC------------------GACCAG---TGCGGCGGCGTCGGCACCCTCGGCATCGCCTGGGCCTTCGGTGGCATGATCTTCATCCTCGTCTACTGCACCGCCGGC

**OsPIP2;7**  AACCAGAGGGCCACCG---------------TCGACGCG---TGCACCGGCGTCGGCTACCTCGGCGTGGCGTGGTCGTTCGGTGCCACCATATTCGTCCTCGTCTACTGCACCGGCGGC

**OsPIP2;8**  CGGCAGTCGGGC------------------G------------AGGGCGGCGCCGGCGTGCTTGGCATTGCCTGGGCCTTTGGTGGCCTCATCTTCGTGCTGGTGTACTGCACCGCTGGC

370 380 390 400 410 420 430 440 450 460 470 480

....|....|....|....|....|....|....|....|....|....|....|....|....|....|....|....|....|....|....|....|....|....|....|....|

**HvPIP1;1**  ATCTCTGG----------------------------------------------------------------------------------------------------------------

**HvPIP1;2**  ATCTCCGG----------------------------------------------------------------------------------------------------------------

**HvPIP1;3**  ATCTCCGG----------------------------------------------------------------------------------------------------------------

**HvPIP1;4**  ATCTCCGG----------------------------------------------------------------------------------------------------------------

**HvPIP1;5**  ATCTCAGG----------------------------------------------------------------------------------------------------------------

**HvPIP2;1**  GTCTCAGG----------------------------------------------------------------------------------------------------------------

**HvPIP2;2**  ATCTCGGG----------------------------------------------------------------------------------------------------------------

**HvPIP2;3**  GTCTCGGG----------------------------------------------------------------------------------------------------------------

**HvPIP2;4**  GTCTCGGG----------------------------------------------------------------------------------------------------------------

**HvPIP2;5**  ATCTCCGG----------------------------------------------------------------------------------------------------------------

**HvPIP2;6**  ATCTCCGG----------------------------------------------------------------------------------------------------------------

**HvPIP2;7**  GTCTCAGG----------------------------------------------------------------------------------------------------------------

**HvPIP2;10**  GTCTCAGG----------------------------------------------------------------------------------------------------------------

**HvPIP2;8**  ATCTCCGG----------------------------------------------------------------------------------------------------------------

**HvPIP2;9**  ATCTCCGG----------------------------------------------------------------------------------------------------------------

**HvPIP2;7a**  GTCTCAGGCACGCATGCACGCACATATATATGCATACGATCGACCATCTCTCTACAACAAGATAACCCACACATGCATACAATATAGTAGGAGTATAAACTGTCGAGCGTGTGCTGCTGC

**HvPIP2;11**  ATCTCTGG----------------------------------------------------------------------------------------------------------------

**HvPIP2;12**  GTCTCCGG----------------------------------------------------------------------------------------------------------------

**HvPIP2;2a**  ATCTCGGG----------------------------------------------------------------------------------------------------------------

**OsPIP1;1**  ATCTCCGG----------------------------------------------------------------------------------------------------------------

**OsPIP1;2**  ATCTCCGG----------------------------------------------------------------------------------------------------------------

**OsPIP1;3**  ATCTCCGG----------------------------------------------------------------------------------------------------------------

**OsPIP2;1**  ATCTCCGG----------------------------------------------------------------------------------------------------------------

**OsPIP2;2**  ATCTCAGG----------------------------------------------------------------------------------------------------------------

**OsPIP2;3**  GTGTCCGG----------------------------------------------------------------------------------------------------------------

**OsPIP2;4**  GTCTCCGG----------------------------------------------------------------------------------------------------------------

**OsPIP2;5**  ATCTCCGG----------------------------------------------------------------------------------------------------------------

**OsPIP2;6**  ATCTCCGG----------------------------------------------------------------------------------------------------------------

**OsPIP2;7**  GTCTCCGG----------------------------------------------------------------------------------------------------------------

**OsPIP2;8**  ATCTCCGG----------------------------------------------------------------------------------------------------------------

490 500 510 520 530 540 550 560 570 580 590 600

....|....|....|....|....|....|....|....|....|....|....|....|....|....|....|....|....|....|....|....|....|....|....|....|

**HvPIP1;1**  ---CGGGCACATCAACCCGGCGGTGACCTTCGGGCTGTTCCTGGCGAGGAAGCTGTCGCTGACGAGGGCGGTGTTCTACATCATCATGCAGTGCCTGGGCGCCATCTGCGGCGCCGGCGT

**HvPIP1;2**  ---CGGCCACATCAACCCCGCCGTCACCTTCGGGCTGTTCCTGGCCAGGAAGCTGTCGCTCACCCGGGCCGTGTTCTACATGGTGATGCAGTGCCTGGGAGCCATCTGCGGCGCCGGGGT

**HvPIP1;3**  ---CGGCCACATCAACCCGGCGGTCACCTTCGGGCTGTTCTTGGCCAGGAAGCTGTCGCTCACCAGGGCCGTCTTCTACATCGTGATGCAGTGCCTCGGGGCCATATGCGGCGCCGGCGT

**HvPIP1;4**  ---CGGCCACATCAACCCCGCGGTCACCTTCGGGCTGTTCTTGGCCAGGAAGCTGTCGCTCACCAGGGCCGTCTTCTACATCGTGATGCAGTGCCTCGGGGCCATATGCGGCGCCGGCGT

**HvPIP1;5**  ---AGGACACATCAACCCAGCAGTGACTTTTGGGCTGTTCTTGGCCAGGAAGCTGTCCCTGACCAGGGCCATCTTCTACATAATCATGCAATGCCTAGGGGCCATCTGTGGGGCTGGAGT

**HvPIP2;1**  ---TGGACACATCAACCCGGCGGTGACCTTCGGGTTGTTCCTGGCGAGGAAGGTGTCGCTCATCCGGGCGCTGCTCTACATCATCGCGCAGTGCCTCGGCGCCATCTGCGGCGTCGGCCT

**HvPIP2;2**  ---CGGCCACATCAACCCGGCCGTCACCTTCGGCCTCCTCCTCGCGCGCAAGGTGTCGCTGCTGCGCGCCGTCATGTACATCGTGGCGCAGTGCGCCGGCGGCATCGTGGGCGCCGGCAT

**HvPIP2;3**  ---TGGCCACATCAACCCGGCGGTGACGTTCGGGCTGTTCCTGGCGCGCAAGGTTTCGCTGGTGCGCGCGGTGCTCTACATCATCGCGCAGTGCCTGGGCGCCATCTGTGGCGTGGGGCT

**HvPIP2;4**  ---TGGCCACATCAACCCGGCGGTGACCTTCGGGCTGTTCCTGGCGCGCAAGGTGTCGCTGGTGCGCGCGGTGCTCTACATCATCGCGCAGTGCCTGGGCGCCATCTGCGGCGTGGGGCT

**HvPIP2;5**  ---GGGCCACATCAACCCGGCCGTGACCTTCGGCCTCTTCCTGGCGCGCAAGGTCTCCCTGGTCCGCGCGCTGCTCTACATGGTGGCGCAGTGCCTCGGCGCCATGTGCGGGGTCGGCCT

**HvPIP2;6**  ---CGGCCACATCAACCCGGCCGTGACGTTCGGCCTCTTCCTGGCGCGCAGGGTGTCCCTGGTGCGCGCGCTGCTCTACATGGCCGCGCAGTGCCTCGGCGCCGTCTGTGGCGTCGGGCT

**HvPIP2;7**  ---TGGTCACATCAACCCGGCGGTGACGTTCGGGCTGTTCGTGGGGAGGAAGCTGTCGCTGGTGCGCACGGTGCTGTACATCGTGGCGCAGTGCCTCGGCGCCATCTGCGGCGCGGGCAT

**HvPIP2;10**  ---TGGGCACATAAACCCGGCAGTGACGTTCGCGCTGTTCATCGCCGGGAAGGTGACGCTGGTGCGCTCGGTGCTGTACGTGGTGGCGCAGTGCCTCGGCGCCGTCGTCGGCGTGGGCAT

**HvPIP2;8**  ---CGGCCACATCAACCCCGCGGTCACCTTCGGCCTCCTCCTGGCGCGCAAGGTCTCCCTTCCCAGGGCCTTCCTGTACATGGTGGCGCAGTGCGTGGGCGCCATCTGCGGCGCGGCGCT

**HvPIP2;9**  ---CGGCCACGTCAACCCCGCCGTGACGTTCGGGCTGCTGCTGGCGAGGAAGCTGTCGCTCCCCCGCGCCTTGCTCTACACGTCGGCGCAGTGCCTCGGCGCCATCTGCGGCGCGGCGAT

**HvPIP2;7a**  AGGTGGTCACATCAACCCGGCGGTGACGTTCGGGCTGTTCGTGGGGAGGAAGCTGTCGCTGGTGCGCACGGTGCTGTACATCGTGGCGCAGTGCCTCGGCGCCATCTGCGGCGCGGGCAT

**HvPIP2;11**  ---CGGCCACATCAACCCCGCGGTGACGTTCGGGCTGCTGCTGGCGAGGAAGGTCTCGCTTCCCAGAGCCTTCTTCTACATGGCGGCGCAGTGCCTCGGCGCCATCTGCGGCGCCGGCCT

**HvPIP2;12**  ---CGGCCACATCAACCCCGCCGTCACCTTCGGCCTCCTGCTGGCCCGCAAGGTGTCCCTCGTCCGCGCGCTGCTCTACATGGTCGCCCAGTGCCTCGGCGCCATGTGCGGCGCCGGCCT

**HvPIP2;2a**  ---CGGCCACATCAACCCGGCCGTCACCTTCGGCCTCCTCCTCGCGCGCAAGGTGTCGCTGCTGCGCGCCGTCATGTACATCGTGGCGCAGTGCGCCGGCGGCATCGTGGGCGCCGGCAT

**OsPIP1;1**  ---AGGACACATCAACCCAGCAGTTACTTTTGGGCTGTTCTTGGCCAGGAAGCTGTCCCTGACCCGGGCCATCTTCTACATAGTGATGCAATGCCTAGGGGCCATCTGCGGAGCTGGAGT

**OsPIP1;2**  ---CGGCCACATCAACCCGGCCGTCACCTTCGGCCTCTTCCTCGCCAGGAAGCTGTCCCTCACCAGGGCCCTCTTCTACATGGTGATGCAGTGCCTCGGCGCCATCTGTGGCGCCGGCGT

**OsPIP1;3**  ---CGGGCACATCAACCCGGCGGTGACGTTCGGGCTGTTCCTGGCGCGGAAGCTGTCGCTGACGCGGGCGGTGTTCTACATGGCGATGCAGTGCCTGGGCGCCATCTGCGGCGCCGGCGT

**OsPIP2;1**  ---CGGGCACATCAACCCGGCGGTGACGTTCGGGCTCTTCCTGGCGCGCAAGGTGTCCCTGGTCCGCGCCATCCTCTACATCGTGGCGCAGTGCCTCGGCGCCATCTGCGGCGTCGGCCT

**OsPIP2;2**  ---TGGGCACATCAACCCGGCGGTGACGTTCGGGCTGTTCCTGGCGAGGAAGGTGTCGCTGATCAGGGCGGTGCTGTACATCATCGCGCAGTGCCTGGGCGCCATCTGCGGCGTGGGGCT

**OsPIP2;3**  ---CGGCCACATCAACCCGGCGGTGACGTTCGGGCTCTTCCTGGCGCGCAAGGTGTCGCTGGTGCGCGCGGTGCTCTACATCGTAGCGCAGAGCCTCGGCGCCATCTGCGGCGTCGGGCT

**OsPIP2;4**  ---CGGGCACATCAACCCGGCGGTGACACTCGGCCTCTTCCTGGCGCGGAAGGTGTCCCTGGTGCGCGCCCTCCTGTACATGGCGGCGCAGTGCCTCGGCGCCATCTGCGGCGTCGCGCT

**OsPIP2;5**  ---CGGGCACATCAACCCGGCGGTGACGTTCGGCCTCTTCCTGGCGCGCAAGGTGTCCCTGGTCCGCGCCATCCTCTACATCGTGGCGCAGTGCCTCGGCGCCGTCTGCGGCGTCGCGCT

**OsPIP2;6**  ---AGGGCACATTAACCCCGCGGTGACGTTCGGGCTGCTGCTGGCGAGGAAGGTGTCGGTGATTCGCGCGGTGATGTACATCGTGGCGCAGTGCCTGGGCGGCATCGTGGGCGTGGGCAT

**OsPIP2;7**  ---CGGGCACATCAACCCGGCGGTGACGCTGGGCCTCTTCTTCGGGCGGAAGCTGTCGCTCGTCCGCACCGTGCTGTACGTCGTGGCGCAGTGCCTCGGCGCCATCGCCGGCGCCGGCAT

**OsPIP2;8**  ---GGGGCACATGAACCCAGCCGTGACCTTCGCCATGGTGCTCGCGCGAAGGGTGTCGCTGCCGCGAGCGGCGCTGTACACGATGGCGCAGTGCGTGGGCGCCGTGTGCGGCGCAGGGCT

610 620 630 640 650 660 670 680 690 700 710 720

....|....|....|....|....|....|....|....|....|....|....|....|....|....|....|....|....|....|....|....|....|....|....|....|

**HvPIP1;1**  GGTCAAGGGGTTCCAGCAGGGC---CTGTACATGGGCAACGGCGGCGGCGCCAACGTGGTGGCGTCCGGCTACACCAAGGGCTCCGGGCTCGGCGCCGAGATCATCGGCACCTTCGTCCT

**HvPIP1;2**  GGTGAAGGGGTTCCAGACCACG---CTGTACATGGGCAACGGCGGCGGCGCCAACTCGGTGGCGCCGGGGTACACCAAAGGCGACGGCCTGGGGGCGGAGATCGTGGGGACCTTTGTGCT

**HvPIP1;3**  GGTGAAGGGGTTCCAGACCACG---CTGTACCAGGGCAACGGCGGCGGCGCCAACTCCGTCGCTGCCGGGTACACCAAGGGGGATGGGTTGGGCGCCGAGATCGTCGGCACGTTCGTGCT

**HvPIP1;4**  GGTGAAGGGGTTCCAGACCACG---CTGTACCAGGGCAACGGCGGCGGCGCCAACTCCGTCGCTGCCGGGTACACCAAGGGGGATGGGTTGGGCGCCGAGATCGTCGGCACGTTCGTGCT

**HvPIP1;5**  GGTGAAGGGCTTCCAGCAGGGT---CTGTACATGGGCAACGGTGGCGGCGCCAATGTAGTTGCCAGTGGCTACACCAAGGGCGATGGCCTTGGTGCCGAGATCATTGGCACCTTCGTCCT

**HvPIP2;1**  TGTCAAGGGTTTCCAGAGCTCC---TACTACGTGCGGTACGGCGGTGGCGCCAACGAGCTCAGCGCAGGCTACTCCAAGGGCACCGGTCTAGCGGCCGAGATCATCGGCACCTTCGTGCT

**HvPIP2;2**  CGTCAAGGGCATCATGAAGGAC---GCGTACCAGGCCAACGGCGGCGGCGCCAACATGGTCGCCTCGGGCTTCTCCCGCGGCACCGCGCTGGGGGCAGAGATCGTCGGCACCTTCGTCCT

**HvPIP2;3**  CGTCAAGGGGTTCCAGAGCGCC---TTCTACGTGCGCTACGGCGGCGGCGCCAACGAGCTCAGCGCGGGCTACTCCAAGGGCACCGGGCTCGCCGCCGAGATCATCGGCACCTTCGTGCT

**HvPIP2;4**  CGTCAAGGGGTTCCAGAGCGCC---TTCTACGTGCGCTACGGCGGGGGCGCCAACGAGCTCAGCGCCGGCTACTCCAAGGGCACCGGCCTCGCCGCCGAGATCATCGGCACCTTCGTGCT

**HvPIP2;5**  CGTCAAGGCCTTCCAGAGCGCC---TACTTCGTCAGGTACGGCGGCGGCGCCAACACGCTCGCCGCCGGATACTCCAAGGGCACCGGCCTCGCCGCGGAGATCATCGGCACCTTCGTGCT

**HvPIP2;6**  CGTCAGGGGGTTCCAGAGCGGG---CTCTACGCGCGCCACGGCGGCGGCGCGAACGAGGTCGGGGCCGGGTACTCGGTCGGCACGGGGCTCGCCGCGGAGATCGTCGGTACCTTCGTGCT

**HvPIP2;7**  GGTGAAGGGGATCGCGGGGGCC---AGCTACGAGGCCCTCGGCGGCGGCGCAAACACGGTGGCCGACGGCGTCTCGGTCGGCGCGGGGCTCGGGGCGGAGATCGCCGGCACGTTCGTGCT

**HvPIP2;10**  CGTGAAGGGGATCATGAAGCAC---CCGTACGACGACTTCGGCGGCGGCGCCAACGCGGTGGCCGGGGGGTACTCCCTCGGCGCGGCCCTCGGCGCGGAGATCTTCGGCACCTTCGTCCT

**HvPIP2;8**  GGTGAGAGCCGTGCACGGCGGCCACCACTACGCTCTCTACGGAGGCGGCGCCAACGAGCTCGCGCCGGGCTACTCCAGGATGGCGGGGCTCATCGCGGAGATCGCCGGCACCTTCGTGCT

**HvPIP2;9**  GGTCAGGACCGTGCACGGCGCGCAGCACTACGAGCTCTACGGCGGCGGCGCCAACGAGGTCGCGCCGGGGTACTCGAAGGCGGGGGGCCTGCTGGCCGAGGCGGCCGGCACCTTCCTCCT

**HvPIP2;7a**  GGTGAAGGGGATCGCGGGGGCC---AGCTACGAGGCCCTCGGCGGCGGCGCAAACACGGTGGCCGACGGCGTCTCGGTCGGCGCGGGGCTCGGGGCGGAGATCGCCGGCACGTTCGTGCT

**HvPIP2;11**  TCTTAGGGCCGTGCACGGCGCGCACCACTACGAGCTATACGGCGGCGGCGCCAACGAGGTAGCGCCGGGGTACTCCAAGGCGGGGGCATTGGTGGCCGAGGCCGCCGGGACGTTCGTTCT

**HvPIP2;12**  CGTCAGGGCCGTGCACGGCGCC---CAGTACGCGCGCCACGGTGGGGGCGCCAACGAGCTCGCCCCGGGATACTCCAAGGTCGCCGGGCTCGTCGCCGAGATCGTGGGCACCTTCGTGCT

**HvPIP2;2a**  CGTCAAGGGCATCATGAAGGAC---GCGTACCAGGCCAACGGCGGCGGCGCCAACATGGTCGCCTCGGGCTTCTCCCGAGGCACCGCGCTGGGGGCAGAGATCGTCGGCACCTTCGTCCT

**OsPIP1;1**  TGTGAAGGGCTTCCAGCAGGGT---CTGTACATGGGCAATGGCGGTGGTGCCAATGTAGTTGCCAGTGGCTACACCAAGGGTGACGGTCTTGGTGCTGAGATTGTTGGCACCTTCATCCT

**OsPIP1;2**  CGTCAAGGGCTTCCAGAAGGGC---CTGTACGAGACCACCGGCGGCGGCGCCAACGTCGTCGCGCCCGGCTACACCAAGGGCGACGGCCTCGGCGCCGAGATCGTCGGCACCTTCATCCT

**OsPIP1;3**  GGTGAAGGGGTTCCAGCGGGGG---TTGTACATGGGCTCCGGCGGCGGCGCCAACGCCGTGAACCCGGGGTACACCAAGGGGGACGGGCTCGGGGCGGAGATCGTGGGCACCTTCGTCCT

**OsPIP2;1**  CGTCAAGGCGTTCCAGAGCGCC---TACTTCAACAGGTACGGCGGCGGCGCCAACACCCTCGCCGCCGGCTACTCCAAGGGCACCGGCCTCGCCGCCGAGATCATCGGCACCTTCGTGCT

**OsPIP2;2**  CGTCAAGGGGTTCCAGAGCTCG---TACTACGCGCGGTACGGCGGCGGAGCCAACGAGCTCAGCGACGGCTACTCCAAGGGCACCGGCCTCGGCGCGGAGATCATCGGCACCTTCGTGCT

**OsPIP2;3**  CGTCAAGGGGTTCCAGAGCGCC---TTCTACGTGCGCTATGGCGGCGGCGCCAACGAGCTCAGCGACGGCTACTCCAAGGGCACCGGCCTCGCCGCCGAGATCATCGGCACCTTCGTGCT

**OsPIP2;4**  CGTCAAGGGGTTCCAGAGCTCG---CTCTACGACAGGTACGGCGGCGGCGCCAACGAGCTCGCCGCCGGCTACTCCACCGGCACCGGCCTCGCCGCCGAGATCATCGGCACCTTCGTGCT

**OsPIP2;5**  CGTCAAGGGGTTCCAGAGCTCG---TTCTACGACAGGTACGGCGGCGGCGCCAACGAGCTCGCCGCCGGCTACTCCAAGGGCACCGGCCTCGCCGCCGAGATCATCGGCACCTTCGTGCT

**OsPIP2;6**  CGTGAAGGGCATCATGAAGCAC---CAGTACAACGCCAACGGCGGCGGCGCCAACATGGTGGCCAGCGGCTACTCCACCGGCACCGCCCTCGGCGCCGAAATCATCGGCACCTTCGTCCT

**OsPIP2;7**  CGTCAAGGGGATCATGAAGCGC---CCCTACGACGCGCTCGGCGGCGGCGCCAACACCGTCAGCGACGGCTACTCCGCCGCCGGCGCCCTCGGCGCCGAGATCGTCGGCACGTTCATCCT

**OsPIP2;8**  CGCCAGGGCGATGCACGGTGGCGGGCAGTACGCGCGGCACGGCGGAGGTGCCAACGAGCTGGCGGCGGGCTACTCGGCGGGGGCAGGGGTCGTGGCGGAGATGGTGGGCACGTTCGTGCT

730 740 750 760 770 780 790 800 810 820 830 840

....|....|....|....|....|....|....|....|....|....|....|....|....|....|....|....|....|....|....|....|....|....|....|....|

**HvPIP1;1**  CGTCTACACCGTCTTCTCCGCCACCGACGCCAAGAGGAACGCCAGGGACTCCCACGTTCCCATCCTCGCCCCGCTGCCCATCGGGTTCGCCGTGTTCCTGGTCCACCTGGCCACCATCCC

**HvPIP1;2**  CGTGTACACCGTCTTCTCCGCCACCGACGCCAAGCGCAGCGCCAGAGACTCCCACGTCCCCATTTTGGCGCCGCTGCCGATCGGGTTCGCGGTGTTCCTGGTGCACCTGGCGACAATCCC

**HvPIP1;3**  GGTGTACACCGTCTTCTCCGCCACCGACGCCAAGCGCAGCGCCAGAGACTCCCACGTCCCCATTTTGGCGCCGCTTCCGATCGGGTTCGCGGTGTTCCTGGTGCACCTGGCGACGATCCC

**HvPIP1;4**  GGTGTACACCGTCTTCTCCGCAACCGACGCCAAGCGCAGCGCCAGAGACTCCCACGTCCCCATTTTGGCGCCGCTTCCGATCGGGTTCGCGGTGTTCCTGGTGCACCTGGCGACGATCCC

**HvPIP1;5**  GGTCTACACCGTCTTCTCCGCCACTGATGCCAAGAGGAATGCCAGGGACTCGCATGTTCCTATCCTTGCCCCGCTGCCGATTGGGTTCGCGGTGTTCCTGGTCCACCTGGCCACCATTCC

**HvPIP2;1**  CGTCTACACGGTCTTCTCCGCCACCGACCCCAAGCGCAACGCCCGTGATTCCCACATCCCGGTGCTGGCTCCTCTCCCAATCGGATTCGCTGTGTTCATGGTCCACTTGGCCACCATCCC

**HvPIP2;2**  CGTCTACACCGTCTTCTCCGCCACCGACCCCAAGCGCAGCGCCCGCGACTCCCACGTCCCCGTGCTGGCGCCGCTCCCCATCGGCTTCGCCGTCTTCATGGTGCACCTCGCCACCATCCC

**HvPIP2;3**  CGTCTACACCGTCTTCTCCGCCACCGACCCCAAGCGCAGCGCCCGTGACTCCCACGTCCCAGTGCTGGCTCCTTTGCCAATCGGCTTCGCGGTGTTCATGGTGCACCTGGCCACTATCCC

**HvPIP2;4**  CGTCTACACCGTCTTCTCCGCCACCGACCCCAAGCGCAGCGCCCGTGACTCCCACGTCCCAGTCCTGGCTCCCCTGCCAATCGGCTTCGCGGTGTTCATGGTGCACTTGGCCACTATCCC

**HvPIP2;5**  CGTCTACACCGTCTTCTCCGCCACCGACCCCAAGCGCAGCGCACGCGACTCTCACGTCCCGGTGTTGGCGCCCCTCCCGATCGGCTTCGCCGTGTTCATGGTCCACCTGGCCACCATCCC

**HvPIP2;6**  CGTCTACACCGTCTTCTCCGCCACCGACTCCAAGCGCAACGCTCGTGACTCCCACGTCCCGATGTTGGCACCGCTGCCGATCGGGTTCGCGGTGTTCATGGTGCACCTGGCCACCATCCC

**HvPIP2;7**  GGTGTACACCGTCCTCTCCGCCACCGACCCCAAGCGCACCGCGCGCGACTCCTTCATCCCCGTGCTGGTGCCGCTGCCCATCGGCTTCGCCGTGTTCATCGTGCACCTGGCCACCATACC

**HvPIP2;10**  CGCCTACACCGTCTTCTCCGCCACCGACCCCAAGCGCACCGCCCGCGACGCCTTCGTCCCCCTGGTGGCCGCGCTCCCGATCGGGTTGTCGGTGTTCGTGGTGCACCTGGCGACCATCCC

**HvPIP2;8**  CGTGTACACGGTGTTCTCGGCGACGGACCCCAAGCGCATCGCCCGGGACCCGCACGTCCCGGTGCTGGCGCCGCTGCTCATCGGGTTCTCGGTGCTCATGGCGCACCTCGCCACCATCCC

**HvPIP2;9**  CGTGTACACCGTGTTCTCGGCGACCGACCCGAAGCGCATGGCGCGGGACACCCATGTGCCGGTGCTGGCGCCGCTGCTCATCGGCTTCGCGGTCGTGGTGGCGCACCTGGCCACCATCCC

**HvPIP2;7a**  GGTGTACACCGTCCTCTCCGCCACCGACCCCAAGCGCACCGCGCGCGACTCCTTCATCCCCGTGCTGGTGCCGCTGCCCATCGGCTTCGCCGTGTTCATCGTGCACCTGGCCACCATACC

**HvPIP2;11**  CGTCTACACCGTGTTCTCGGCGACCGACCCGAAGCGCATGGCGCGGGACTCCCACGTGCCGGTGTTGGCGCCGCTGCTCATCGGTTTCGCCGTGCTGATGGCGCACCTGGCCACCATCCC

**HvPIP2;12**  CGTCTACACCGTGTTCGCCGCCACCGACCCCAAGCGCAAGGCGAGGGACTCCCACGTGCCCGTGCTGGCGCCGCTGCCCATCGGGTTCGCGGTGCTCATGGTTCACCTGGCCACCATCCC

**HvPIP2;2a**  CGTCTACACCGTCTTCTCCGCCACCGACCCCAAGCGCAGCGCCCGCGACTCCCACGTCCCCGTGCTGGCGCCGCTCCCCATCGGCTTCGCCGTCTTCATGGTGCACCTCGCCACCATCCC

**OsPIP1;1**  GGTCTACACCGTCTTCTCAGCCACTGATGCCAAGAGGAATGCCAGGGACTCACATGTTCCTATCCTTGCCCCACTGCCAATTGGTTTTGCGGTGTTCCTGGTCCACCTGGCCACCATCCC

**OsPIP1;2**  CGTCTACACCGTCTTCTCCGCCACCGACGCCAAGAGGAACGCCAGGGACTCCCACGTTCCGATCCTTGCCCCACTGCCAATCGGGTTTGCGGTGTTCTTGGTTCACCTGGCCACCATCCC

**OsPIP1;3**  CGTCTACACCGTCTTCTCCGCCACCGACGCCAAGCGCAACGCCAGGGACTCCCACGTCCCCATCCTGGCGCCGCTCCCCATCGGCTTCGCCGTCTTCCTCGTCCACCTCGCCACCATCCC

**OsPIP2;1**  CGTCTACACCGTCTTCTCCGCCACCGACCCCAAGCGCAACGCCCGCGACTCACATGTCCCGGTCTTGGCGCCGCTGCCAATCGGCTTCGCCGTGTTCATGGTCCACCTGGCGACGATCCC

**OsPIP2;2**  CGTCTACACCGTCTTCTCCGCCACCGACCCCAAGCGCAACGCCCGCGACTCCCACATCCCGGTGTTGGCTCCTCTCCCAATTGGATTCGCGGTGTTCATGGTCCATCTGGCCACCATCCC

**OsPIP2;3**  CGTCTACACCGTCTTCTCCGCCACCGACCCCAAGCGCAACGCCCGCGACTCCCACGTCCCCGTGCTTGCTCCTCTTCCAATTGGGTTCGCGGTGTTCATGGTTCACTTGGCCACGATCCC

**OsPIP2;4**  CGTGTACACCGTCTTCTCCGCCACCGATCCCAAGCGCAACGCCCGCGACTCACATGTTCCCGTGTTGGCTCCGCTGCCAATCGGGTTCGCGGTGTTCATGGTGCACCTGGCGACGATCCC

**OsPIP2;5**  CGTGTACACCGTCTTCTCCGCCACCGACCCCAAGCGCAACGCCCGCGACTCCCATGTCCCGGTGTTGGCGCCGCTGCCGATCGGGTTCGCCGTGTTCATGGTGCACCTGGCGACCATCCC

**OsPIP2;6**  CGTCTACACCGTCTTCTCCGCCACCGACCCAAAGCGCAATGCCCGCGACTCCCACGTCCCGGTGCTCGCGCCGCTGCCCATCGGGTTCGCCGTGTTCATGGTGCACCTGGCCACCATCCC

**OsPIP2;7**  CGTCTACACCGTCTTCTCCGCCACCGACCCCAAGCGCACCGCCCGCGACTCCTTCATCCCCGTACTCGTGCCGCTGCCGATCGGGTTCGCGGTGTTCGTCGTGCACCTGGCGACGATTCC

**OsPIP2;8**  GGTGTACACGGTGTTCTCGGCGACGGACCCGAAGCGGAAGGCCAGGGACTCTCACGTGCCGGTGCTGGCGCCGCTTCCCATCGGGTTGGCGGTGCTGGTTGTGCACCTTGCCACCATCCC

850 860 870 880 890 900 910 920 930 940 950 960

....|....|....|....|....|....|....|....|....|....|....|....|....|....|....|....|....|....|....|....|....|....|....|....|

**HvPIP1;1**  CATCACCGGCACCGGCATCAACCCGGCGAGGAGCCTCGGCGCGGCCATCATCTACAAC------AGGGAGCACGCCTGGTCAGACCAC--------------------------------

**HvPIP1;2**  CATCACCGGCACCGGCATCAACCCGGCGCGCTCCCTCGGCGCCGCCATCATCTACAAC------AAGAAGCAGTCGTGGGACGACCAC--------------------------------

**HvPIP1;3**  CATCACGGGCACCGGCATCAACCCGGCGAGGTCCCTCGGCGCCGCCATCATCTACAAC------AAGAAGCAGGCGTGGGACGACCAC--------------------------------

**HvPIP1;4**  CATCACGGGCACCGGCATCAACCCGGCGAGGTCCCTCGGCGCCGCCATCATCTACAAC------AAGAAGCAGGCGTGGGACGACCAC--------------------------------

**HvPIP1;5**  CATCACCGGCACCGGCATCAACCCAGCTAGGAGCCTTGGGGCTGCCATCATCTACAAC------AGGGACCATGCCTGGAATGACCAT--------------------------------

**HvPIP2;1**  CATCACCGGCACCGGCATCAACCCCGCGAGAAGCTTGGGAGCTGCTGTCATCTACAAC------ACTGACAAGGCCTGGGATGACCAA--------------------------------

**HvPIP2;2**  CATCACCGGCACCGGCATCAACCCCGCCAGGAGCCTCGGCGCCGCGGTCATCTACAAC------AAGAAGGCCGCATGGGACAACCAC--------------------------------

**HvPIP2;3**  AATCACCGGCACCGGCATCAACCCGGCAAGGAGCTTCGGAGCTGCCGTGATCTACAAC------AACGAGAAGGCCTGGGATGACCAC--------------------------------

**HvPIP2;4**  GATCACCGGCACCGGCATCAACCCGGCGAGGAGCTTCGGAGCTGCCGTGATCTACAAC------AACGAGAAGGCCTGGGATGACCAC--------------------------------

**HvPIP2;5**  GATCACCGGCACCGGCATCAACCCGGCCAGGAGCCTGGGGGCCGCCGTCATCTACAAC------AAGGACAAGGCCTGGGATGATCAG--------------------------------

**HvPIP2;6**  GATCACCGGGACGGGGATCAACCCGGCGAGGAGCCTCGGTGCCGCCGTCATCTACAAC------GGCGACAAGGCCTGGAGCGATCAG--------------------------------

**HvPIP2;7**  CATCACCGGCACCGGCATCAACCCGGCCAGGAGCCTCGGCGCCGCCGTCATGTACAAC------CAGCACAAGGCATGGAAGGACCAC--------------------------------

**HvPIP2;10**  GATCACCGGCACGGGCATCAACCCGGCGAGGAGCCTGGGCGCCGCCGTCCTGTACAAC------CAGCACAAGACCTGGAAGCAACAC--------------------------------

**HvPIP2;8**  CGTCACCGGCACCGGGATCAACCCGGCGAGGAGCTTTGGTGCCGCCGTGGTGTACAAC------AACAAGAAAGCTTGGGGAGACCAG--------------------------------

**HvPIP2;9**  CGTCACCGGCACCGGGATCAACCCGGCGAGGAGCCTGGGTGCCGCCGTGGTGTACAAC------AACAGCAAGGCGTGGCGCGAGCAG--------------------------------

**HvPIP2;7a**  CATCACCGGCACCGGCATCAACCCGGCCAGGAGCCTCGGCGCCGCCGTCATGTACAAC------CAGCACAAGGCATGGAAGGACCACGTAAGTATATATACCACGAATTCGATCACGAT

**HvPIP2;11**  CATCACCGGCACCGGGATTAACCCGGCGAGAAGCCTTGGCGCCGCCGTGGTGTACAAT------GGCAAGAAGGCCTGGGCCGATCAG--------------------------------

**HvPIP2;12**  CATCACCGGCACCGGGATCAACCCGGCCAGGAGCCTCGGGGCCGCGGTGGTGTACAAC------AAGAAGAAGGCGTGGGACGAGCAG--------------------------------

**HvPIP2;2a**  CATCACCGGCACCGGCATCAACCCCGCCAGGAGCCTCGGCGCCGCGGTCATCTACAAC------AAGAAGGCCGCATGGGACAACCACGTAAGCTACCTTCCGTCGTCTCCGTTAACCTC

**OsPIP1;1**  CATCACCGGTACTGGCATCAACCCAGCCAGGAGCCTTGGCGCTGCCATCATCTACAAC------AAGGACCATGCCTGGAATGACCAT--------------------------------

**OsPIP1;2**  CATCACCGGCACCGGCATCAACCCAGCGAGGAGCCTTGGCGCTGCCATCATCTACAAC------AGGGGCCATGCCTGGGATGACCAT--------------------------------

**OsPIP1;3**  CATCACCGGCACCGGCATCAACCCCGCCCGCAGCCTCGGCGCCGCCATCGTCTATAAC------CGCGCCCACGCATGGCACGACCAC--------------------------------

**OsPIP2;1**  GATCACCGGCACCGGCATCAACCCGGCCAGGAGCATCGGAGCGGCCGTCATCTTCAAC------AACGAGAAGGCGTGGCACAACCAT--------------------------------

**OsPIP2;2**  CATCACCGGCACCGGCATTAACCCGGCGAGGAGCTTGGGAACCGCGGTTATCTACAAC------AAGGACAAGGCCTGGGATGACCAA--------------------------------

**OsPIP2;3**  GATCACCGGCACCGGCATCAACCCGGCAAGGAGCTTGGGAGCTGCGGTGATCTACAAC------CAGCACAAGGCATGGCATGACCAC--------------------------------

**OsPIP2;4**  GATCACCGGCACCGGCATCAACCCGGCCAGGAGCCTCGGCGTCGCCGTGGTCTACAAC------AACAACAAGGCCTGGAGTGATCAG--------------------------------

**OsPIP2;5**  CGTCACCGGCACCGGCATCAACCCGGCGAGGAGCCTCGGCGCCGCCGTCGTCTACAAC------AACAGCAAGGCATGGAGTGATCAG--------------------------------

**OsPIP2;6**  CATCACCGGCACGGGCATCAACCCTGCCCGGAGCATCGGCGCCGCCGTCATCTACAAC------CAGAAGAAGGCATGGGACGACCAC--------------------------------

**OsPIP2;7**  GATCACCGGCACGGGCATCAACCCGGCGAGGAGCCTCGGCGCCGCCGTGCTGTACAAC------CAGCACGCAGCTTGGAAAGACCAC--------------------------------

**OsPIP2;8**  CATCACCGGCACTGGCATCAACCCCGCCCGCAGCCTTGGCCCCGCCCTCGTCCTCGGCCTCGGCACCACCAAGGCCTGGTCCCACCTC--------------------------------

970 980 990 1000 1010 1020 1030 1040 1050 1060 1070 1080

....|....|....|....|....|....|....|....|....|....|....|....|....|....|....|....|....|....|....|....|....|....|....|....|

**HvPIP1;1**  ------------------------------------------------------------------------------------TGGATCTTCTGGGTCGGCCCCTTCATCGGCGCCGCG

**HvPIP1;2**  ------------------------------------------------------------------------------------TGGATCTTCTGGGTGGGCCCGTTCACCGGCGCGGCG

**HvPIP1;3**  ------------------------------------------------------------------------------------TGGATCTTCTGGGTGGGACCGTTCATCGGCGCGGCG

**HvPIP1;4**  ------------------------------------------------------------------------------------TGGATCTTCTGGGTGGGTCCGTTCATCGGCGCGGCG

**HvPIP1;5**  ------------------------------------------------------------------------------------TGGATCTTCTGGGTGGGCCCCTTCGTTGGCGCCGCC

**HvPIP2;1**  ------------------------------------------------------------------------------------TGGATCTTCTGGGTGGGGCCACTGATCGGCGCCGCC

**HvPIP2;2**  ------------------------------------------------------------------------------------TGGATCTTCTGGGTCGGCCCGTTCGTCGGAGCGCTG

**HvPIP2;3**  ------------------------------------------------------------------------------------TGGATCTTCTGGGTGGGGCCATTCATCGGAGCCGCC

**HvPIP2;4**  ------------------------------------------------------------------------------------TGGATGTTCTGGGTGGGGCCATTCATCGGGGCCGCC

**HvPIP2;5**  ------------------------------------------------------------------------------------TGGATCTTCTGGGTCGGCCCCATGATCGGCGCGGCG

**HvPIP2;6**  ------------------------------------------------------------------------------------TGGATCTTCTGGGTGGGGCCGTTCATCGGCGCGGCG

**HvPIP2;7**  ------------------------------------------------------------------------------------TGGATATTCTGGGTGGGGCCGCTCCTCGGCGCGACG

**HvPIP2;10**  ------------------------------------------------------------------------------------TGGGTCTTTTGGGTCGGGCCCTTCACCGGCGCCGCC

**HvPIP2;8**  ------------------------------------------------------------------------------------TGGATCTTCTGGGTTGGCCCGTTCATCGGCTCCGCC

**HvPIP2;9**  ------------------------------------------------------------------------------------TGGATCTTCTGGGTTGGGCCATTCTCCGGCGCCGCC

**HvPIP2;7a**  GCATGCATATGCCCAAGATCGTCGGACGCATGGGACGGAAGTATACTAGCTCATCGTGTGGGTTGATTTTGTTTTTGCATGCAGTGGATATTCTGGGTGGGGCCGCTCCTCGGCGCGACG

**HvPIP2;11**  ------------------------------------------------------------------------------------TGGATCTTCTGGGTGGGGCCTTTGGCGGGCGCCACC

**HvPIP2;12**  ------------------------------------------------------------------------------------TGGATCTTCTGGGTGGGGCCCTTCATCGGGGCCGGC

**HvPIP2;2a**  TACCACCTTGTGCTTGCAATGCAAGAAGCT------------------------------AACTGTGCATGACATGACATGCAGTGGATCTTCTGGGTCGGCCCGTTCGTCGGAGCGCTG

**OsPIP1;1**  ------------------------------------------------------------------------------------TGGATCTTCTGGGTTGGTCCCTTCGTTGGCGCTGCC

**OsPIP1;2**  ------------------------------------------------------------------------------------TGGATCTTCTGGGTTGGCCCCTTCATTGGCGCTGCC

**OsPIP1;3**  ------------------------------------------------------------------------------------TGGATTTTCTGGGTTGGTCCGTTCATCGGAGCGGCA

**OsPIP2;1**  ------------------------------------------------------------------------------------TGGATCTTCTGGGTCGGCCCGTTCGTCGGCGCCGCC

**OsPIP2;2**  ------------------------------------------------------------------------------------TGGATCTTCTGGGTGGGCCCCCTGATCGGCGCCGCC

**OsPIP2;3**  ------------------------------------------------------------------------------------TGGATCTTCTGGGTGGGGCCCCTCATCGGCGCCGCC

**OsPIP2;4**  ------------------------------------------------------------------------------------TGGATCTTCTGGGTTGGGCCGTTCATCGGCGCGGCG

**OsPIP2;5**  ------------------------------------------------------------------------------------TGGATCTTCTGGGTTGGGCCGTTCATCGGGGCGGCG

**OsPIP2;6**  ------------------------------------------------------------------------------------TGGATCTTCTGGGCGGGGCCGTTCATCGGAGCGCTG

**OsPIP2;7**  ------------------------------------------------------------------------------------TGGATCTTCTGGGTGGGGCCGGTGATCGGGGCGTTC

**OsPIP2;8**  ------------------------------------------------------------------------------------TGGATCTTCTGGGTGGGCCCCTTCGCCGGCGCCGCC

1090 1100 1110 1120 1130 1140 1150 1160 1170 1180 1190

....|....|....|....|....|....|....|....|....|....|....|....|....|....|....|....|....|....|....|....|....|....|....|..

**HvPIP1;1**  CTGGCCGCCATCTACCACCAGGTGGTC---ATCAGAGCGATCCC--------------ATTCA---A-GACCAAGTCCTAA------------------------------------

**HvPIP1;2**  CTGGCGGCCATCTACCACGTGGTGGTG---ATCAGGGCCATCCC--------------CTTCA---A-GAGCCGCGACTAG------------------------------------

**HvPIP1;3**  CTGGCGGCGATCTACCACGTGGTGGTG---ATCAGGGCGATCCC--------------CTTCA---A-GAGCCGCGGCTAG------------------------------------

**HvPIP1;4**  CTGGCGGCCATATACCACGTGGTGGTG---ATCAGGGCAATCCC--------------CTTCA---A-GAGCCGCGACTAG------------------------------------

**HvPIP1;5**  CTGGCAGCCGTCTACCACCAGGTGATC---ATCAGAGCAATTCC--------------GTTCAACAA-GAGCAGGTCCTAG------------------------------------

**HvPIP2;1**  ATTGCCGCCGCCTACCACCAGTATGTG---CTGAGGGCCAGCGC--------------CGC---CAA-GCTCGGGTCCTACAGGAGCAACTAA------------------------

**HvPIP2;2**  GCGGCGGCGGCGTACCACCAGTACATC---CTCCGGGCGGCGGC--------------CATCAAGGC-GCTCGGCTCCTTCCGGAGCAGCCGGAGCA--ACTGA-------------

**HvPIP2;3**  ATTGCCGCCGCCTACCACCAGTACGTC---CTGAGGGCGAGTGC--------------CAC---CAA-GCTTGGCTCGTCTGCCTCCTTCGGTAGGAGCTAG---------------

**HvPIP2;4**  ATCGCCGCCTTGTACCACCAGTACGTG---CTGAGGGCCAGCGC--------------CAC---CAA-GTTCGGCTCGTCTGCCTCCTTCGGCAGCCGCTAG---------------

**HvPIP2;5**  ATCGCGGCCTTCTACCACCAGTACATA---CTCAGGGCCGGCGC--------------CATCAAGGC-CCTCGGCTCCTTCAGGAGCAACGCGTAA---------------------

**HvPIP2;6**  ATCGCAGCGCTCTACCACCAGACCATC---CTCCGCGCGAGCGC--------------CA---GGGG-CTATGGCTCCTTCCGGAGCAACGCCTAG---------------------

**HvPIP2;7**  GTGGCTGCGTTGTACCACCGGTTCGTG---CTGCGCGGCGAGGC--------------CGCCAAGGC-GCTGGGCTCGTTCAGGAGCACCGGCGCCGCCACCGCGCGAACCTAA---

**HvPIP2;10**  ATAGCGGCGTTCTACCACAAGATCGTG---CTGCGCGACGAGGCTGTGGTGAAGGAGTCGCTAACGCAGCTGGGCTCGTTCAAGAGGAGCGGCTCG---ACCGCTTGA---------

**HvPIP2;8**  GTGGCCATGGTGTATCACCAGTACGTC---CTCAGGAACAGCGC--------------CAT-------------CTTCCGGTCCAACTACG---ATGCCGCCGTCTAG---------

**HvPIP2;9**  GTGGCCATGGCGTACCACCAGTACGTC---CTCAGGGGCGGCGC--------------CGCCGCCAA-GCCACACTTCAACTTCGACAACG--GATTCCGACGCCTCGGCTGTTGA-

**HvPIP2;7a**  GTGGCTGCGTTGTACCACCGGTTCGTG---CTGCGCGGCGAGGC--------------CGCCAAGGC-GCTGGGCTCGTTCAGGAGCACCGGCGCCGCCACCGCGCGAACCTAA---

**HvPIP2;11**  GTCGCCATGGCCTACCACCAGTACGTC---CTCAGGAACGGCGC--------------CGCGAAGCA-TTCCTTCGGCCGCTCCGACCACGACGATGTCGAAGCCTAG---------

**HvPIP2;12**  ATCGCCATGGTGTACCACCAGTACATC---ATCAGGGGCGGGGC--------------AGGCAAGGC-ATTAGCCTCCTTCCGCCACAACTACATCGATACCGCCTAG---------

**HvPIP2;2a**  GCGGCGGCGGCGTACCACCAGTACATC---CTCCGGGCGGCGGC--------------CATCAAGGC-GCTCGGCTCCTTCCGGAGCAGCCGGAGCA--ACTGAGCACGCCGGCCAA

**OsPIP1;1**  CTGGCTGCCATCTACCACCAGGTGATC---ATCAGGGCGATCCC--------------ATTCA---A-GAGCAGGTCTTAA------------------------------------

**OsPIP1;2**  CTTGCTGCCATCTACCACCAGGTGGTC---ATCAGGGCAATTCC--------------ATTCA---A-GAGCAGGTCGTAA------------------------------------

**OsPIP1;3**  CTGGCGGCCATCTACCACGTGGTGGTG---ATCAGAGCAATCCC--------------CTTCA---A-GAGCCGGGACTAA------------------------------------

**OsPIP2;1**  ATCGCGGCGTTCTACCACCAGTACATC---CTCCGGGCCGGCGC--------------CATCAAAGC-CCTCGGCTCCTTCAGGAGCAACGCGTGA---------------------

**OsPIP2;2**  ATCGCCGCCGCCTACCACCAGTACGTGGCGCTGAAGTGTAGATA--------------CGCGTACAA-GATGGCCGCCTCTGTCCAGCACTAG------------------------

**OsPIP2;3**  ATCGCCGCCGCCTACCACCAGTACGTC---CTGAGGGCCAGCGC--------------CGC---CAA-GCTCGGCTCTTCCTCCTCCTTCCGCGG---CTAG---------------

**OsPIP2;4**  ATCGCGGCGCTGTACCACCAGGTCATC---CTCCGTGCCAGTGC--------------AA---GGGG-CTACGGCTCCTTCCGGAGCAACGCGTAG---------------------

**OsPIP2;5**  ATCGCAGCGCTGTACCACCAGATCGTC---CTTCGTGCCAGCGC--------------CA---GGGG-GTACGGCTCCTTCCGGAGCAATGCTTAA---------------------

**OsPIP2;6**  GCGGCGGCGGCGTATCACCAGTACATC---CTCCGGGCGGCAGC--------------CATCAAGGC-GCTGGGCTCCTTCCGGAGCAACCCCAGCA--ACTGA-------------

**OsPIP2;7**  TTGGCGGCGGCGTACCACAAGCTGGTG---CTGCGCGGCGAGGC--------------CGCCAAGGC-GCTCAGCTCGTTCAGGAGCACCAGCGTG---ACGGCGTGA---------

**OsPIP2;8**  GCCGCAATGATCTACCATCACTACATC---CTCAGGGGCGCTGC--------------CGCCAAGGC-CTTCGCCTCCTCCTCCTAC--CGATCACCCCATTTTTAA----------

**B. TIPs**

10 20 30 40 50 60 70 80 90 100 110 120

....|....|....|....|....|....|....|....|....|....|....|....|....|....|....|....|....|....|....|....|....|....|....|....|

**OsTIP1;1**  -----------------------ATGCCGATCCGCAACATCGCCGTGGGGAGCCACCAGGAGGTGTA------CCACCCGGGTGCACTCAAGGCGGCGTTGGCCGAGTTCATCTCCACCC

**OsTIP1;2**  -----------------------ATGCCGGTGAGCCGGATCGCGGTGGGCGCCCCCGGCGAGCTGTC------CCACCCGGACACGGCCAAGGCCGCCGTCGCCGAGTTCATCTCTATGC

**OsTIP2;1**  -----------------------------ATGGTGAAGCTCGCATTCGGAAGCTTGGGTGACTCCTT------CAGCGCCACGTCCGTGAAGGCCTACGTGGCGGAGTTCATCGCCACCC

**OsTIP2;2**  --------------------------ATGTCGGGCAACATCGCCTTCGGCCGCTTCGATGACTCCTT------CAGCGCGGCCTCCCTCAAGGCCTACGTCGCCGAGTTCATCTCCACCC

**OsTIP3;1**  --ATGAGCACGGCGGCGGCGAGGCCAGGGCGGCGG---TTCACGGTGGGGCGGAGCGAGGACGCGAC------GCACCCGGACACCATCCGCGCCGCCATCTCCGAGTTCCTCGCCACCG

**OsTIP3;2**  ATGCTGCCAGGTCGCCACACTCCCCGCCGTGCTGACGCCGCCGCCGCTGCCGCCGCCATGGAGCCCCT-----GGTGCCGGGCGCCACCCGCGCGGCGCTGTCGGAGTTCGTAGCCACCG

**OsTIP4;1**  --------------------------ATGGCGAAGGAGGTGGATCCGTGCGACCACGGCGAGGTCG------TCGACGCCGGGTGCGTCCGCGCCGTGCTGGCCGAGCTCGTCCTCACCT

**OsTIP4;2**  --------------ATGCCGTTGCTGCCGATGACGAAGCTGGAGCTCGGCCACCGCGGCGAGGCGT------GGGAGCCCGGCTGCCTCCGCGCCGTCGCCGGCGAGCTCCTCTTCACCT

**OsTIP4;3**  -----------------------------ATGGCGAAGCTCGCGCTCGGCCACCACCGCGAGGCCA------CCGACCCCGGCTGCCTCCGCGCCGTCGTCGCCGAGCTCCTCCTCACCT

**OsTIP5;1**  ---------------------------------ATGGCG---AACATCTGTGCCAACATGAAGCGCTGCTTCTCGCCGCCGGCGC--TCCGGGCGTACTTCGCCGAGTTCTTCTCCACCT

**HvTIP1;1**  -----------------------ATGCCGGTCAGCAGGATCGCCGTGGGGAGCCACCGGGAGGTGTA------CGAGGTCGGCGCCCTCAAGGCGGCGCTCGCCGAGTTCATCTCCACGC

**HvTIP1;2**  -----------------------ATGCCGGTGAGCAGGATCGCCATCGGCGCCCCGGGGGAGCTGTC------CCACCCGGACACCTTCCGCGCCGGCGTCGCCGAGTTCATATCCATGC

**HvTIP2;1**  -----------------------------ATGGTGAAGCTCGCATTCGGGAGCTGCGGTGACTCCTT------CAGCGCCACGTCCATCAGGGCGTACGTGGCGGAGTTCATCGCCACCC

**HvTIP2;2**  -----------------------------ATGGTGAAGCTTGCGTTTGGAAGCTTCGGCGACTCATT------CAGCGCCACGTCCATCAGGTCCTATGTCGCGGAGTTCATCGCCACCC

**HvTIP2;3**  --------------------------ATGCCAGGCTCCATCGCCTTTGGTCGCTTCGATGACTCCTT------CAGCGTGGCCTCTCTCAAGGCCTACGTCGCCGAGTTCATCTCCACCC

**HvTIP3;1**  --ATGAGCACGGCGGCGCGGTCGACGGGGCGGCGGGGGTTCACCATGGGGCGCAGCGAGGACGCGAC------GCACCCGGACACCATCCGCGCCGCAATCTCCGAGTTCCTCGCCACGG

**HvTIP4;1**  --------------------ATGGCCGCCACCAAGCACGCGGATTCGTTCGACGAGCGTGAAGTCGCCGTCGTCGACGCCGGCTGCGTCCGCGCCGTGCTGGGGGAGCTGGTCCTCACCT

**HvTIP4;2**  -----------------------------ATGCCGAAGATAGCGCTCGGCCACCGCCGCGAGGCGT------CGGACCCCGGCTGCGTCCGGGCCGTGCTCGGCGAGCTCGTCCTCACCT

**HvTIP5;1**  ---------------------------------ATGGCGTCCAACCTCCGCGTGCACCTGAAGCACTGCTTCTCGCCGCCGTCCC--TCCGGTCCTACTTCGCCGAGTTCATCTCCACCT

**HvTIP3;2**  ----------------ATGCTGCCAAC--TAGTTTCGCCACCCGCGGGGCCGCGGGGCCGGAGCCCCT-----GCTGCCGGCGGCTTCCCGCGCCGTGCTGTCGGAGTTCGTCGCCACCG

**HvTIP4;3**  -----------------------------ATGGCGAACTTTGCTCTCGGGCACCACCGCGAGGCCA------CCGAGGCCGGCTGCGTCCGCGCCGTTCTCGCCGAGGCCGTGCTCACCT

130 140 150 160 170 180 190 200 210 220 230 240

....|....|....|....|....|....|....|....|....|....|....|....|....|....|....|....|....|....|....|....|....|....|....|....|

**OsTIP1;1**  TCATCTTCGTCTTCGCCGGCCAGGGCTCCGGCATGGCCTTCAGCAAGCTGACCGGCGGCGGAGCGACGAC----CCCCGCCGGGCTGATCGCGGCGGCGGTGGCCCACGCCTTCGCCCTG

**OsTIP1;2**  TCATCTTCGTCTTCGCCGGATCGGGATCCGGCATGGCCTTCAGCAAGCTGACGGACGGCGGCGGCACGAC----GCCGTCGGGGCTGATCGCCGCCTCCCTGGCGCACGCGCTGGCCCTG

**OsTIP2;1**  TCCTCTTCGTCTTCGCTGGCGTCGGATCCGCCATTGCCTATGGGCAATTGACCAATGGTGGCGCCCTCGA----CCCGGCCGGCCTTGTGGCGATCGCGATCGCCCATGCACTCGCCCTG

**OsTIP2;2**  TCGTCTTCGTCTTCGCCGGCGTCGGCTCCGCCATCGCCTACACCAAGTTGACCGGCGGCGCGCCGCTTGA----CCCGGCCGGGCTGGTCGCCGTGGCGGTGTGCCACGGGTTCGGGCTG

**OsTIP3;1**  CCATCTTCGTCTTCGCCGCCGAGGGCTCCATCCTCTCCCTCGGGAAGCTG---TACCAGGACATGAGCAC----GCCGGGAGGGCTGGTGGCGGTGTCGCTGGCGCACGCGCTGGCGCTG

**OsTIP3;2**  CCGTGTTCGTCTTCGCCGCCGAAGGCTCCGTCTACGGCCTCTGGAAGATG---TACAGGGACACGGGGAC----GCTGGGCGGCCTGCTGGTGGTGGCGGTGGCGCACGCGCTGGCGCTG

**OsTIP4;1**  TCGTCTTCGTCTTCACCGGCGTCGCCGCCACCATGGCCGCAGGGGTGCCGGA-GGTGGCGGGGGCGGCGATGCCGATGGCGGCGCTGGCGGGGGTGGCGATCGCGACGGCGCTGGCGGCG

**OsTIP4;2**  TCCTCTTCGTCTTCATCGGCGTCGCCTCCACCATCACCGCCGGGAAGGCGG---CGGGTGGGGCCGGCGA----GGCGGCGGCGGTGACGGCGGCGGCGATGGCGCAGGCGCTGGTGGTG

**OsTIP4;3**  TCCTCTTCGTCTTCTCCGGCGTCGGCTCCGCCATGGCCGCCGCCAAGCTGG---GCGGCGGCGGCGACAC----GATAATGGGGCTGACGGCGGTGGCGGCGGCGCACGCGCTGGTGGTC

**OsTIP5;1**  TCCTCTTTGTGTTCATCGCCGTCGGCTCCACCATCTCCGCCCGGATGCTCACGCCGGATGAAACATCCGA----TGCCTCGTCCCTGATGGCGACCGCCGTTGCGCAGGCGTTCGGGCTC

**HvTIP1;1**  TCATCTTCGTCTTCGCCGGCCAGGGCTCCGGCATGGCCTTCAGCAAGCTGAGCCCCGACGGCGTGGCGAC----GCCGGCCGGGCTGATCTCGGCGGCGATAGCGCACGCGTTCGCGCTG

**HvTIP1;2**  TCATCTTCGTCTTCGCCGGCTCCGGCTCCGGCATGGCCTTCGGCAAGCTGACTGACGGCGGCGCGGCGAC----GCCGGCCGGGCTGATCTCCGCGGCCCTGGCGCACGCGTTCGCGCTT

**HvTIP2;1**  TCCTCTTCGTGTTCGCCGGCGTTGGGTCCGCCATTGCCTATGGGAAACTCACCGAGGACGGCGCCCTCGA----CCCGGCTGGCCTTGTGGCGATCGCGATCGCCCACGCCTTCGCCCTC

**HvTIP2;2**  TCCTCTTCGTGTTCGCCGGCGTCGGGTCCGCCATTTCCTACGGGCAACTGACGCAGGGTGGCGCACTGGA----CCCGGCTGGCCTTGTGGCGATCGCCATCGCCCATGCCTTCGCCCTC

**HvTIP2;3**  TCATCTTCGTCTTCGCCGGCGTCGGCTCTGCCATTGCCTACACCAAGGTGAGCGGCGGCGCGCCCCTTGA----CCCATCCGGGCTGATTGCCGTGGCGATCTGCCACGGGTTCGGGCTG

**HvTIP3;1**  CCATCTTCGTCTTCGCCGCCGAGGGCTCCATCCTCTCCCTCGGGAAGCTC---TACCATGACATGAGCAC----GGCGGGCGGGCTGGTGGCCGTGGCGCTGGCACACGCGCTGGCTCTG

**HvTIP4;1**  TCCTCTTCGTCTTCACCGGAGTCGCCGCCGCCATGGCCGCCGGGGTTCCGGA-GCTGCCGGGCGCGGCTATGCCGATGGCGACGTTGGCCGGGGTTGCGCTTGCGCAGGCGCTGGCAGCG

**HvTIP4;2**  TCCTCTTCGTCTTCGTCGGCGTCGGCTCCGCCATCGTCGGAGGACAAGCAGTCGCAGCCGGAGGCGACCC----GTCGGCGGCGTTGATCGCGGTGGCACTGGGACATGCGCTGGTGGTG

**HvTIP5;1**  TCCTCTTCGTGTTCACCGCCGTCGGCTCCGCCATCTCCGCCAGGATGCTCACGCCCGACGTCACGTCCAA----CGCCTCGTCCCTGGTGGCGACCGCCGTCGCGCAGTCGTTCGGGCTC

**HvTIP3;2**  CCGTGTTCGTCTTCGCCGCCGAAGGCTCCGTCTACGGCCTCTGGAAGATG---TACAAGGACACGGGCAC----GCTGGGGGGCCTGCTCGTGGTGGCGGTGGCGCACGCGCTCGCTCTG

**HvTIP4;3**  TCCTCTTCGTCTTCTCCGGCGTCGGCTCCGCCATGGCCACCGGGAGGCTGG---CCGGCGGCGCCGACAC----GATCATGGGTCTGACGGCAGTCGCGCTGGCCCACGCGATGGTGGTG

250 260 270 280 290 300 310 320 330 340 350 360

....|....|....|....|....|....|....|....|....|....|....|....|....|....|....|....|....|....|....|....|....|....|....|....|

**OsTIP1;1**  TTCGTGGCGGTGTCCGTCGGCGCCAACATCTCCGGCGGCCACGTGAACCCGGCCGTGACCTTCGGCGCCTTCGTCGGCGGCAACATCACCCTGTTCCGCGGCCTCCTCTACTGGATCGCC

**OsTIP1;2**  TTCGTGGCGGTGGCGGTGGGAGCCAACATCTCCGGCGGGCACGTGAACCCGGCGGTGACATTCGGCGCGTTCGTGGGGGGGAACATCAGCCTGGTGAAGGCGGTGGTGTACTGGGTGGCG

**OsTIP2;1**  TTCGTGGGCGTTTCCGTCGCCGCCAACATCTCCGGCGGCCACCTTAACCCGGCCGTGACGTTCGGCCTCGCCGTCGGCGGCCACATCACCATCCTCACCGGACTCTTCTACTGGATCGCC

**OsTIP2;2**  TTCGTGGCGGTGGCCATCGGCGCCAACATCTCCGGCGGCCACGTCAACCCGGCCGTCACCTTCGGCCTCGCCCTCGGCGGCCAGATCACCATCCTCACCGGCGTCTTCTACTGGATCGCC

**OsTIP3;1**  GCGGTGGCGGTGGCGGTGGCCGTCAACATCTCCGGCGGCCACGTCAACCCGGCCATCACCTTCGGCGCGCTCCTCGGCGGCCGCCTCTCCCTCATCCGCGCCCTCTTCTACTGGCTCGCC

**OsTIP3;2**  GCGGCCGCGGTGGCGGTGTCGCGCAACGCGTCGGGCGGGCACGTCAACCCGGCCGTCACGTTCGGCGTGCTCGTCGGCCGCCGCATCTCCTTCGCCCGCGCCGCGCTCTACTGGGCCGCC

**OsTIP4;1**  GGGGTGCTGGTGACGGCGGGGTTCCACGTGTCCGGCGGGCACCTGAACCCGGCGGTGACGGTGGCGCTGCTGGCGCGGGGGCACATCACGGCGTTCAGGTCGGCGCTCTACGTCGCCGCC

**OsTIP4;2**  GCGGTGCTGGCGACGGCGGGGTTCCACGTCTCCGGCGGCCACCTCAACCCGGCCGTGACGCTCTCCCTCGCCGTCGGCGGCCACATCACGCTCTTCCGCTCGGCGCTCTACGTCGCCGCC

**OsTIP4;3**  GCCGTCATGGTCTCCGCGGGGCTGCACGTCTCCGGCGGCCACATCAACCCGGCCGTCACGCTCGGCCTCGCCGCCGGCGGCCACATCACCCTCTTCCGCTCCGCGCTCTACGCCGCCGCC

**OsTIP5;1**  TTCGCCGCGGTGTTCATCGCCGCCGACGTCTCCGGCGGCCACGTCAACCCCGCCGTGACGTTCGCCTACGCCATCGGCGGCCACATCACCGTCCCGAGCGCCATCTTCTACTGGGCGTCT

**HvTIP1;1**  TTCGTGGCGGTGTCGGTGGGCGCCAACATCTCGGGCGGGCACGTGAACCCGGCGGTGACCTTCGGCGCCTTCGTGGGCGGCAACATCACCCTCTTCCGCGGCCTGCTCTACTGGGTGGCG

**HvTIP1;2**  TTCGTGGCCGTCTCCGTGGGCGCCAACATCTCCGGCGGCCACGTGAACCCGGCCGTCACATTCGGCGCCTTCGTGGGCGGCAACATCAGCCTCCTCAAGGCCGTCGTGTACTGGGTGGCG

**HvTIP2;1**  TTCGTTGGTGTCGCAATCGCCGCCAACATCTCCGGCGGCCACCTCAACCCCGCCGTGACCTTCGGCCTTGCCGTCGGCGGCCACATCACCATCCTCACCGGGATCTTCTACTGGGTGGCC

**HvTIP2;2**  TTCGTCGGCGTGGCGATGGCTGCCAACATCTCCGGCGGCCACCTGAACCCCGCCGTCACGTTCGGCCTCGCCGTCGGCGGCCACGTCACCATCCTCACCGGGCTCTTCTACTGGGTCGCC

**HvTIP2;3**  TTCGTCGCGGTTGCCATCGGCGCCAACATCTCCGGCGGCCACGTGAACCCTGCCGTCACCTTCGGCCTCGCGCTCGGCGGTCAGATCACCATCCTCACCGGCCTCTTCTACTGGGTTGCG

**HvTIP3;1**  GCCGTGGCGGTGTCCGTGGCCGTGAACATCTCCGGGGGCCACGTCAACCCGGCCATCACCTTCGGCGCGCTGCTCGGCGGCCGCATCACCCTCGTGCGCGCCCTCTTCTACTGGATCGCG

**HvTIP4;1**  GGGGTGTTGGTGACGGCGGGGTTCCATGTCTCCGGCGGGCACCTCAACCCGGCGGTGACGGTGGCGCTGCTGGCGCGCGGGCACATCACGGCGTTCCGGGCGGTGCTGTACGTGGTGGCC

**HvTIP4;2**  GCGGTGTTCGCGACCGCCGGGTTCCACATCTCCGGCGCCCACATGAACCCGGCCGTCACGCTGAGCCTCGCCGTCGGCGGCCACATCACCCTCTTCCGCGCCGCCTTCTTCGTGCTCGCC

**HvTIP5;1**  TTCGCCGCGGTGTTCATCGCCGCCGACGTCTCCGGCGGCCACGTCAACCCGGCCGTCACGTTCGCCTTCGCCATCGGCGGCCACATCGGCGTCCCGACCGCCATCTTCTACTGGACGTGC

**HvTIP3;2**  GCCGCGGCGGTGGCGCTGGCGAGCGACGCCTCCGGCGGGCACGTCAACCCGGCCGTCACGTTCGGCGTGCTCGTCGGCAGGCGCATCTCCTTTGCGCGCGCCGTGCTCTACTGGGCGGCG

**HvTIP4;3**  GCCGTAATGGTGTCGGCAGGGCTTCACGTCTCCGGCGGCCACATCAACCCAGCCGTCACGCTCTCCCTCGCCGCAGGCGGCCACATCACCCTCTTCCGCTCTGCGCTCTACGTGCTCGCT

370 380 390 400 410 420 430 440 450 460 470 480

....|....|....|....|....|....|....|....|....|....|....|....|....|....|....|....|....|....|....|....|....|....|....|....|

**OsTIP1;1**  CAGCTCCTGGGCTCCACCGTGGCGTGCTTCCTCCTCCGCTTCTCCACCGGCGGG------CTCGCCACCGGCACCTTCGGCCTG---ACCGGCGTCTCCGTGTGGGAGGCCCTGGTGCTG

**OsTIP1;2**  CAGCTGCTGGGGTCGGTGGTGGCGTGCCTGCTGCTCAAGATCGCGACGGGCGGC------GCCGCCGTGGGTGCCTTCTCGCTGTCGGCGGGCGTGGGCGCCTGGAACGCGGTGGTGTTC

**OsTIP2;1**  CAGCTGCTAGGCGCCTCCATCGCCTGCCTCCTCCTCAAGTTTGTCACCCACGGC------AAGGCCATCCCGACGCACGGCGTCGC---CGGCATCAGCGAGCTGGAGGGCGTCGTGATG

**OsTIP2;2**  CAGCTCCTCGGCGCCATCGTCGGCGCCGTCCTCGTCCAGTTCTGCACCGGCGT---------GGCGACACCGACGCACGGGCTGTC---CGGCGTGGGCGCGTTCGAGGGCGTGGTGATG

**OsTIP3;1**  CAGCTCCTCGGCGCCGTCGTCGCCACCCTCCTCCTCCGCCTCACCACCGGCGGC------ATGCGGCCGCCGGGGTTCGCACTGGCGTCGGGGGTGGGGGACTGGCACGCGGTGCTGCTG

**OsTIP3;2**  CAGCTGCTCGGCGCCGTGCTCGCCGTGCTTCTCCTCAGGCTGGCCTCCGGAGGC------ATGCGCCCCATGGGCTTCACGCTCGGCCACCGCATCCACGAGCGGCACGCCCTGCTGCTC

**OsTIP4;1**  CAGCTGCTGGCTTCCTCCCTCGCCTGCATCCTCCTCCGCTACCTCACCGGCGGC------ATGGCGACCCCGGTGCACACTCTGGGCTCAGGGATAGGGCCCATGCAGGGCCTGGTCATG

**OsTIP4;2**  CAGCTCGCCGGCTCCTCCCTCGCGTGCCTCCTCCTCCGGTGCCTCACCGGCGGC------GCCGCCACGCCGGTGCACGCGCTGGCCGACGGGGTGGGCCCCGTCCAGGGCGTGGCGGCG

**OsTIP4;3**  CAGCTGCTCGGCTCCTCCCTCGCCTGCCTCCTCCTCGCCGCCCTCACCGGCGGCGAG---GAGGCCGTCCCGGTGCACGCGCCGGCGCCCGGCGTCGGCGCGGCGCGCGCCGTGGCCATG

**OsTIP5;1**  CAGATGCTCGGCTCCACGTTCGCCTGCCTCGTCCTCCACTACATCTCCGCCGGC------CAGGCCGTGCCGACGACGAGGATCGCGGTGGAGATGACCGGGTTCGGGGCGGGGATCCTG

**HvTIP1;1**  CAGCTGCTGGGCTCCACCGCCGCCTGCTTCCTCCTCCGCTTCTCCACCGGCGGG------CTCCCCACCGGCACCTTCGGGCTC---ACCGGCATCGGCGCGTGGGAGGCGGTGGTCCTG

**HvTIP1;2**  CAGCTCCTCGGCTCCGTCGTTGCCTGCCTCCTCCTCAAGATCGCCACTGGCGGC------GAGGCCGTGGGCGCCTTCTCGCTCTCCGCCGGCGTCGGCGTCTGGAACGCGGTGGTGTTC

**HvTIP2;1**  CAGCTGCTCGGCTCTGCCGCCGCCTGCTTCCTCCTCAAGTTCGTCACCCACGGA------AAGGCCATCCCGACGCACGCTGTGGCGGCCGGCATGAACGAGTTCGAGGGCGTGGTGATG

**HvTIP2;2**  CAGCTGCTCGGCGCCTCCGTGGCATGCCTCCTCCTGCAGTTTGTCACCCACGCC------CAGGCTATGCCGACGCACGCCGTGTC---CGGCATCAGCGAGGTCGAGGGCGTGGTGATG

**HvTIP2;3**  CAGCTCCTCGGTGCCATCGTCGGCGCCTTCCTCGTCCAGTTCTGCACCGGCGT---------GGCGACCCCTACACACGGGCTTTC---CGGCGTGGGCGCTTTTGAGGGCGTGGTGATG

**HvTIP3;1**  CAGCTCCTCGGCGCCATCGTCGCCTCCCTCCTCCTCCGCCTCACCACCGGAGGC------ATGCGGCCGCCCGGTTTCTCCCTGGCGTCGGGGGTGGGGGATTGGCACGCGGTGTTGCTG

**HvTIP4;1**  CAGCTGCTGGCCTCCTCCCTCGCCTGCATCCTCCTCCGGTGCCTGACCGGCGGCCAGCCTACACCGGTTCCGGTGCACACCCTGGGCGCAGGCATAGGCCCCATGCAAGGCCTGGTCATG

**HvTIP4;2**  CAGATGCTCGGCTCCTCCCTCGCCTGCATCCTGCTCAGGGCCCTCACCGGCGGA------CTGGTCACTCCGGTGCACGCGCTGGCGGCGGGCGTGGGCCCGATCCAGGGCCTGGTGGCG

**HvTIP5;1**  CAACTGCTCGGCTCCACGCTCGCCTGCCTCGTCCTCCACTTCCTCTCCGCCGGC------CAGGCCGTGCCGACGACGAGGATCGCTGTGGAGATGACCGGGTTCGGCGCGTCGATAGTG

**HvTIP3;2**  CAGCTTCTCGGCGCCGTGCTCGCCGCCGCTCTCCTCAGGATCATCTCCGGCGGC------GTGCGTCCCATGGGGTTCACGCTCGGCCACGGCATCCACGAGCGGCACGCGCTGCTGCTC

**HvTIP4;3**  CAGCTGCTTGGCTCCTCCCTCGCCTGCCTCCTCCTCGCCTTCCTCGCCGGCAGCGCG---GCGACCATGCCAGTGCACGCGCTGTCCGCCGGGGTGAGCGCGCCGCAGGGAGTGCTCTGG

490 500 510 520 530 540 550 560 570 580 590 600

....|....|....|....|....|....|....|....|....|....|....|....|....|....|....|....|....|....|....|....|....|....|....|....|

**OsTIP1;1**  GAGATCGTCATGACCTTCGGCCTCGTCTACACCGTGTACGCCACCGCCGTTGACCCCA--------------A---GAAGGGCAGCC---TCGGCACCATCGCCC-CCATCGCCATCGGC

**OsTIP1;2**  GAGATCGTCATGACCTTCGGCCTCGTCTACACCGTGTACGCCACCGCGGTGGATCCCA--------------A---GAAGGGCGACC---TCGGTGTCATCGCCC-CCATCGCCATCGGC

**OsTIP2;1**  GAGATCGTCATCACGTTCGCGCTGGTGTACACGGTGTACGCCACGGCGGCGGACCCGA--------------A---GAAGGGATCGC---TCGGCACCATCGCGC-CCATCGCGATCGGC

**OsTIP2;2**  GAGATCATCGTCACCTTCGGGCTGGTGTACACCGTGTACGCCACCGCCGCCGACCCCA--------------A---GAAGGGGTCGC---TCGGCACCATCGCGC-CCATCGCCATCGGC

**OsTIP3;1**  GAGGCGACGATGACGTTCGGGCTCATGTACGCGTACTACGCGACGGTGATCGACCCGA--------------A---GCGCGGGCAC---GTCGGGACCATCGCGC-CGCTCGCCGTCGGC

**OsTIP3;2**  GAGGTCGTCATGACGTTCGGGCTCGTGTACACCGTGTACGCCACCGCCGTCGACCGGA--------------G---AAGCGGCGGC------GGCGACATTGCGC-CCCTCGCCATCGGC

**OsTIP4;1**  GAGATCATCCTAACCTTCTCCCTCCTCTTCGTCGTCTACGCGACCATCCTTGACCCGC--------------G---GAGCTCGGTCC------CGGGCTTCGGCC-CGCTGCTCACGGGC

**OsTIP4;2**  GAGGCCGTGTTCACGTTCACGCTGCTGCTGGTGATCTGCGCCACCATCCTCGACCCGA--------------G---GAGGGCGGCGCC---GCCGGGGACGGGGC-CGCTGCTGACGGGG

**OsTIP4;3**  GAGGCCGTGCTCACCTTCTCCCTCCTCTTCGCCGTCTACGCCACCGTCGTCGACCGGC--------------G---CCGCGCCGTCG------GCGCGCTCGGCC-CGCTCCTCGTCGGC

**OsTIP5;1**  GAGGGGGTGCTCACGTTCATGGTGGTGTACACGGTGCACGTCGCCGGCGACCCGCGGGGTGGAGGCTTCGGCGGCAGGAAGGGGCCCGCGGCGACGGCGCTGGGCGCGCTGGTGGTCGGG

**HvTIP1;1**  GAGATCGTCATGACCTTCGGGCTGGTGTACACGGTGTACGCCACCGCCGTCGACCCCA--------------A---GAAGGGCAGCC---TGGGCACCATCGCGC-CCATCGCCATCGGC

**HvTIP1;2**  GAGATCGTCATGACCTTCGGGCTCGTGTACACAGTGTACGCCACGGCTGTGGACCCCA--------------A---GCGCGGCGACC---TCGGGGTAATCGCGC-CCATCGCCATCGGC

**HvTIP2;1**  GAGATCGTCATCACCTTCGCGCTGGTGTACACGGTGTACGCCACGGCGGCGGACCCCA--------------A---GAAGGGGTCCC---TCGGCACCATCGCGC-CCATCGCGATCGGT

**HvTIP2;2**  GAGATCGTGATCACCTTCGCGCTGGTGTACACGGTGTACGCGACGGCGGCCGACCCCA--------------A---GAAGGGCTCCC---TCGGCACCATCGCGC-CCATGGCGATCGGC

**HvTIP2;3**  GAGATCATCGTCACCTTCGGGCTCGTCTACACCGTGTACGCCACCGCCGCCGACCCCA--------------A---GAAGGGATCCC---TCGGCACCATCGCCC-CCATCGCCATCGGC

**HvTIP3;1**  GAGGCCGTGATGACGTTCGGGCTCATGTACGCCTACTACGCGACGCTGATCGACCCCA--------------A---GAGGGGCCAC---GTGGGCACCATCGGGC-CGCTCGCCGTGGGG

**HvTIP4;1**  GAGATCATCCTCACCTTCTCCCTTCTCTTCGTCGTGTACGCGACCATCCTCGACCCGC--------------G---GACCACGGTGC------CCGGCTACGGAC-CGATGCTCACCGGC

**HvTIP4;2**  GAGGTCGTCTTCACCTTCACGCTGCTCTTCACCATCTACGCGGCCATCCTCGACCCCA--------------A---GAGCGCCGCGC------CAGGGTTCGGCC-CGCTGCTCACCGGC

**HvTIP5;1**  GAGGGGGTGATGACGTTCATGGTGGTGTACACGGTGCACGTCGCCGGCGACCCTCGCGGGCAAGG---------CAGGAAGGGGCTGGCGACGTCGGCGCTGGGCGCGCTGGTGGTCGGG

**HvTIP3;2**  GAGGTCGTCATGACGTTCGGGCTCATGTACACCGTGTACGCCACCGCCGTCGATCGGA--------------A---CCGCGGTGGCAACGTCGGCGCCATCGCGC-CCATCGCCATCGGC

**HvTIP4;3**  GAGGCCGTGCTCACCTTCTCGCTGACCTTCACGGTGTACGCGACCGTCGTGGACCCGC--------------G---CCGGAGCGTCG------GCAACCTCGGGC-CGCTACTGGTGGGC

610 620 630 640 650 660 670 680 690 700 710 720

....|....|....|....|....|....|....|....|....|....|....|....|....|....|....|....|....|....|....|....|....|....|....|....|

**OsTIP1;1**  TTCATCGTCGGCGCGAACATCCTCGTCGGCGGCGCGTTCGACGGCGCCTCCATGAACCCGGCCGTCTCCTTCGGCCCGGCCCTCGTCAGCTGGTCCTGGGAGTCCCAGTGGGTGTACTGG

**OsTIP1;2**  TTCATCGTCGGCGCCAACATCCTCGCCGGCGGCGCCTTCGACGGCGCCTCCATGAACCCCGCCGTCTCCTTCGGCCCCGCCGTCGTCACCGGAGTCTGGGACAACCACTGGGTCTACTGG

**OsTIP2;1**  TTCATCGTCGGCGCCAACATCCTCGCCGCCGGCCCGTTCAGCGGCGGCTCCATGAACCCAGCGCGCTCCTTCGGCCCCGCCGTCGCCGCCGGCAACTTCGCTGGCAACTGGGTCTACTGG

**OsTIP2;2**  TTCATCGTCGGCGCCAACATCCTCGTCGCCGGCCCCTTCTCCGGCGGCTCCATGAACCCGGCGCGCTCCTTCGGCCCCGCCGTCGCCAGCGGCGACTACACCAACATCTGGATCTACTGG

**OsTIP3;1**  TTCCTCCTCGGCGCCAACATGCTCGCCGGGGGCCCGTTCGACGGCGCCGGGATGAACCCGGCGAGGGTGTTCGGCCCGGCGCTCGTCGGCTGGCGCTGGAGGCACCACTGGGTGTACTGG

**OsTIP3;2**  TTGGTCGCGGGCGCGAACATCCTCGCCGGGGGCCCGTTCGACGGCGCGGCCATGAACCCGGCGCGCGCGTTCGGCCCGGCGCTCGTCGGCTGGAACTGGCGCCACCACTGGGTTTACTGG

**OsTIP4;1**  CTCATCGTCGGTGCCAACACCATCGCTGGTGGCAACTTCTCCGGCGCGTCAATGAACCCGGCCCGGTCATTTGGGCCGGCGCTGGCCACTGGAGTGTGGACCCACCACTGGATCTACTGG

**OsTIP4;2**  CTCCTCGTCGGCGCCAACACCGTCGCCGGCGGCGCGCTCACCGGCGCGTCCATGAACCCGGCGAGGTCGTTCGGGCCGGCGCTGGCCACCGGCGAGTGGGCCCACCACTGGGTCTACTGG

**OsTIP4;3**  CTCGTCGTCGGCGCCAACATCCTCGCCGGCGGGCCCTACTCCGGCGCCTCCATGAACCCGGCCCGCTCCTTCGGCCCGGCCCTCGCCGCCGGCGAATGGGCCGACCACTGGATCTATTGG

**OsTIP5;1**  GCCGTGACGGGCGCGTGCGTGCTGGCGGCGGGGTCGCTCACGGGCGCGTCGATGAACCCGGCGCGCTCGTTCGGGCCGGCGGTTGTCAGCGGGCACTACAGCAACCAGGCCGTGTACTGG

**HvTIP1;1**  TTCATCGTCGGCGCCAACATCCTCGTCGGCGGCGCCTTCTCCGGCGCGTCCATGAACCCCGCCGTCTCCTTCGGCCCCGCCCTCGTCAGCTGGGAGTGGGGGTACCAGTGGGTGTACTGG

**HvTIP1;2**  TTCATCGTCGGCGCCAACATCCTGGCCGGCGGAGCCTTTGACGGCGCGTCCATGAACCCCGCCGTCTCCTTCGGCCCCGCCGTGGTCAGCGGCGTCTGGGAGAACCACTGGGTGTACTGG

**HvTIP2;1**  TTCATCGTCGGCGCCAACATCCTCGCCGCCGGCCCCTTCAGCGGGGGCTCCATGAACCCCGCCCGCTCCTTCGGCCCGGCCGTCGCCGCCGGCAACTTCGCTGGCAACTGGGTCTACTGG

**HvTIP2;2**  TTCATCGTCGGCGCCAACATCCTCGCCGCCGGGCCCTTCAGCGGCGGCTCCATGAACCCGGCGCGCTCCTTCGGGCCGGCCGTGGCGGCCGGCAACTTCTCCGGCCACTGGGTGTACTGG

**HvTIP2;3**  TTCATCGTCGGGGCCAACATCCTCGTTGCCGGCCCCTTCTCCGGCGGTTCCATGAACCCTGCACGCTCATTCGGCCCCGCCGTTGCCAGCGGCGACTTCACCAACATCTGGATCTACTGG

**HvTIP3;1**  TTCCTGCTCGGTGCTAACATACTCGCCGGCGGGCCGTTTGATGGCGCCGCGATGAACCCGGCACGGGTCTTCGGGCCGGCGCTCGTCGGGTGGCGGTGGAGGCACCACTGGGTCTACTGG

**HvTIP4;1**  CTTATTGTCGGTGCCAACACCATTGCCGGCGGCAACTTCTCTGGGGCGTCCATGAACCCTGCTCGGTCTTTTGGGCCTGCGTTGGCTACCGGGGTGTGGACCAATCATTGGATCTATTGG

**HvTIP4;2**  CTCCTCGTCGGCGCCAACACCATCGCCGGCGGCGCGCTCACCGGCGCGTCCATGAACCCCGCGAGGTCCTTCGGACCGGCGCTGGCCACAGGAAACTGGGCGAACCACTGGGTCTACTGG

**HvTIP5;1**  CTCGTGACGGGCGCCTGCGTGCTGGCGGCGGGCTCTCTCACGGGGGCGTCCATGAACCCCGCGCGGTCGTTCGGGCCGGCGGTTGTCAGCGGCGACTTCAAGAACCAGGCCGTGTACTGG

**HvTIP3;2**  TTCGTCCTGGGCGCCAACATCCTCGCCGGCGGCCCGTTCGACGGCGCGGCGATGAACCCGGCGCGGGCGTTCGGCCCGGCGCTCGTCGGCTGGACCTGGCGCCACCACTGGGTCTACTGG

**HvTIP4;3**  CTCGTCGTCGGCGCCAACGTGCTCGCCGGAGGGCCGTTCTCCGGCGCGTGTATGAACCCGGCACGTTCATTCGGGCCCGCGCTCGTCTCAGGGATTTGGGCCTGTCAGTGGGTCTATTGG

730 740 750 760 770 780 790 800 810 820 830 840

....|....|....|....|....|....|....|....|....|....|....|....|....|....|....|....|....|....|....|....|....|....|....|....|

**OsTIP1;1**  GTTGGCCCACTCATCGGCGGTGGCCTCGCCGGCGTCATCTACGAGGTCCTCTTCATCT----CCCA---CACCCACGAGCAGCTCCCCACC--ACCGACTACTAA---------------

**OsTIP1;2**  CTCGGCCCCTTCGTCGGCGCCGCCATCGCCGCGCTCATCTACGACATCATCTTCATCG----GCCAGCGCCCGCACGACCAGCTGCCCACC--GCCGACTACTGA---------------

**OsTIP2;1**  GTCGGCCCGCTGATCGGCGGCGGCCTCGCCGGGCTCGTGTACGGCGACGTGTTCATCG-----GCTCATACCAGCCCGTTGCCGACCAG----GACTACGCTTAA---------------

**OsTIP2;2**  GTCGGCCCCCTCGTCGGCGGCGGCCTCGCCGGCCTCGTCTACCGGTACGTCTACATGT-----GCGGCGACCACGCCCCCGTTG-CCAGC---AGCGAGTTCTAA---------------

**OsTIP3;1**  CTCGGCCCCTTCGTCGGCGCCGGCCTCGCCGGGCTTCTCTACGAGTACCTCGTCATCCCGTCAGCCGACGCCGCCCCGCACGGCGGCGCGC--ACCAGCCGCTGGCG-CCG--GAGGACT

**OsTIP3;2**  CTCGGGCCGCTCATCGGCGCCGGGATGGCCGGCGCGCTGTACGAGTTTGTCATGGCGGAGCAGCCTGAGCCGCCGGCAGCTGCAGATACTC--G---GCTTCCCGTGGCCGCCGAGGATT

**OsTIP4;1**  CTCGGGCCGCTGATTGGCGGGCCTCTCGCTGGGCTGGTCTATGAGTCATTGTTCTTGG----TCAAGAGGACCCATGAGC--CTCTGCT------AGATAATTCCTTTTAG---------

**OsTIP4;2**  GTCGGGCCGCTGGCAGGTGGGCCCCTCGCGGTGGTGGCCTACGAACTCCTGTTCATGG----ACGTGGAGG-ACGCCGGTGGGGCCCAC------CAGCCGCTGCCGCAGGAGTGA----

**OsTIP4;3**  GTTGGGCCTCTTATAGGTGGGCCTTTGGCTGGGCTGGTTTATGAGGGCCTCTTCATGG----GCCCGCCCGGCCATGAGCCGCTTCCTAGG--AATGACGGCGACTTCTAA---------

**OsTIP5;1**  GCCGGCCCGATGGTCGGCGCGGCGGTGGCGGCGCTGGTGCATCAAGCCCTGGTCTTCCCGACCGTGCCGGAGCCGGCGCCGGCGCCGGCGACGAACGAGTCGGCGCGCCATGGGAGCGTG

**HvTIP1;1**  GTCGGCCCCCTCATCGGCGGCGGCCTCGCCGGCGTCATCTACGAGCTGCTCTTCATCT----CCCG---CACCCACGAGCAGCTCCCCACC--ACCGACTACTAA---------------

**HvTIP1;2**  CTCGGCCCCTTCGCCGGCGCCGCCATCGCCGCCCTCGTCTACGACATCTGCTTCATCG----GCCAGCGCCCGCACGAGCAGCTCCCCACC--GCCGAGTACTGA---------------

**HvTIP2;1**  GTCGGGCCGCTCATCGGAGGTGGCCTCGCCGGGTTCGTGTACGGCGACGTGTTCATCG-----CGTCCTACCAGCCAGTCGCGGACCAG----GACTACGCGTGA---------------

**HvTIP2;2**  GTCGGACCACTCATCGGTGGCGGCCTCGCCGGGCTCGTCTACGGCGACGTGTTCATCG-----CCTCCTACCAGCCGGTCGGCCACCAGCAG-GAGTACCCATGA---------------

**HvTIP2;3**  GCCGGCCCGCTCATCGGCGGTGGCCTCGCCGGCATCGTCTACCGGTACCTGTACATGT-----GCGACAACCACACCCCCGTCG-CCAGC---AACGACTACTAA---------------

**HvTIP3;1**  CTCGGGCCCTTCCTCGGCTCCGGCATCGCTGGGCTCCTCTACGAGTACGTCGTCATCCCGTCTACAGAGACCGCCGCCCACG------CCC--ACCAGCCGCTCGCG-CCG--GAGGACT

**HvTIP4;1**  GTTGGCCCATTGGTCGGTGGTCCGTTGGCCGGTTTTGTCTATGAGATGGTCTTCATGG----TGAAGAAGACCCACGAGC--CTCTGCT------TGGTTGGGACTTTTAG---------

**HvTIP4;2**  GTCGGCCCGCTCGCCGGCGGCCCCCTCGCCGTGGCCGTCTACGAGTTCGTCTTCGCCG----TCCCGGTGACGCACCAGCAGCTCCCCG------TGGTGGA------------------

**HvTIP5;1**  GCCGGCCCGATGATCGGCGCGGCCGTGGCGGCGCTGGTGCATCAGAACCTGGTGTTCCCGTCCGCGCCGGAGCCGCTGCCGC------------ACGAGT---CGCGCCACGGGAGCGTG

**HvTIP3;2**  GTCGGCCCGCTGATCGGCGCCGGGCTGGCCGGCGCGCTGTACGAGTTTGTCATGGCCGAGCAACCCGTGGAGCCGGCGGCCGCGGCCACCC--GTGGGCTGCCCGTGCCCGCCGAAGATT

**HvTIP4;3**  GTTGGGCCGATGATAGGTGGGCTGCTCGCGGGGCTAGTATATGATGGATTGTTCATGG----TCCGGCCCGGTCATCAGCAGCTTCCGAGC--GAAGGCACCGCCTTCTAG---------

850

....|....|....|...

**OsTIP3;1**  ACTAG-------------

**OsTIP3;2**  ACTGA-------------

**OsTIP5;1**  CAAACGGTGGTCGTGTGA

**HvTIP3;1**  ACTAG-------------

**HvTIP5;1**  GAAACGGTGGTGGTGTGA

**HvTIP3;2**  ATTGA-------------

**C. NIPs**

10 20 30 40 50 60 70 80 90 100 110 120

....|....|....|....|....|....|....|....|....|....|....|....|....|....|....|....|....|....|....|....|....|....|....|....|

**HvNIP1;1** ------------------------------------------------------------------------------------------------------------------------

**HvNIP1;2** -----------------------------------------------------------------------ATGGAGCCCATAAATAGCAGAAGCATACTGATCAACACGCGGATCCAAA

**HvNIP2;1** ------------------------------------------------------------------------------------------------------------------------

**HvNIP2;2** ------------------------------------------------------------------------------------------------------------------------

**HvNIP2;3** ------------------------------------------------------------------------------------------------------------------------

**HvNIP3;1** ------------------------------------------------------------------------------------------------------------------------

**HvNIP3;2** ------------------------------------------------------------------------------------------------------------------------

**HvNIP4;1** ------------------------------------------------------------------------------------------------------------------------

**OsNIP1;1** ------------------------------------------------------------------------------------------------------------------------

**OsNIP1;2** ATGGCGGTGGTGGTCGACGGCGTCTCGCCGCCGTGGTCCAAGGAGGCCGTCGTACACCTCCTGTCCGAATTCAGTAGCCCTGATCACGTAAGTATTAGTGTCACAGCTCACCAACCCTGC

**OsNIP1;3** ------------------------------------------------------------------------------------------------------------------------

**OsNIP1;4** ------------------------------------------------------------------------------------------------------------------------

**OsNIP2;1** ------------------------------------------------------------------------------------------------------------------------

**OsNIP2;2** ------------------------------------------------------------------------------------------------------------------------

**OsNIP3;1** ------------------------------------------------------------------------------------------------------------------------

**OsNIP3;2** ---------------------------------------------------------------------------------------------------ATGGAAGGGGGCAAGATGAGC

**OsNIP3;3** ------------------------------------------------------------------------------------------------------------------------

**OsNIP3;4** ------------------------------------------------------------------------------------------------------------------------

**OsNIP3;5** ----------------------------------------ATGAAAAATAAAAAGCCCACTAATTCTGAGAAAAAAAAAATACAGCTAGCTATCACCGTTCACATTGGAATTGGATATTA

**OsNIP4;1** ------------------------------------------------------------------------------------------------------------------------

130 140 150 160 170 180 190 200 210 220 230 240

....|....|....|....|....|....|....|....|....|....|....|....|....|....|....|....|....|....|....|....|....|....|....|....|

**HvNIP1;1** ------------------------------------------------------------------------------------------------------------------ATGGCA

**HvNIP1;2** CAAGAGATAGAGATAGAGATAGAGATAGAGATAGACAGCCGGACAAAGAGAGAGGGATGAAGGGAGAGAGCGGAAGCGCGCGCATGGCAGGAGGAGGAGGAGAGCATGGAGCCAACGGCC

**HvNIP2;1** ------------------------------------------------------------------------------------------------------------------ATGGCC

**HvNIP2;2** ---------------------------------------------------------------------------------------------------------ATGTCGGTGACTTCC

**HvNIP2;3** ---------------------------------------------------------------------------------------------------------ATGTCGGTGACTTCC

**HvNIP3;1** ---------------------------------------------ATGGAGGCGGCGGCTGGTGCTGG---GGCTGGGGCGGAGACGCCG---AACC---CGTCGGCGCCGGCGACGCCG

**HvNIP3;2** ------------------------------------------------------------------------------------------------------------------------

**HvNIP4;1** ------------------------------------------------------------------------------------------------------------------ATGGAT

**OsNIP1;1** ------------------------------------------------------------------------------------------------------------------ATGGCA

**OsNIP1;2** CTGCTGAAGAACGGCTTGGAGGAGACAGAGACAACTAGGGCAAGTGGTTTGCCAAACTTGCGCAATGATTCGATCAACCGTGTTCTGATTGAGTCTTTGGTTGAAAGATTGATAGCTTTG

**OsNIP1;3** -----------------------------------------------------------------------------------------ATGGCTGGAGGAGAGCATGGAGTTAATGGCC

**OsNIP1;4** -----------------------------------------------------------------------------------------------ATGGCACGGCGAGAAGTCGACGACT

**OsNIP2;1** ------------------------------------------------------------------------------------------------------------------ATGGCC

**OsNIP2;2** ------------------------------------------------------------------------------------------------------------ATGGCATCGACG

**OsNIP3;1** ---------------------------------------ATGGAGATGGCGGCGCCGAACGGGGGCGGCGCGGCGGGGATGTCGTCGCCGGTGAACGGGGCGTCGGCGCCGGCGACGCCG

**OsNIP3;2** AGCATGGGCATGGATGCTG--CTTCAGCTTCTGTTACTGTCCCTCCCATGCAGATGCAGGCAGGTGATCAGAG---CAACAGGATCGCCATCATCATCTCTCCAAGAG--CCGGGAGCTC

**OsNIP3;3** -----------------------------------------------ATGGAAGGGCACAAGAGTGG-CATGG---AAGCTGTA--GCAGTTGCCATCCCTCCC------TTGCACACTG

**OsNIP3;4** --------------------------------ATGGCTGAGAACATGGTGATGGTGAGTAGTAGTGATGAAAATCACAATCAAGTGGCCATCGACTTGTGCTCGGCGTCGCCGGTTGACC

**OsNIP3;5** CACATGCGTGTGCATGTCGGGCTCCAAAACCCATTCTGCTTCAAAGAAAATGGATGAAGGTTCATCACCGGCGTC-CACCTCGGCCACCGCCGCCGCCGCCGCCGCAAATTTGGAGTCCA

**OsNIP4;1** ------------------------------------------------------------------------------------------------------------ATGACAACGGAT

250 260 270 280 290 300 310 320 330 340 350 360

....|....|....|....|....|....|....|....|....|....|....|....|....|....|....|....|....|....|....|....|....|....|....|....|

**HvNIP1;1** GGAGGA--GGCGATA--ACTC-CCAGACGAACGGAGG------AGCTC-AAGAGCCGCGCGCCATGGAGGAAGGGAGGAAGGAGGA-----------------CTATGACCAGGGCT---

**HvNIP1;2** TGCAAG--AGCAAGA--CCACGCCGGAGCTCTGGAAGAA----GGAAG-AGGAGGAGCGAATCACCCAGCAGGGTGCGAAAATTCAG------AGCAAGATCTCATCAGCACCAGC----

**HvNIP2;1** AGCAAC---TCGAGA--TCGAACTCCAGGGCGACCTTCTCCAGCGAGA-TCCACGACATCGGAACGGTGCAGAA-CTCCACCACG----------CCCAGCATGGTGTACTACACCGAG-

**HvNIP2;2** AACACGCCGACGAGG--GCCAACTCGCGAGTGAACTACTCGAACGAGA-TCCACGACCTGTCCACGGTGCAGGA-CGGCGCCCCCAGCCT----CGCCCCCAGCATGTACTACCAGGAG-

**HvNIP2;3** AACACCCCGACTCGG--GCAAACTCGCGAGTGAACTACTCGAACGAGA-TCCACGACCTGTCTACCGTGCAGGA-CGGCGCCCCCAGCCT----CGCCCCCAGCATGTACTACCAGGAG-

**HvNIP3;1** GGGACGCCGGCGCCGCTGTTCGCGGGGCCGCGGGTGGACTCGCTGTCGTACGAGCGCAAGTCGATGCCGCGGTGCAGGTGCCTGCCGGTGG---------AGGCGTGGATGTCGCCCA--

**HvNIP3;2** ------------------------------------------------------------------------------------------------------------------------

**HvNIP4;1** CTTGACAAGACGAA--------CACGGTGGCCGGCGACGGCGCGGCCA-ACGGGCACGATG---TAGAGCAGGCTCGCCGTGGCC---------AGGAGCCGGCGCCACCGCCTGCCGG-

**OsNIP1;1** GGAGGT--GACAACA--ACTC-CCAGACCACCAATGGCGGCTCAGGTC-ACGAGCAGAGAGCCATGGAGGAAGGCAGGAAGCAGGAGG-----AGTTCGCCGCCGACGGCCAGGGCT---

**OsNIP1;2** CCATTG--AGTCAGG--GAGA--GAGATGGCTGGGAGAGAGGATGGAG-CAGCAGCAGGAGCCATGGAAGAAGG-GCAAGACAGCAAG-----GAGATCAAATTTTCAACGAACATTGA-

**OsNIP1;3** AGCATG--AAGAAA--------CCAGAGCTATGGAGGAA----GGCAG-CAGAG------ATCATCAAGCAAGGTGTGAGAATTCAG------AGCAAGATGGAGGAAGCAAGAGCTC--

**OsNIP1;4** CCTAC---ACCAACG--GTTC----CGTGGTCGAGGTCGTGTCCATAG-AGGAAGGCAGCAAGATGGACAAGGAGGATGACCACCAAA-----ACCCGCAGGCGCCTGACGGCGGCGA--

**OsNIP2;1** AGCAACAACTCGAGA--ACAAACTCCAGGGCGAACTACTCCAACGAGA-TCCACGATCTCTCCACGGTGCAGAA-CGGCACCATG----------CCTACCATG---TACTACGGCGAG-

**OsNIP2;2** ACAGCGCCGTCGAGG--ACCAACTCTCGGGTGAACTACTCGAACGAGA-TCCATGACCTCTCCACCGTGCAGAG-CGTCTCCGCC----------GTCCCCAGCGTCTACTACCCCGAG-

**OsNIP3;1** GGGACGCCGGCGCCGCTGTTCGCGGGGCCGCGGGTGGACTCGCTGTCGTACGAGCGCAAGTCGATGCCGCGGTGCAAGTGCCTGCCGGCGGCGGTGGCGGAGGCGTGGGCGCCGTCGGCG

**OsNIP3;2** CAAGATCCTGCCATT-CGAGCTTGTCAATGGTGCCGCCAATGCCGGCTCGCAACGGCACGCTGATCCAGCAGA---ATCCACTCCTGA-----AGCTCATCATCA---TCTATGGCATCC

**OsNIP3;3** GTGAGAGCAACCA---CAGGATTGACAG---------CAATGTCAGCTCGCAATGCCATGCTGATCCTGCAGA---GCTCTCTGATGA-----AACTCAGCAGCAATCTCTGTGGCATC-

**OsNIP3;4** GGAGCCTCTCCGCCGCCGCCGGCGGCAGCACCACC-CCGAGATCACCGGGCTTCTCCATGGTGGTGGTGCCGGTCGAGTCGCCGGAGA-----AGACGACTGGCAAACCTCAGACTGACG

**OsNIP3;5** CCAGTTTCGACGACGGCAGAAGTCATAGCTCCAAGATCACGCCAATCGAGCTGGTCGTCGTCAATCCCGAAGA---ACCGCCACCGGC-----CTCTCGGTCTCGTGGTCATGGACCTC-

**OsNIP4;1** CATGCCGGGAAGAAA--GTCGACGTCGTCGTGGTTGGCAACGTTGACG-GCGAGCACGTCGGAGTAGAGCAAGCTCGCCATGATCTGCACGAGGAGGCGGCGGCGGCGGCGGCGGCTGA-

370 380 390 400 410 420 430 440 450 460 470 480

....|....|....|....|....|....|....|....|....|....|....|....|....|....|....|....|....|....|....|....|....|....|....|....|

**HvNIP1;1** ---------GCGGCCTCGCCATCTCTCTC---CC----CTTCGTCCAGAAGATCATCGCCGAGATTTTCGGGACGTACTTCCTGATCTTCGCCGGGTGCGGCGCGGTGACCATCAACAAG

**HvNIP1;2** --------AACCAGCCCATGATCTCCGTT---CA----ATTCGTGCAGAAGGTCCTCGCCGAGATATTGGGGACGTACCTGCTCATCTTCGCCGGCTGCGCGGCGGTGGCCGTGAACAAG

**HvNIP2;1** -CGGT-----CCATCGCCGACTACTTCCC---TCCTCACCTCCTCAAGAAGGTGGTGTCGGAGGTGGTGTCGACGTTCCTGCTGGTGTTCGTGACGTGCGGGGCGGCGGCCATCAGCGCC

**HvNIP2;2** -AAGT-----CATTCGCCGACTTCTTCCC---TCCCCACCTCCTCAAGAAGGTGATATCGGAGCTGGTGGCGACGTTCCTGCTGGTGTTCGTGACGTGCGGGGCGGCGTCCATCTACGGC

**HvNIP2;3** -AAGT-----CCCTCGCCGACTTCTTCCC---TCCCCACCTCCTCAAGAAGGTGATATCGGAGGTGGTGGCGACGTTCCTGCTGGTGTTCGTGACGTGTGGGGCGGCGTCCATGTACGGC

**HvNIP3;1** -ACGCCTGCGTCGTGGAGATCCCCTCGCCGGACGTCTCGCTCCCCCGCAAGCTTGGCGCGGAGTTCGTGGGAACGTTCATCCTCATCTTCTTCGCGACGGCGGCGCCGATCGTGAACCAG

**HvNIP3;2** ------------------------------------------ATGGATAAGGCGACGGCGGAGTTCCTCGGGACCTTCATCCTGATGTTCACCCAGGTGTCCGCCATCATCATGGACGAG

**HvNIP4;1** -CCAC----GCCACCAAGGGCCTCGCCGT---CGGCCACCTCATCCGGGAGCTGGTGCTGGAGGGCGTGGCGACGTTCCTGGTGGTGTTCTGGTCGTGCGTGGCGGCGCTGATGCAGGAG

**OsNIP1;1** ---------GCGGCCTCGCCTTCTCCGTC---CC----TTTCATCCAGAAGATCATCGCGGAGATCTTTGGGACATACTTCTTGATCTTCGCGGGGTGCGGGGCGGTGACGATCAACCAG

**OsNIP1;2** -TTGCTGACACAGATCCATGGTGCATGTG---CATGAACAAAAACCTGCTGATTCTTGCGGAAATTCTGGGGACATATTTCATGATATTCGCCGGGTGCGGCGCCGTGGTGGTGAACCAG

**OsNIP1;3** -TAGTAATAACCACCCCATGTTCTCTGTC---CA----ATTCGCGCAGAAGGTGATCGCGGAGATCTTGGGGACGTTCTTCCTCATCTTCGCGGGGTGCGCGGCGGTGGCGGTGAACAAG

**OsNIP1;4** -CGTCGTGGTTTGTGGCATG-CCCATGTC---GTTCACCTTCCTCCAGATGCTGCTCGCCGAGTTCTTGGCCACGTTCTTCCTGATGTTCGCGGGGCTGGGCGCCATCACGGTGGAGGAG

**OsNIP2;1** -AAGG-----CCATCGCCGACTTCTTCCC---TCCTCACCTCCTCAAGAAGGTCGTGTCGGAGGTGGTGGCCACGTTCCTGCTGGTGTTCATGACGTGTGGGGCGGCAGGGATCAGCGGC

**OsNIP2;2** -AAAT-----CCTTCGCCGACATCTTCCC---TCCTAACCTCCTCAAGAAGGTGATATCGGAGGTGGTGGCGACGTTCCTGCTTGTGTTCGTGACGTGCGGGGCGGCGTCCATCTACGGC

**OsNIP3;1** CACGGCTGCGTCGTGGAGATCCCGGCGCCGGACGTCTCGCTCACCCGCAAGCTTGGAGCGGAGTTCGTGGGGACGTTCATCCTCATCTTCTTCGCGACGGCGGCGCCGATCGTGAACCAG

**OsNIP3;2** AGTAGACCTCCCAAAGATAAAACCACCA-----GTTCCTCTTGTCAAGAAGGTGGGTGCAGAGTTCTTTGGCACATTCACACTGATCTTCACAGTGCTGTCCACCATCATCATGGATGAA

**OsNIP3;3** --TAGGCCTAAGAAAGATAATACCATCATC-T-GTTCCTCTCCTCAAGAAGGTCAGTGCTGAGTTCTTTGGCACATTCATACTGATCTTCACCGTGCTATCAACCATCATCATGGATGAA

**OsNIP3;4** ATCATGATCAGCAGCAAGGGCGTGCCAAAG-AGGTGCCACTTGTCAAGAAGGCTGCGGCGGAGTTCATCGGCACGTTCATCCTGGTCTTCACGGTGCTGTCCACCGTCGTGATGGACGCG

**OsNIP3;5** --GTCGTCGATCATGGCGGCGGCGGCCATG-T-CCACCCCTCGCGAAGAAGGCGGCGGCCGAGTTCGTGGGCACGTTCATCCTGATCTTCGCCATGCTCTCCACCATCGTGACCGACGCG

**OsNIP4;1** -CCATC-ATGCCACCAGAGGCCTAGCCAT---TGGCTTTCTCATACGAGAGGTGATGGTGGAGGGGTTGGCGTCGTTCTTGGTGGTGTTCTGGTCGTGCGTGGCGGCGCTGATGCAGGAG

490 500 510 520 530 540 550 560 570 580 590 600

....|....|....|....|....|....|....|....|....|....|....|....|....|....|....|....|....|....|....|....|....|....|....|....|

**HvNIP1;1** AGCAAG---GGGCAGATCACGTTCCCCGGCGTAGCCATCGTGTGGGGCCTCGCCGTGATGGTGATGGTGTACTCCGTCGGCCACATCTCCGGCGCGCACTTCAACCCGGCCGTCACCTTC

**HvNIP1;2** AGGACGGCCGGCACGGTGACGTTCCCGGGCATCTGCATCACCTGGGGCCTGGCCGTCATGGTGATGGTCTACTCCGTCGGCCACATCTCCGGGGCGCACCTCAACCCCGCCGTCACGCTC

**HvNIP2;1** CACGACGTCACGCGCATATCGCAGCTCGGCCAGTCGGTCGCCGGCGGGCTCATCGTCGTCGTGATGATCTATGCCGTCGGCCACATCTCCGGCGCCCACATGAACCCCGCCGTCACCCTC

**HvNIP2;2** GCCGACGTGACGCGCGTCTCGCAGCTGGGCCAGTCCGTCGTCGGGGGCCTCATCGTCACCGTCATGATCTACGCCACCGGACACATCTCCGGCGCGCACATGAACCCCGCCGTCACCCTC

**HvNIP2;3** GCCGACGTGACGCGCGTCTCGCAGCTGGGCCAGTCCCTCGTTGGCGGCCTCATCGTCACCGTCATGATCTACGCCACCGGCCACATCTCCGGCGCGCACATGAACCCCGCCGTCACCCTC

**HvNIP3;1** AAGTACGGCGGCGTGATCTCGCCGTTCGGGAACGCGGCGTGCGCGGGCCTGGCGGTGACGACCATCATCCTGTCGACGGGCCACATCTCCGGCGCGCACCTGAACCCGTCGCTGACCATC

**HvNIP3;2** CAGCACGACGGCGTGGAGGGCCTCATGGGCATCGGCGTGTCCGTGGGCCTGGCGGTCACGGTGCTTGTCTTCTCCACCATCCACATATCCGGGTGCCACCTGAACCCCGCGGTGAGCATT

**HvNIP4;1** ATGCACCACGGGCTCACCTTCC------------CCACCGTCTGCCTCGTCGTCGCCCTCACCGTCGCCTTCGTGCTCGGCTGGATGGGCCCCGCGCACCTCAACCCCGCCGTCACCGTC

**OsNIP1;1** AGCAAGAACGGGCAGATCACGTTCCCGGGGGTGGCGATCGTGTGGGGGCTGGCGGTGATGGTGATGGTGTACGCCGTGGGGCACATCTCCGGCGCGCACTTCAACCCCGCGGTGACGCTG

**OsNIP1;2** AGCACCGGCGGCGCGGTGACGTTCCCGGGGATCTGCGCCGTGTGGGGGCTCGTCGTCATGGTGCTAGTCTACACTGTCAGCCACATCTCCGGTGCCCACTTCAACCCCGCCGTCACCGTC

**OsNIP1;3** AGGACGGGAGGCACGGTGACGTTCCCGGGGATCTGCATCACGTGGGGGCTCGCCGTGATGGTGATGGTGTACTCCGTCGGCCACATCTCCGGCGCGCACCTCAACCCGGCGGTCACCCTC

**OsNIP1;4** AAGAAG---GGCGCGGTGACGTTCCCGGGGGTGGCCGTGGCGTGGGGCGCGGCGGTCATGGCGATGGTGTACGCCGTCGGCCACGTCTCCGGCGCGCACCTCAACCCGGCCGTCACCCTC

**OsNIP2;1** AGCGACCTGTCTCGCATATCGCAGCTGGGACAGTCGATCGCCGGTGGCCTCATCGTGACGGTGATGATCTACGCCGTCGGCCACATCTCCGGCGCCCACATGAACCCCGCCGTGACGCTC

**OsNIP2;2** GAGGACATGAAGCGCATCTCGCAGCTGGGGCAGTCGGTGGTCGGTGGCCTCATCGTCACCGTCATGATCTACGCCACCGGCCACATCTCCGGCGCCCACATGAACCCGGCCGTCACCCTC

**OsNIP3;1** AAGTACGGCGGCGCGATCTCGCCGTTCGGGAACGCGGCGTGCGCGGGGCTCGCCGTGACGACCATCATCCTGTCGACGGGGCACATCTCCGGCGCCCACCTCAACCCGTCGCTCACCATC

**OsNIP3;2** CAACACAAAGGTGTAGAGTCCCTCCTCGGCATCGCGACATCGGCAGGATTAGCAGTGACAGTACTGGTTCTGTCACTCATCCACATTTCAGGCTGCCACCTGAACCCGGCTGTCAGCATT

**OsNIP3;3** CAACACAAAAGTATCGAGACGCTCCTCGGAATCGCAACATCTGCAGGCTTAGCAGTCACTGTTCTAGTTCTGTCCCTCATCCACATATCAGGATGCCATCTGAACCCTGCAATCAGCATC

**OsNIP3;4** CGGCACGGCGGCGCCGAGACCCTCGTCGGCGTGGCGGCGTCGGCGGGGCTGGCCGTCGTCGCCGTCGTCCTCTCCGTGGTGCACATCTCGGGCTCCCACCTCAACCCGGCGGTGAGCCTC

**OsNIP3;5** CAGCGCGGCGGCGTGGAGGGCCTCGTCGGCGTGGCGGCGTCCATCGGGCTCGCCGTGGCGGTGCTCGTCATGTCGCTCGCCCACGTCTCCGGCGCCCACATCAACCCGGCGGTCAGCGTC

**OsNIP4;1** ATGTACGGGACGCTGACGTTCC------------CGATGGTGTGCCTGGTGGTGGCGATGACGGTGGCGTTCGTTCTCAGCTGGCTCGGCCCGGCGCACTTCAACCCGGCCGTCACCATC

610 620 630 640 650 660 670 680 690 700 710 720

....|....|....|....|....|....|....|....|....|....|....|....|....|....|....|....|....|....|....|....|....|....|....|....|

**HvNIP1;1** GCGTTCGCCACCGTGCGCCGATTCCCG---TGGCGGCAGGTGCCGGCGTACGTGCTGGCGCAGATGCTGGGGGCCACGCTGGCCAGCGGCACGCTGAGGCTCATGTTCGGCGGGCGCCA-

**HvNIP1;2** GCCTTCGCCACCTGCGGCCGCTTCCCG---TGGAGGCAGGTCCCGGCCTACGCGGCGGCGCAGGTGGTCGGGTCCACGGCGGCGAGCCTCACGCTGCGGCTGCTGTTCGGGAGCGAGCC-

**HvNIP2;1** GCCTTCGCCATCTTCCGCCATTTCCCC---TGGATTCAGGTCCCGTTCTACTGGGCGGCGCAGTTCACGGGCGCCATCTGCGCGTCCTTCGTGCTCAAGGCGGTGCTCCACCCC-ATCAC

**HvNIP2;2** TCCTTCGCCTGCTTCCGGCATTTCCCC---TGGATTCAGGTGCCGTTCTACTGGGCGGCGCAGTTCACGGGGGCGATGTGCGCGGCGTTCGTGCTGCGGGCGGTGCTGCACCCG-ATCAC

**HvNIP2;3** TCCTTCGCCTTCTTCCGGCATTTTCCC---TGGATTCAGGTGCCGTTCTACTGGGCGGCGCAGTTCACGGGGGCGATGTGCGCGGCGTTCGTGCTGCGGGCGGTGCTGCACCCG-ATCAC

**HvNIP3;1** GCCTTCGCGGCGTTCCGCCACTTCCCC---TGGCTCCAGGTCCCGGCCTACGTGACCGTCCAAGTGCTGGGCTCCATCTGCGCCGGGTTCGCGCTCAAGGGCGTCTTCCACCCGTTCCTC

**HvNIP3;2** GCCATGGCGGTCTTCGGGCACCTCCCG---CCGGCACACCTCGTCCCCTACGTCGCCGCGCAGGTGCTGGGATCCACCGCCGCCTCGTTCGTGGGCAAGGCCATCTACCACCCCGTGAAC

**HvNIP4;1** ACCTTCGCCGCCTTCCGCTACTTCCCG---TGGCGCAAGCTGCCGCTCTACGTCGCCATGCAGATCGGCGCTTCCGTGCTCGCCTGCCTCTCCGTCAACGCCATGATGGAGCCGCACGAG

**OsNIP1;1** GCGTTCGCGACGTGCCGGAGGTTCCCT---TGGCGGCAGGTGCCGGCGTACGCGGCGGCGCAGATGCTGGGCGCCACCCTCGCCGCCGGCACGCTCCGGCTCATGTTCGGCGGCCGCCA-

**OsNIP1;2** GCCTTCGCCACGTGCGGACGCTTCCGG---TGGAAGCAGGTGCCATCGTACGTGGTGGCCCAGGTGCTGGGATCCACCATGGCAAGCCTGACGCTGCGCGTGGTGTTCGGCGGCGGCGGC

**OsNIP1;3** GCGTTCGCCACCTGCGGCAGGTTCCCG---TGGCGGCGGGTGCCGGCGTACGCGGCGGCGCAGGTGGCCGGCTCGGCGGCGGCGAGCGCGGCGCTGCGGGCGCTGTTCGGCGGCGCGCC-

**OsNIP1;4** GGCTTCGCCGTCGCCGGCCGCTTCCCG---TGGAGGCGCGCGCCCGCGTACGCGCTGGCGCAGACGGCCGCCGCCACGGCGGCGAGCGTGGTGCTGCGGCTCATGTTCGGCGGCCGGCA-

**OsNIP2;1** GCGTTCGCCGTGTTCAGGCATTTCCCC---TGGATTCAGGTTCCGTTCTACTGGGCGGCGCAGTTCACCGGAGCGATATGCGCGTCGTTCGTGCTCAAGGCGGTGATCCACCCG-GTGGA

**OsNIP2;2** TCCTTCGCCTTCTTCCGGCATTTCCCC---TGGATTCAGGTGCCGTTCTACTGGGCGGCGCAGTTCACGGGGGCGATGTGCGCGGCGTTCGTGCTGCGGGCGGTGCTGTACCCG-ATCGA

**OsNIP3;1** GCCTTCGCCGCGCTCCGCCACTTCCCG---TGGCTGCAGGTCCCGGCGTACGTCGCCGTCCAGGTGCTCGGCTCCATCTGCGCCGGCTTCGCCCTCAAGGGCGTCTTCCACCCCTTCCTC

**OsNIP3;2** GCCATGACTGTCTTTGGTCATCTTCCT---CCTGCTCATCTTCTTCCTTACATTGCTGCACAAATCCTCGGCTCGATCACCGCATCGTTCGCCGTCAAAGGGATGTATCATCCGGTGAAC

**OsNIP3;3** GCCATGGCCGTCTTTGGTCACCTCCCT---TCTGCTCATCTTCTACCTTACATTTCTTCACAAATTCTCGGGGCCGTCGCCGCCTCCTTCGCCGTCAAAGGTCTGTATCATCCGGTGAAC

**OsNIP3;4** GCCATGGCCGCGCTCGGCCACCTCCCG---CCGGCCCACCTCCTCCCCTACGCGGCGGTGCAGACGGCGGCCTCCCTCGCCGCCGCGTTCCTCGCCAAGGGCGTGTACCGGCCGGCGCGG

**OsNIP3;5** GCCATGGCCGCGTTCGGGCGCCTCCAG---CCCGCGCACCTCCTGCCCTACGCCGCCGCGCAGGTCCTCGGCGCCGTCGCCGCCGCCGCCGCCGTCGACGGGATCTTCCATCCGGCGAGC

**OsNIP4;1** ACCTTCGCCGCCTACCGCCGCTTCCCGGTCTGGCCCAAGCTGCCGCTCTACGTCGCCGCCCAGCTCGCCGGCTCGCTCCTCGCCTGCCTCTCCGTCAACGCCGTCATGAGGCCGCGCCAC

730 740 750 760 770 780 790 800 810 820 830 840

....|....|....|....|....|....|....|....|....|....|....|....|....|....|....|....|....|....|....|....|....|....|....|....|

**HvNIP1;1** --------------CGAGCACTT---CCCCGGCACGCTCCCTACCGGGTCCGACGTACAGT---CACTCGTTCTCGAGTTCATCATCACCTTCTACCTCATGTTCGTCATTTCGGGCGTG

**HvNIP1;2** --------------GGAGCACTT---CTTCGGGACGGTGCCGGCCGGGTCCGACGTCCAGT---CGCTGGTGCTGGAGTTCATCATCACCTTCTACCTCATGTTCGTCATCTCCGGAGTC

**HvNIP2;1** CGTGA---------TCGGCACCA--CCGAGCCGGTGGGGCCGCACT-----GGCACGCTC-------TGGTCATCGAGGTCGTCGTCACCTTCAACATGATGTTCGTCACCCTCGCCGTC

**HvNIP2;2** GGTGC---------TGGGGACGA--CCACGCCCACGGGGCCGCACT-----GGCACGCGC-------TCGTCATCGAGATCATCGTCACCTTCAACATGATGTTCATCACCTGCGCCGTC

**HvNIP2;3** GGTGC---------TGGGGACGA--CCACGCCCACGGGGCCGCACT-----GGCACGCGC-------TCGTCATCGAGATCGTCGTCACCTTCAATATGATGTTCGTCACCTGCGCCGTC

**HvNIP3;1** TCCGG---------CGGGGTCAC--CGTCCCCGACGTCGCCATCTC----CACCGCCCAGG---CCCTCTTCACCGAGTTCATCATCACCTTCAACCTTCTCTTCGTCGTCACCGCCGTC

**HvNIP3;2** --------------CCCGGC-------ATCGCCACCGTCCCGAGCGTCGGCACCGTCGAGG---CGTTCGCCGTCGAGTTCATCATCACCTTCGTCCTTCTGTTCGTCATAACCGCTGTG

**HvNIP4;1** GACAA---------CTTCTACGG--CACCGTGCCCAG-GCCGCCCGGGGCCGGCGCCCGGCTGCCGTTCCTCCTGGAGCTCCTCGCCTCCGCCGTGCTCATGATCGTCATCGCCACCGTC

**OsNIP1;1** --------------CGAGCACTT---CCCCGGCACGCTCCCCGCCGGCTCCGACGTGCAGT---CGCTCGTCCTCGAGTTCATCATCACCTTCTACCTCATGTTCGTCATCTCCGGCGTC

**OsNIP1;2** GGCGGCGCGCGCGGGGAGCACTTGTTCTTCGGGACGACGCCGGCGGGGTCGATGGCGCAGG---CGGCGGCGCTGGAGTTCGTCATCTCCTTCTTCCTCATGTTCGTCGTCTCCGGCGTC

**OsNIP1;3** --------------GGAGCACTT---CTTCGGGACGGCGCCGGCCGGGTCCGACGTGCAGT---CGCTGGCGATGGAGTTCATCATCACCTTCTACCTCATGTTCGTCGTCTCCGGCGTC

**OsNIP1;4** --------------CGCGCCCGT---GCCGGCCACGCTGCCGGGCGGCGCCCACGCGCAGT---CGCTCGTCATCGAGTTCGTCATCACCTTCTACCTCATGTTCGTCATCATGGCTGTC

**OsNIP2;1** TGTGA---------TCGGAACCA--CCACGCCCGTGGGGCCGCACT-----GGCACTCGC-------TCGTCGTCGAGGTCATCGTGACGTTCAACATGATGTTCGTCACGCTCGCCGTC

**OsNIP2;2** GGTGT---------TGGGGACGA--CGACGCCGACGGGGCCGCACT-----GGCACGCCC-------TCGTCATCGAGATCGTCGTCACCTTCAACATGATGTTCGTCACCTGCGCCGTT

**OsNIP3;1** TCCGG---------CGGCGTCAC--CGTCCCCGACCCCACCATCTC----CACCGCCCAGG---CCTTCTTCACCGAGTTCATCATCACCTTCAACCTCCTCTTCGTCGTCACCGCCGTC

**OsNIP3;2** --------------CCCGGG-------ATTGTGACGGTGCCAAAGGTTGGGACAGTGGAAG---CATTCTTCCTGGAGTTTGTTACAACGTTTGTCCTACTGTTCATCATCACTGCTCTT

**OsNIP3;3** --------------CCCGGG-------ATTGTCACCGTGCCAAATGTCGGCACGGTTGAGG---CGTTTTTCGTCGAGTTCATAATAACATTTTTTCTACTGTTCATCATCACTGCTCTT

**OsNIP3;4** --------------CCGGCCGTC----ATGGCGACCGTGCCGGCCGCCGGC---GTCGGCG---C------CGGCGAG---------------------GCGTTCGTCGTCG--------

**OsNIP3;5** --------------CGAGGGTGG----ATGGTGAGCGTGCCCAAGGTGGGGACGGTGGAGG---CGTTCTTCCTCGAGTTCGTCACCACGTTTGTTCTCCTCTTCGTCATCACCGCTGTC

**OsNIP4;1** GACCA---------CTTCTACGG--CACGGCGCCCGTCGTCGTCCA----CGGCACCCGCCTCCCCTTCCTCATGGAGTTCCTCGCCTCCGCCGTCCTCATGATCGTCATCGCCACCGTC

850 860 870 880 890 900 910 920 930 940 950 960

....|....|....|....|....|....|....|....|....|....|....|....|....|....|....|....|....|....|....|....|....|....|....|....|

**HvNIP1;1** GCCACCGACAACAGAGCCATCGGGGAGTTGGCAGGGCTGGCCGTGGGTGCAACCATCCTTCTTAATGTGTTGATCGCTGGGCCGGTGTCGGGGGCGTCGATGAACCCGGCGAGGACGGTG

**HvNIP1;2** GCCACCGACAACAGAGCCATTGGTGAGCTCGCCGGTCTGGCCGTTGGAGCTACCGTGCTGCTAAACGTGCTCTTTGCCGGGCCCATATCAGGAGCATCCATGAACCCCGCAAGAACCATC

**HvNIP2;1** GCCACGGACACTAGAGCGGTTGGTGAGTTGGCTGGGTTGGCTGTCGGTTCCTCCGTTTGCATTACCTCCATCTTCGCAGGGGCGGTGTCAGGTGGATCGATGAACCCGGCGAGGACGCTG

**HvNIP2;2** GCCACGGACTCGAGAGCGGTGGGTGAGTTGGCAGGGTTAGCAGTTGGTTCCGCGGTTTGCATTACGTCCATCTTCGCAGGGCCTGTGTCAGGAGGATCGATGAACCCGGCGAGGACCCTG

**HvNIP2;3** GCCACGGATTCTAGGGCGGTGGGTGAGTTGGCGGGGTTAGCAGTTGGTTCCGCGGTTTGCATTACGTCCATCTTCGCAGGGCCTGTGTCAGGAGGATCCATGAACCCGGCGAGGACTCTG

**HvNIP3;1** GCCACCGACACCCGCGCGGTGGGCGAGCTCGCCGGTATCGCGGTGGGAGCCGCTGTGACGCTCAACATCCTCGTGGCCGGGCCGACGACGGGAGGGTCGATGAACCCGGTGAGGACGCTG

**HvNIP3;2** GCCACGGACCCTCATGCAGTGAAAGAACTGATCGCGGTGGCAGTTGGTGCGACTGTAGTGATGAATATTCTCGTCGCGGGGCCATCAACAGGGGCGTCTATGAATCCAGCGCGCACGATT

**HvNIP4;1** GCCA---GGAGCTCCGCCAGCAAGGCGGTTGGAGGGATCGCCATCGGGGCGGCGGTCGGGACGCTGGGGCTGGTGATTGGGCCGGTGTCGGGAGGGTCGATGAACCCGGCGAGGAGCCTT

**OsNIP1;1** GCCACCGACAACCGAGCCATCGGGGAGCTGGCAGGGCTGGCCGTTGGTGCAACCATCCTGCTTAACGTGCTGATTGCTGGGCCGATCTCGGGAGCATCGATGAACCCGGCTCGGAGCCTG

**OsNIP1;2** GCCACGGACAACAGAGCAATCGGTGAATTGGCTGGGCTCGCTGTCGGTGCGACGGTCGCGGTGAACGTGCTCTTCGCAGGGCCGGTGACGGGGGCGTCGATGAACCCGGCTCGGAGCCTT

**OsNIP1;3** GCCACCGACAACCGAGCTATTGGTGAACTAGCTGGCCTTGCCGTTGGAGCTACCGTCTTAGTAAATGTGCTCTTTGCTGGGCCAATATCAGGAGCGTCCATGAACCCGGCCAGGACCATC

**OsNIP1;4** GCCACCGATGACCAAGCGGTGGGTCATATGGCTGGAGTGGCTGTGGGTGGAACCATCATGCTCAATGTGCTGTTTGCTGGGCCGGTGTCGGGGGCGTCGATGAACCCGGCGAGGAGCATT

**OsNIP2;1** GCCACGGACACGAGAGCGGTGGGTGAGTTGGCCGGGTTGGCGGTTGGTTCCGCGGTTTGCATTACGTCCATCTTCGCAGGGGCAATTTCAGGTGGATCGATGAACCCGGCAAGGACGCTG

**OsNIP2;2** GCCACCGACTCCAGAGCGGTGGGTGAGTTGGCGGGGTTAGCAGTCGGTTCCGCGGTTTGCATTACGTCGATCTTCGCAGGGCCGGTGTCAGGAGGATCGATGAACCCGGCGAGGACGCTG

**OsNIP3;1** GCCACCGACACCCGCGCCGTCGGCGAGCTCGCCGGCATCGCCGTCGGCGCCGCCGTCACCCTCAACATCCTCATCGCCGGGCCGACGACAGGAGGGTCGATGAACCCGGTGAGGACGCTG

**OsNIP3;2** GCCACTGACCCCAATGCAGTTAAAGAGCTGATAGCAGTGGCAGTCGGAGCAACAATCATGATGAACGCTCTTGTCGCAGGGCCATCGACAGGAGCATCAATGAATCCAGCAAGAACACTT

**OsNIP3;3** GCCACTGATCCTAATGCAGTGAAAGAACTGATAGCAGTGGCAGTTGGGGCAACAGTCATGATGAACATTCTTGTGGCAGGGCCATCAACAGGAGCATCGATGAATCCGGCGCGTACAATT

**OsNIP3;4** ------------AGAGCA----AGGAGCTGGTGGCCATTGCGATTGCTGCAGCGATCATGATGAACGCTCTCGTCGGAGGGCCGTCGACGGGGCCGTCGATGAACCCGGCGAGGACGATC

**OsNIP3;5** TCTGCCGATCCCAACGCAGTTAAAGAGTTGATAGCAGTGGCAGTCGGTGGTACAGCGATGATGAACGTTCTTGTCGCAGGGCCGTCGACAGGAGCATCAATGAACCCTGCAAGAACACTT

**OsNIP4;1** GCCACCGACGGCACAGCGGGGAAGACGGTGGGAGGGATCGCCATCGGAGCGGCGGTGGGAGGGCTGGGGCTGGTGATCGGGCCGGTGTCGGGAGGGTCGATGAACCCGGCGAGGACGCTG

970 980 990 1000 1010 1020 1030 1040 1050 1060 1070 1080

....|....|....|....|....|....|....|....|....|....|....|....|....|....|....|....|....|....|....|....|....|....|....|....|

**HvNIP1;1** GGGCCGGCGCTGGTGGGGAGCGAGTACAGGTCGATCTGGGTGTACGTGGTGGGCCCGGTGGCCGGAGCCGTGGCCGGGGCGTGGGCGTACAACCTCATCCGCTTCACCAA----CAAGCC

**HvNIP1;2** GGCCCGGCAATGGTTGCCGGCCGATACACCAGCATCTGGTTGTATATCGTTGGCCCAATTAGTGGCGCTGTTGCCGGTGCATGGGCTTACAACCTCATCCGGTTTACCAA----CAAGCC

**HvNIP2;1** GGCCCGGCGCTGGCGAGCAACCGCTACCCTGGCCTCTGGCTCTACTTCCTGGGACCCGTCCTTGGCACGCTCAGCGGGGCCTGGACCTACACCTACATCCGCTTCGAGGA------CCCG

**HvNIP2;2** GCGCCGGCGGTGGCCAGCGGCGTCTACACCGGCCTGTGGATCTACTTTCTCGGCCCCGTCATCGGCACGCTCTCCGGCGCGTGGGTCTACACCTACATCCGCTTCGAGGAGGAGCCCTCC

**HvNIP2;3** GCGCCGGCGGTGGCCAGTGGCGTCTACACTGGCCTCTGGATCTACTTTCTCGGCCCCGTCATCGGCACGCTCTCCGGCGCGTGGGTCTACACCTACATCCGCTTCGAGGAGGCGCCCTCC

**HvNIP3;1** GGGCCGGCGGTCGCGGCGGGGAACTACAGGCAGCTGTGGATATACCTGGTGGCGCCGACGCTGGGCGCGGTGTGCGGCGCCGGCGTGTACAAGCTGGTCAAGCTCAGAGA-----CGTCA

**HvNIP3;2** GGACCGGCGATTGTTATGGGGAGATACACCAGGATTTGGGTTTATCTGCTGGCTCAACCACTGGGTGCTATTGCTGGAGCTGGATCCTACGTTGCAATTAAGCTG---------------

**HvNIP4;1** GGCCCGGCGATCGTCTTCGGGCGGTACACCTCCATCTGGATCTACGTCACCGCGCCCGTCGCCGGCATGCTGCTCGGCGCGCTCTGCAACATGGCCGTCCGACAGTCCGA------CGTG

**OsNIP1;1** GGGCCGGCGATGATCGGCGGCGAGTACAGGTCGATCTGGGTGTACATCGTCGGGCCGGTCGCCGGCGCGGTGGCCGGAGCTTGGGCCTACAACATCATCCGCTTCACCAA----CAAGCC

**OsNIP1;2** GGTCCGGCGATGGTGGCGGGGAGGTACGGCGGCGTGTGGGTGTACGTGGCGGCGCCGGTGAGCGGGACGGTGTGCGGCGCGTGGGCGTACAACCTGCTCCGCTTCACCGA----CAAGCC

**OsNIP1;3** GGACCGGCGATCATCCTCGGCCGGTATACCGGTATCTGGGTGTACATCGCCGGCCCGGTTTTTGGCGCAGTTGCTGGCGCGTGGGCCTACAATCTTATCCGGTTCACCGA----CAAACC

**OsNIP1;4** GGGCCGGCGTTGGTGGGGAGCAAGTACACGGCGCTGTGGGTGTACATCTTGGGACCGTTCGCCGGTGCGGCAGCCGGAGCTTGGGCCTACAGCCTCATCCGCCTCACTGG----CGA-CC

**OsNIP2;1** GGGCCGGCGCTGGCGAGCAACAAGTTCGACGGCCTGTGGATCTACTTCCTGGGCCCAGTCATGGGCACGCTCTCGGGAGCATGGACCTACACCTTCATCCGCTTCGAGGA------CACC

**OsNIP2;2** GCGCCGGCGGTGGCCAGCAACGTCTACACCGGCCTCTGGATCTACTTCCTCGGCCCCGTCGTCGGCACCCTCTCCGGCGCATGGGTCTACACCTACATCCGCTTCGAGGAGGCCCCCGCC

**OsNIP3;1** GGGCCGGCGGTGGCGGCGGGCAACTACCGGCAGCTGTGGATATACCTGATCGCGCCGACGCTGGGCGCGGTCGCCGGCGCCGGCGTGTACACGGCGGTGAAGCTCCGCGA-----CGAGA

**OsNIP3;2** GGCCCGGCCATTGCAACAGGGAGATACACACAGATTTGGGTTTATTTGGTTGCTACCCCACTTGGTGCTGTAGCTGGAGAAGGGTTTTATTTTGCAATCAAGCTGTAG------------

**OsNIP3;3** GGCGCGGCTATTGCCACAGGGAGATATACTCAGATTTGGGTTTACTTGGTGGCAACTCCACTGGGTGCTATAGCTGGAACTGGGGCTTATGTTGCAATTAAGCTGTAG------------

**OsNIP3;4** GGGGCGGCGGTGGCGACGGGCGAGTACAGGCAAATGTGGATCTACCTCGTCGCGCCGCCGCTCGGCGCCATCGCCGGAGCAGCCACTTACACTCTCATCAAGCCCTAA------------

**OsNIP3;5** GGTACAGCCATTGTCGCAGGGAATTACACCCAGATATGGGTCTACATGGTTTCTACCCCTCTGGGTGCAATTGCTGGAACAGGGGCTTATTTTGCAATCAAGCTCTAG------------

**OsNIP4;1** GGGCCGGCGATCGTGCTGGGCAGGTACGACGGCGTGTGGATCTACGTGGTGGCGCCCGTCGCCGGGATGCTGGTCGGCGCGCTCTGCAACCGCGCCGTCAGGCTCTCCCA------CCGC

1090 1100 1110 1120 1130 1140 1150 1160 1170 1180 1190

....|....|....|....|....|....|....|....|....|....|....|....|....|....|....|....|....|....|....|....|....|....|....

**HvNIP1;1** GCTGCGCGAGAT-----CACCAAGAGCACCTCCTTCCTCAGGAGCATGAGCAGGATGAACTCCGTCTCCGTCTAG---------------------------------------

**HvNIP1;2** ACTGCGAGAGAT-----CACCAGGACTGGGTCCTTCTTACGAAGTGCAA---GGATGAGCTAG---------------------------------------------------

**HvNIP2;1** CCCAAGGACG------CGCCCCAGAAGCTCTCCTCCTTCAAGCTCCGGCGGCTGCAGAGCCAG---TCCGTGGCCGCCGACGACG---ACGAGCTCGACCACATCCCCGTCTGA

**HvNIP2;2** GTCAAGGACGGC------CCACAGAAGCTCTCCTCCTTCAAGCTCCGCCGCCTGCAGAGCCAGCGGTCCATGGCCGTCGACGAGTTCGACCATGTCTGA---------------

**HvNIP2;3** GTCAAGGACGGC------CCACAGAAGCTCTCTTCCTTCAAGCTCCGCCGCCTGCAGAGCCAGCGGTCCATGGCCGTCGACGAGTTCGACCATGTCTGA---------------

**HvNIP3;1** ACGGCGAGACGCCGCGCCCGCAGCGCAGCTTCCGACGCTGA-------------------------------------------------------------------------

**HvNIP3;2** ------------------------------------------------------------------------------------------------------------------

**HvNIP4;1** GTCGTCGGCTTC----CTCTGCGGCGGCCGGGGCGCGTCCAGCAGGGTGGTCGTCGTCGGTCG--GTCCGTCGCCTGA------------------------------------

**OsNIP1;1** CCTCCGGGAGAT-----CACCAAGAGCGGCTCCTTCCTCAAGAGCATGAACCGGATGAACTCC---TCCACCTAA---------------------------------------

**OsNIP1;2** GCTGCGTGACAT-----CGCCAACACCGCCTCCTTCCTCAGGAGGAGCTCGCGTAGGAGTTAG---------------------------------------------------

**OsNIP1;3** GCTAAGAGAGAT-----CACCATGACTGCATCCTTCATTAGAAGCACAA---GGAGGAACTAG---------------------------------------------------

**OsNIP1;4** GCACGGATTGA-------------------------------------------------------------------------------------------------------

**OsNIP2;1** CCCAAGGAAGGCT---CCTCCCAGAAGCTCTCCTCCTTCAAGCTGCGCCGCTTGCGGAGCCAGCAGTCCATCGCCGCCGACGACGTCGACGAGATGGAGAACATCCAAGTGTGA

**OsNIP2;2** GCCGCCGGCGGCGCCGCCCCCCAGAAGCTCTCCTCCTTCAAGCTCCGCCGCTTGCAGAGCCAG---TCCATGGCCGCCGACGAGTTCGACAACGTCTAA---------------

**OsNIP3;1** ACGGCGAGACCCCGCGCCCCCAGCGCAGCTTCCGCCGCTGA-------------------------------------------------------------------------

**OsNIP3;2** ------------------------------------------------------------------------------------------------------------------

**OsNIP3;3** ------------------------------------------------------------------------------------------------------------------

**OsNIP3;4** ------------------------------------------------------------------------------------------------------------------

**OsNIP3;5** ------------------------------------------------------------------------------------------------------------------

**OsNIP4;1** ATCGTCGCCTTC----CTCTGCGGCACCTCTGTTGGGATC-GCCGGCTCGCCCTAG----------------------------------------------------------

**D. SIPs**

10 20 30 40 50 60 70 80 90 100 110 120

....|....|....|....|....|....|....|....|....|....|....|....|....|....|....|....|....|....|....|....|....|....|....|....|

**HvSIP1;1** ATGGCGATGGGAGCGGC-----TGTGCGGGAGG---CGGCGGCGGACGGCG--TCGTGACCTTCCTCTGGGTGCTCTGCGTCTCCACGCTCGGCGCCTCCACGGCCGCCGTCACCGCCTA

**HvSIP2;1** ATGGCTCCGGCTCCGGC---GTCGAGCGGCCGAATCCGGCCCTGGCTGGTGGTTGGCGACCTGGTCCTGGCGGTGCTGTGGGTGTGCGC-GGGCGCGCTGGTGAAGCTGGCCGTGTACAA

**OsSIP1;1** ATGGCGGTGG---CGGC-----GGTGCGGGCGG---CGGCGGCGGACGCGG--CGGTGACGTTCCTGTGGGTGCTGTGCGTGTCGACGCTGGGGGCGTCGACGGCGGCGGTGACGTCGTA

**OsSIP2;1** ATGTCGCCCGCTCCGCCGCCGTCGCGCGGCCGCATCCGGCCGTGGCTGGTGGTGGGCGACCTGGTGGTGGCGGCGATGTGGGTGTGCGC-GGGCGCGCTGGTGAAGCTCGCCGTGTACGG

130 140 150 160 170 180 190 200 210 220 230 240

....|....|....|....|....|....|....|....|....|....|....|....|....|....|....|....|....|....|....|....|....|....|....|....|

**HvSIP1;1** CCTCAGCCTGCACGAGGGGATCCACTACGCCCTCCTCGTCACCGTCTCCATCCTCGCCCTCCTCTTATTCGCCTTCAACCTCCTCTGCGACGCCCTCGGCGGCGCCAGCTTCAACCCCAC

**HvSIP2;1** CGTGCTCGGACTTGGGGGCCGCCCGGAGGGAGAAGCCGCCAAGGTCTCGCTCTCTGTCGTCTACATGTTCCTCTTCGCCTGGCTCGAATCCGCCACCGGCGGCGCCTCCTACAACCCGCT

**OsSIP1;1** CCTCCGCATCCACGAGGGGATCCACTACGCGCTCCTCGTCACCGTCTCCCTCCTCTCCGTGCTCCTCTTCGCCTTCAACCTCCTCTGCGACGCCCTCGGCGGCGCCAGCTTCAACCCCAC

**OsSIP2;1** GGTGCTCGGCCTCGGTGGCCGCCCCGAGGCCGACGCCGTCAAGGTGGCGCTCTCCCTCGTCTACATGTTCTTCTTCGCCTGGCTCGAGGGCTTCACCGGCGGTGCCTCCTACAACCCGCT

250 260 270 280 290 300 310 320 330 340 350 360

....|....|....|....|....|....|....|....|....|....|....|....|....|....|....|....|....|....|....|....|....|....|....|....|

**HvSIP1;1** CGGCGTCGCCGCCTTCTACGCCGCAGGCCTCACCAGCCCC---------TCGCTCTTCTCCATCGCGCTCCGCCTACCAGCGCAGGCCGCCGGAGCCGTGGGCGGAGCTCTGGCCATCTC

**HvSIP2;1** CACCGCCATCTCCGGTGCCCTGGCCTCCCGCGGCGGCCCCGCCC---TCTACCTCTTCACCGTCTTCGTACGGGTCCCTGCGCAGGTGATTGGGGCAGTTATTGGAGTGATGCTCATGC-

**OsSIP1;1** CGCCCTCGCCGCCTTCCACGCCGCCGGCCTCTCCTCCCCCCGCCACTCCTCCCTCTTCCCCCTCGCCCTCCGCTTCCCCGCCCAGGCCGCGGGTGCGGTGGGTGGGGCGATGGCGATCTC

**OsSIP2;1** CACCGTCCTCGCCGGTGCCCTCGCATCCCGCGCCGGCCCCTCTC---TCTACCTGTTCGCCGCCTTCGTACGGATGCCTGCGCAGGTGTTTGGGTCGATTCTTGGAGTGAAGCTCATCC-

370 380 390 400 410 420 430 440 450 460 470 480

....|....|....|....|....|....|....|....|....|....|....|....|....|....|....|....|....|....|....|....|....|....|....|....|

**HvSIP1;1** CGAGCTGATGCCCGAGCAGTACAAGCACATGCTCGGCGGACCCTCGCTCAAGGTGGATCCCCACACCGGCGCCGCCGCCGAAGGGGTGCTCACCTTCGTCATCACCTTTGCCGTCCTCTG

**HvSIP2;1** -GATTCGCATTCCCTAAAGTA--GGTAAA--------GGAGCCGCGTTAAACGTCGGTGTTCATCATGGGGCTTTAACTGAAGGACTGGCAACCTTGATGGTTGTTATGGTGTCATTGAC

**OsSIP1;1** GGAGCTGATGCCGGAGCAGTACAAGCACATGCTCGGGGGGCCATCGCTCAAGGTGGATCTCCACACGGGTGCCGCCGCCGAGCTGGTGCTCACCTTCGTCATCACCTTGGCCGTGCTCTG

**OsSIP2;1** -GAGCAGCTCTCCCTAAAGTA--GGCAAA--------GGAGCTCCGTTAAGCGTTGGAGTTCACCATGGAGCGTTAGCTGAAGGGTTGGCAACTTTCATGGTTGTTATAGTGTCGGTGAC

490 500 510 520 530 540 550 560 570 580 590 600

....|....|....|....|....|....|....|....|....|....|....|....|....|....|....|....|....|....|....|....|....|....|....|....|

**HvSIP1;1** CATCATCGTCAAGGGACCCCGCAACCCCATTGTCAAGACAGCCATGCTCTCCGTCTCCACCGTCAGCCTCGTCCTCACCGGCGCCGCATACACCGGACCCTCCATGAACCCTGCCAACGC

**HvSIP2;1** ACTTAAAAAGAAAGAAC---AGGGGTTCTTTGTGAAGACATGGATCGCCAGCATTTGGAAAATGACGATTCATATCCTTAGCTCAGATATTACTGGAGGAATTATGAACCCTGCATCTGC

**OsSIP1;1** GATCATCGTCAAGGGGCCCCGTAATCCCATCGTCAAGACCTGGATGCTCTCCATCTCCACCGTTTGCCTCGTCCTCACCGGCGCTGCCTACACTGGCCCATCCATGAACCCTGCCAATGC

**OsSIP2;1** TCTTAAGAAGAAGGAGATGAAAGGGTTCTTTATGAAGACATGGATCTCGAGCATTTGGAAAATGACATTTCATCTCCTTAGCTCAGATATAACTGGAGGAGTCATGAACCCTGCATCTGC

610 620 630 640 650 660 670 680 690 700 710 720

....|....|....|....|....|....|....|....|....|....|....|....|....|....|....|....|....|....|....|....|....|....|....|....|

**HvSIP1;1** GTTTGGTTGGGCGTATGTTAACAATCAGCACAACACCTGGGAGCAGCTGTATGTGTACTGGATATGCCCCTTCATCGGTGCCATTCTCGCTGCGTGGACCTTCAGGGCCGTGTTTCCGCC

**HvSIP2;1** TTTTGCCTGGGCCTATGCTCGTGGAGATCACACATCATTTGACCACCTACTGGTATATTGGCTTGCACCCCTCCAAGCAACCCTTGTAGGGGTATGGGTGGTTACCTTCTTAACTAAACC

**OsSIP1;1** GTTTGGCTGGGCATATGTGAACAATCGCCACAACACATGGGAGCAATTCTACGTCTACTGGATATGCCCTTTTGTTGGCGCTGTTCTTGCTGCCTGGGTCTTCAGGGCCGTGTTCCCACC

**OsSIP2;1** TTTTGCTTGGGCCTATGCTCGAGGGGATCACACAACATTCGACCACCTACTTGTATATTGGCTTGCACCCCTCCAAGCAACCTTGCTAGGAGTATGGGTCGTGACCTTATTAACTAAACC

730 740 750 760 770

....|....|....|....|....|....|....|....|....|....|..

**HvSIP1;1** ACCGGCCCCTAAGCC----CAAGACCAAGAAAGCATGA--------------

**HvSIP2;1** CAAGAAGACCAAGGAGCAAGAAGCAGATAAAAACAAGAACAAAAAGGAATAG

**OsSIP1;1** ACCGGCACCTAAACC----TAAGGCCAAGAAAGCATGA--------------

**OsSIP2;1** CAAGAAGATAGAGGA---AGAAGCAGATGAAAGCAAAACCAAGAAGGAGTAG

**S1 Figure. Alignments of barley and rice nucleotide sequences**

**A.** PIPs; **B.** TIPs; **C.** NIPs; **D.** SIPs. Multiple alignments were performed using ClustalW2 (<http://www.ebi.ac.uk/Tools/clustalw2/index.html>).
