# Supplementary material for: Identification and Expression Analysis of the Barley (Hordeum vulgare L.) Aquaporin Gene Family
Source: PLoS One. 2015 Jun 9;10(6):e0128025. doi: 10.1371/journal.pone.0128025 (PMC4461243; doi:10.1371/journal.pone.0128025)
Supplement: S2 Fig — (DOCX) [file pone.0128025.s002.docx]

**PIPs**

HvPIP1_1 MEGKEEDVRLGANKYSERQPIGTAAQGS---EDKDYKEPPPAPLFEPGELKSWSFYRAGI 57

HvPIP1_2 MEGKEEDVRLGANRYSERQPIGTAAQGGGA-DEKDYKEPPPAPLFEAEELTSWSFYRAGI 59

HvPIP1_3 MEGKEEDVRLGANRYSEHQPIGTAAQGGGA-DEKDYKEPPPAPFFEAGELTSWSFYRAGI 59

HvPIP1_4 MEGKEEDVRLGANRYSERQPIGTAAQGGGA-DEKDYKEPPPAPLFEAEELSSWSFYRAGI 59

HvPIP1_5 MEGKEEDVRLGANRYSERQPIGTAAQGGG--DDKDYKEPPPAPLFEPGELKSWSFYRAGI 58

HvPIP2_1 ---------MAKDIEAAP-QGG-------EFSSKDYSDPPPAPIVDFEELTKWSLYRAVI 43

HvPIP2_2 ----------MAKEVSE-EPE-HAAPAR-----KDYSDPPPAPLFDMGELRMWSFYRALI 43

HvPIP2_3 ---------MAKDIEAAP-PGG-------EYGAKDYSDPPPAPLFDAEELTKWSLYRAVI 43

HVPIP2_4 ---------MAKDIEAAP-PGG-------EYAAKDYSDPPPAPLFDAEELTKWSLYRAVI 43

HvPIP2_5 ---------MAKDEVMET-GGGG------DFAAKDYTDPPPAPLVDAAELASWSLYRAVI 44

HvPIP2_7 ---------MSKEEVIAGGDTADVAVEK-----APYWDPPPAPLLDTSELTRWSLYRAVI 46

HvPIP2_10 -----MPSPILPAKEVEEVVTANEEVTDIIVQRVPYWDPPAVRALDTSELSTWSLYRALI 55

HvPIP2_8 -------MTMAAAQ-GKLSPDAIDNEVISNGSAKDYLDPPPAPLVDAGELGKWSLYRAVI 52

HvPIP2_6 ---------MGKEVDVSALEAG---------GARDYSDPPPAPLVDVDELGRWSLYRAVI 42

HvPIP2_9 ---------MAAAQQGKHSRDAHGSND-----TKDYLDPPAVRLFDAGGLGQWSLYRAII 46

HvPIP2_7a ---------MSKEEVIAGGDTADVAVEK-----APYWDPPPAPLLDTSELTRWSLYRAVI 46

HvPIP2_2a ----------MAKEVSE-EPE-HAAPAR-----KDYSDPPPAPLFDMGELRMWSFYRALI 43

HvPIP2_11 ---------MAAGD-GKLNTEGNVSSDTTM-STKDYLDPPPTPLVDAGELGKWSLYRATI 49

HvPIP2_12 ---------MVPNN---NTVD------------KDYRDPRPAPLINAGELGKWSLWRAVI 36

* :* : * **::** *

**TM2**

**TM1**

HvPIP1_1 AEFMATFLFLYVTILTVMGYSGAAS---------KCATVGIQGIAWSFGGMIFALVYCTA 108

HvPIP1_2 AEFLATFLFLYISVLTVMGVVGNPSG-------SKCGTVGIQGIAWSFGGMIFVLVYCTA 112

HvPIP1_3 AEFLATFLFLYISVLTVMGVVGNPSG-------SKCGTVGIQGIAWSFGGMIFVLVYCTA 112

HvPIP1_4 AEFLATFLFLYISVLTVMGVVGNPSG-------SKCGTVGIQGIAWSFGGMIFVLVYCTA 112

HvPIP1_5 AEFIATFLFLYVTVLTVMGVSKAPS---------KCATVGVQGIAWSFGGMIFALVYCTA 109

HvPIP2_1 AEFVATLLFLYITVATVIGYKHQSDPT-VNTTDAACSGVGILGIAWAFGGMIFVLVYCTA 102

HvPIP2_2 AEFVATLLFLYITVATVIGYKVQSAA-------DPCAGVGVLGIAWAFGGMIFVLVYCTA 96

HvPIP2_3 AEFVATLLFLYITVATVIGYKHQADPAGPNAADAACSGVGILGIAWAFGGMIFVLVYCTA 103

HVPIP2_4 AEFVATLLFLYITVATVIGYKHQADPAGPNAADAACSGVGILGIAWAFGGMIFVLVYCTA 103

HvPIP2_5 AEFIATLLFLYITVATVIGYKHQTDPAVNSAADAACGGVGVLGIAWAFGGMIFVLVYCTA 104

HvPIP2_7 AEFVATLIFLYVSLATVIGYKSQSSA-------QPCTGVGYLGVAWAFGATIFVLVYCTG 99

HvPIP2_10 GEFTASLILLYVSIATVIGYRNQSSA-----ADERCTGVGYLGVAWSFGATVSVLVYSTS 110

HvPIP2_8 AEFTATLLFVYVAVATVVGHKRQTD------AQA-CSGAGVLGIAWAFGGTIAVLVYCTA 105

HvPIP2_6 AEFVATLLFLYITVSTVIGYKHQTDAS-ASGPDAACGGVGVLGIAWAFGGMIFVLVYCTA 101

HvPIP2_9 AEFTASLLFVYVSIATVIGHKRQTD------ADA-CSGAGVLGIAWAFGGMIAVLVYCTA 99

HvPIP2_7a AEFVATLIFLYVSLATVIGYKSQSSA-------QPCTGVGYLGVAWAFGATIFVLVYCTG 99

HvPIP2_2a AEFVATLLFLYITVATVIGYKVQSAA-------DPCAGVGVLGIAWAFGGMIFVLVYCTA 96

HvPIP2_11 AEFTATLLFVYVAVATVIGHKRQTD------AQA-CSGTGVLGIAWAFGGMIAVLVYCTA 102

HvPIP2_12 AEFTATLLFVYVTVATVIGHKRQTD------GTVGCGGAGILGIAWAFGGMIFVLVYCTA 90

** *:::::*::: **:* * * *:**:** : *** *

**TM3**

**HB**

HvPIP1_1 GISGGHINPAVTFGLFLAR-----KLSLTRAVFYIIMQCLGAICGAGVVKGFQQGL-YMG 162

HvPIP1_2 GISGGHINPAVTFGLFLAR-----KLSLTRAVFYMVMQCLGAICGAGVVKGFQTTL-YMG 166

HvPIP1_3 GISGGHINPAVTFGLFLAR-----KLSLTRAVFYIVMQCLGAICGAGVVKGFQTTL-YQG 166

HvPIP1_4 GISGGHINPAVTFGLFLAR-----KLSLTRAVFYIVMQCLGAICGAGVVKGFQTTL-YQG 166

HvPIP1_5 GISGGHINPAVTFGLFLAR-----KLSLTRAIFYIIMQCLGAICGAGVVKGFQQGL-YMG 163

HvPIP2_1 GVSGGHINPAVTFGLFLAR-----KVSLIRALLYIIAQCLGAICGVGLVKGFQSSY-YVR 156

HvPIP2_2 GISGGHINPAVTFGLLLAR-----KVSLLRAVMYIVAQCAGGIVGAGIVKGIMKDA-YQA 150

HvPIP2_3 GVSGGHINPAVTFGLFLAR-----KVSLVRAVLYIIAQCLGAICGVGLVKGFQSAF-YVR 157

HVPIP2_4 GVSGGHINPAVTFGLFLAR-----KVSLVRAVLYIIAQCLGAICGVGLVKGFQSAF-YVR 157

HvPIP2_5 GISGGHINPAVTFGLFLAR-----KVSLVRALLYMVAQCLGAMCGVGLVKAFQSAY-FVR 158

HvPIP2_7 GVSGGHINPAVTFGLFVGR-----KLSLVRTVLYIVAQCLGAICGAGMVKGIAGAS-YEA 153

HvPIP2_10 GVSGGHINPAVTFALFIAG-----KVTLVRSVLYVVAQCLGAVVGVGIVKGIMKHP-YDD 164

HvPIP2_8 GISGGHINPAVTFGLLLAR-----KVSLPRAFLYMVAQCVGAICGAALVRAVHGGHHYAL 160

HvPIP2_6 GISGGHINPAVTFGLFLAR-----RVSLVRALLYMAAQCLGAVCGVGLVRGFQSGL-YAR 155

HvPIP2_9 GISGGHVNPAVTFGLLLAR-----KLSLPRALLYTSAQCLGAICGAAMVRTVHGAQHYEL 154

HvPIP2_7a GVSGTHARTYICIRSTISLQQDNPHMHTI&&EYKLSSVCCCRWSHQPGGDVRAVRGEEAV 159

HvPIP2_2a GISGGHINPAVTFGLLLAR-----KVSLLRAVMYIVAQCAGGIVGAGIVKGIMKDA-YQA 150

HvPIP2_11 GISGGHINPAVTFGLLLAR-----KVSLPRAFFYMAAQCLGAICGAGLLRAVHGAHHYEL 157

HvPIP2_12 GVSGGHINPAVTFGLLLAR-----KVSLVRALLYMVAQCLGAMCGAGLVRAVHGAQ-YAR 144

*:** * : : : :: : * ::

**TM4**

HvPIP1_1 NGGGAN-------VVASGYTKGSG--------------------LGAEIIGTFVLVYTVF 195

HvPIP1_2 NGGGAN-------SVAPGYTKGDG--------------------LGAEIVGTFVLVYTVF 199

HvPIP1_3 NGGGAN-------SVAAGYTKGDG--------------------LGAEIVGTFVLVYTVF 199

HvPIP1_4 NGGGAN-------SVAAGYTKGDG--------------------LGAEIVGTFVLVYTVF 199

HvPIP1_5 NGGGAN-------VVASGYTKGDG--------------------LGAEIIGTFVLVYTVF 196

HvPIP2_1 YGGGAN-------ELSAGYSKGTG--------------------LAAEIIGTFVLVYTVF 189

HvPIP2_2 NGGGAN-------MVASGFSRGTA--------------------LGAEIVGTFVLVYTVF 183

HvPIP2_3 YGGGAN-------ELSAGYSKGTG--------------------LAAEIIGTFVLVYTVF 190

HVPIP2_4 YGGGAN-------ELSAGYSKGTG--------------------LAAEIIGTFVLVYTVF 190

HvPIP2_5 YGGGAN-------TLAAGYSKGTG--------------------LAAEIIGTFVLVYTVF 191

HvPIP2_7 LGGGAN-------TVADGVSVGAG--------------------LGAEIAGTFVLVYTVL 186

HvPIP2_10 FGGGAN-------AVAGGYSLGAA--------------------LGAEIFGTFVLAYTVF 197

HvPIP2_8 YGGGAN-------ELAPGYSRMAG--------------------LIAEIAGTFVLVYTVF 193

HvPIP2_6 HGGGAN-------EVGAGYSVGTG--------------------LAAEIVGTFVLVYTVF 188

HvPIP2_9 YGGGAN-------EVAPGYSKAGG--------------------LLAEAAGTFLLVYTVF 187

HvPIP2_7a AGAHGAVHRGAVPRRHLRRGHGEGDRGGQLRGPRRRRKHGGRRRLGRRGARGGDRRHVRA 219

HvPIP2_2a NGGGAN-------MVASGFSRGTA--------------------LGAEIVGTFVLVYTVF 183

HvPIP2_11 YGGGAN-------EVAPGYSKAGA--------------------LVAEAAGTFVLVYTVF 190

HvPIP2_12 HGGGAN-------ELAPGYSKVAG--------------------LVAEIVGTFVLVYTVF 177

* ..: : * :

**TM5**

HvPIP1_1 SATDAKRNARDSHVPILAPLPIGFAVFLVHLAT-----------------IPITGTGINP 238

HvPIP1_2 SATDAKRSARDSHVPILAPLPIGFAVFLVHLAT-----------------IPITGTGINP 242

HvPIP1_3 SATDAKRSARDSHVPILAPLPIGFAVFLVHLAT-----------------IPITGTGINP 242

HvPIP1_4 SATDAKRSARDSHVPILAPLPIGFAVFLVHLAT-----------------IPITGTGINP 242

HvPIP1_5 SATDAKRNARDSHVPILAPLPIGFAVFLVHLAT-----------------IPITGTGINP 239

HvPIP2_1 SATDPKRNARDSHIPVLAPLPIGFAVFMVHLAT-----------------IPITGTGINP 232

HvPIP2_2 SATDPKRSARDSHVPVLAPLPIGFAVFMVHLAT-----------------IPITGTGINP 226

HvPIP2_3 SATDPKRSARDSHVPVLAPLPIGFAVFMVHLAT-----------------IPITGTGINP 233

HVPIP2_4 SATDPKRSARDSHVPVLAPLPIGFAVFMVHLAT-----------------IPITGTGINP 233

HvPIP2_5 SATDPKRSARDSHVPVLAPLPIGFAVFMVHLAT-----------------IPITGTGINP 234

HvPIP2_7 SATDPKRTARDSFIPVLVPLPIGFAVFIVHLAT-----------------IPITGTGINP 229

HvPIP2_10 SATDPKRTARDAFVPLVAALPIGLSVFVVHLAT-----------------IPITGTGINP 240

HvPIP2_8 SATDPKRIARDPHVPVLAPLLIGFSVLMAHLAT-----------------IPVTGTGINP 236

HvPIP2_6 SATDSKRNARDSHVPMLAPLPIGFAVFMVHLAT-----------------IPITGTGINP 231

HvPIP2_9 SATDPKRMARDTHVPVLAPLLIGFAVVVAHLAT-----------------IPVTGTGINP 230

HvPIP2_7a GVHRPLRHRPQAHRARLLHPRAGAAAHRLRRVHRAPGHHTHHRHRHQPGQEPRRRRHVQP 279

HvPIP2_2a SATDPKRSARDSHVPVLAPLPIGFAVFMVHLAT-----------------IPITGTGINP 226

HvPIP2_11 SATDPKRMARDSHVPVLAPLLIGFAVLMAHLAT-----------------IPITGTGINP 233

HvPIP2_12 AATDPKRKARDSHVPVLAPLPIGFAVLMVHLAT-----------------IPITGTGINP 220

* : : * : : * ::*

**TM6**

**HE**

HvPIP1_1 ARSLG-------------AAIIYNREHAWS--------------DHWIFWVG-PFIGAAL 270

HvPIP1_2 ARSLG-------------AAIIYNKKQSWD--------------DHWIFWVG-PFTGAAL 274

HvPIP1_3 ARSLG-------------AAIIYNKKQAWD--------------DHWIFWVG-PFIGAAL 274

HvPIP1_4 ARSLG-------------AAIIYNKKQAWD--------------DHWIFWVG-PFIGAAL 274

HvPIP1_5 ARSLG-------------AAIIYNRDHAWN--------------DHWIFWVG-PFVGAAL 271

HvPIP2_1 ARSLG-------------AAVIYNTDKAWD--------------DQWIFWVG-PLIGAAI 264

HvPIP2_2 ARSLG-------------AAVIYNKKAAWD--------------NHWIFWVG-PFVG-AL 257

HvPIP2_3 ARSFG-------------AAVIYNNEKAWD--------------DHWIFWVG-PFIGAAI 265

HVPIP2_4 ARSFG-------------AAVIYNNEKAWD--------------DHWMFWVG-PFIGAAI 265

HvPIP2_5 ARSLG-------------AAVIYNKDKAWD--------------DQWIFWVG-PMIGAAI 266

HvPIP2_7 ARSLG-------------AAVMYNQHKAWK--------------DHWIFWVG-PLLGATV 261

HvPIP2_10 ARSLG-------------AAVLYNQHKTWK--------------QHWVFWVG-PFTGAAI 272

HvPIP2_8 ARSFG-------------AAVVYNNKKAWG--------------DQWIFWVG-PFIGSAV 268

HvPIP2_6 ARSLG-------------AAVIYNGDKAWS--------------DQWIFWVG-PFIGAAI 263

HvPIP2_9 ARSLG-------------AAVVYNNSKAWR--------------EQWIFWVG-PFSGAAV 262

HvPIP2_7a AQGMEGPRKYIYHEFDHDACICPRSSDAWDGSILAHRVG&FCFCMQWIFWVG-PLLGATV 338

HvPIP2_2a ARSLG-------------AAVIYNKKAAWD--------------NHVSYLPSSPLTSTTL 259

HvPIP2_11 ARSLG-------------AAVVYNGKKAWA--------------DQWIFWVG-PLAGATV 265

HvPIP2_12 ARSLG-------------AAVVYNKKKAWD--------------EQWIFWVG-PFIGAGI 252

*: : * : :* : : *: :

**TM6**

HvPIP1_1 AAIYHQVVIRAIPF-K-TKS--------------------------------------- 288

HvPIP1_2 AAIYHVVVIRAIPF-K-SRD--------------------------------------- 292

HvPIP1_3 AAIYHVVVIRAIPF-K-SRG--------------------------------------- 292

HvPIP1_4 AAIYHVVVIRAIPF-K-SRD--------------------------------------- 292

HvPIP1_5 AAVYHQVIIRAIPFNK-SRS--------------------------------------- 290

HvPIP2_1 AAAYHQYVLRASAAK--LGSYRSN----------------------------------- 286

HvPIP2_2 AAAAYHQYILRAAAIKALGSFRSS------RSN-------------------------- 284

HvPIP2_3 AAAYHQYVLRASATK--LGSSASFGRS-------------------------------- 290

HVPIP2_4 AALYHQYVLRASATK--FGSSASFGSR-------------------------------- 290

HvPIP2_5 AAFYHQYILRAGAIKA-LGSFRSNA---------------------------------- 290

HvPIP2_7 AALYHRFVLRGEAAKA-LGSFRSTGAATART---------------------------- 291

HvPIP2_10 AAFYHKIVLRDEAVVKESLTQLGSFKRSGSTA--------------------------- 304

HvPIP2_8 AMVYHQYVLRNSAIFRSNYDAAV------------------------------------ 291

HvPIP2_6 AALYHQTILRASARG--YGSFRSNA---------------------------------- 286

HvPIP2_9 AMAYHQYVLRGGAAAKPHFNFDNGFRRLGC----------------------------- 292

HvPIP2_7a AALYHRFVLRGEAAKA-LGSFRSTGAATART---------------------------- 368

HvPIP2_2a CLQCKKLTVHDMTCSGSSGSARSSERWRRRRTTSTSSGRRPSRRSAPSGAAGATEHAGQ 318

HvPIP2_11 AMAYHQYVLRNGAAKHSFGRSDHDDVEA------------------------------- 293

HvPIP2_12 AMVYHQYIIRGGAG-KALASFRHNYIDTA------------------------------ 280

:

**TIPs**

**TM1**

HvTIP1_1 MPVSRIAVGSHR-------EVYEVGALKAALAEFISTLIFVFAGQGSGMAFSK-LSPDGV 52

HvTIP1_2 MPVSRIAIGAPG-------ELSHPDTFRAGVAEFISMLIFVFAGSGSGMAFGK-LTDGGA 52

HvTIP2_1 --MVKLAFGSCG-------DSFSATSIRAYVAEFIATLLFVFAGVGSAIAYGK-LTEDGA 50

HvTIP2_2 --MVKLAFGSFG-------DSFSATSIRSYVAEFIATLLFVFAGVGSAISYGQ-LTQGGA 50

HvTIP2_3 -MPGSIAFGRFD-------DSFSVASLKAYVAEFISTLIFVFAGVGSAIAYTK-VSGGAP 51

HvTIP3_1 MSTAARSTGRRGFTMGRSEDATHPDTIRAAISEFLATAIFVFAAEGSILSLGK-LYHD-M 58

HvTIP3_2 --MLPTSFATRG---AAGPEPLLPAASRAVLSEFVATAVFVFAAEGSVYGLWK-MYKD-T 53

HvTIP4_1 --MAATKHADSF--DEREVAVVDAGCVRAVLGELVLTFLFVFTGVAAAMAAGVPELPGAA 56

HvTIP4_2 --MPKIALG-----HRREAS--DPGCVRAVLGELVLTFLFVFVGVGSAIVGGQAVAAGGD 51

HvTIP4_3 --MANFALG-----HHREAT--EAGCVRAVLAEAVLTFLFVFSGVGSAMATGR--LAGGA 49

HvTIP5_1 -MASNLRVHLK--------HCFSPPSLRSYFAEFISTFLFVFTAVGSAISARM-LTPDVT 50

:: * : :*** :

**TM3**

**HB**

**TM2**

HvTIP1_1 ATPAGLISAAIAHAFALFVAVSVGANISGGHVNPAVTFGAFVGGNITLFRGLLYWVAQLL 112

HvTIP1_2 ATPAGLISAALAHAFALFVAVSVGANISGGHVNPAVTFGAFVGGNISLLKAVVYWVAQLL 112

HvTIP2_1 LDPAGLVAIAIAHAFALFVGVAIAANISGGHLNPAVTFGLAVGGHITILTGIFYWVAQLL 110

HvTIP2_2 LDPAGLVAIAIAHAFALFVGVAMAANISGGHLNPAVTFGLAVGGHVTILTGLFYWVAQLL 110

HvTIP2_3 LDPSGLIAVAICHGFGLFVAVAIGANISGGHVNPAVTFGLALGGQITILTGLFYWVAQLL 111

HvTIP3_1 STAGGLVAVALAHALALAVAVSVAVNISGGHVNPAITFGALLGGRITLVRALFYWIAQLL 118

HvTIP3_2 GTLGGLLVVAVAHALALAAAVALASDASGGHVNPAVTFGVLVGRRISFARAVLYWAAQLL 113

HvTIP4_1 MPMATLAGVALAQALAAGVLVTAGFHVSGGHLNPAVTVALLARGHITAFRAVLYVVAQLL 116

HvTIP4_2 -PSAALIAVALGHALVVAVFATAGFHISGAHMNPAVTLSLAVGGHITLFRAAFFVLAQML 110

HvTIP4_3 DTIMGLTAVALAHAMVVAVMVSAGLHVSGGHINPAVTLSLAAGGHITLFRSALYVLAQLL 109

HvTIP5_1 SNASSLVATAVAQSFGLFAAVFIAADVSGGHVNPAVTFAFAIGGHIGVPTAIFYWTCQLL 110

* *: : : ** *:***:* : : *:*

**TM4**

**TM3**

HvTIP1_1 GSTAACFLLRFSTGGLPT--GTFGLTG-IGAWEAVVLEIVMTFGLVYTVYAT---AVDPK 166

HvTIP1_2 GSVVACLLLKIATGGEAV--GAFSLSAGVGVWNAVVFEIVMTFGLVYTVYAT---AVDPK 167

HvTIP2_1 GSAAACFLLKFVTHGKAI--PTHAVAAGMNEFEGVVMEIVITFALVYTVYAT---AADPK 165

HvTIP2_2 GASVACLLLQFVTHAQAM--PTHAVS-GISEVEGVVMEIVITFALVYTVYAT---AADPK 164

HvTIP2_3 GAIVGAFLVQFCT-GVAT--PTHGLS-GVGAFEGVVMEIIVTFGLVYTVYAT---AADPK 164

HvTIP3_1 GAIVASLLLRLTTGGMRP--PGFSLASGVGDWHAVLLEAVMTFGLMYAYYAT---LIDPK 173

HvTIP3_2 GAVLAAALLRIISGGVRP--MGFTLGHGIHERHALLLEVVMTFGLMYTVYAT---AVDRN 168

HvTIP4_1 ASSLACILLRCLTGGQPTPVPVHTLGAGIGPMQGLVMEIILTFSLLFVVYAT---ILDPR 173

HvTIP4_2 GSSLACILLRALTGGLVT--PVHALAAGVGPIQGLVAEVVFTFTLLFTIYAA---ILDPK 165

HvTIP4_3 GSSLACLLLAFLAGSAAT-MPVHALSAGVSAPQGVLWEAVLTFSLTFTVYAT---VVDPR 165

HvTIP5_1 GSTLACLVLHFLSAGQAV--PTTRIAVEMTGFGASIVEGVMTFMVVYTVHVAGDPRGQGR 168

: :: : : : : * : ** : : : : :

**TM6**

**HE**

**TM5**

HvTIP1_1 KG-SLGTIAPIAIGFIVGANILVGGAFSGASMNPAVSFGPALVSWEWGYQWVYWVGPLIG 225

HvTIP1_2 RG-DLGVIAPIAIGFIVGANILAGGAFDGASMNPAVSFGPAVVSGVWENHWVYWLGPFAG 226

HvTIP2_1 KG-SLGTIAPIAIGFIVGANILAAGPFSGGSMNPARSFGPAVAAGNFAGNWVYWVGPLIG 224

HvTIP2_2 KG-SLGTIAPMAIGFIVGANILAAGPFSGGSMNPARSFGPAVAAGNFSGHWVYWVGPLIG 223

HvTIP2_3 KG-SLGTIAPIAIGFIVGANILVAGPFSGGSMNPARSFGPAVASGDFTNIWIYWAGPLIG 223

HvTIP3_1 RG-HVGTIGPLAVGFLLGANILAGGPFDGAAMNPARVFGPALVGWRWRHHWVYWLGPFLG 232

HvTIP3_2 RGGNVGAIAPIAIGFVLGANILAGGPFDGAAMNPARAFGPALVGWTWRHHWVYWVGPLIG 228

HvTIP4_1 TT--VPGYGPMLTGLIVGANTIAGGNFSGASMNPARSFGPALATGVWTNHWIYWVGPLVG 231

HvTIP4_2 SA--APGFGPLLTGLLVGANTIAGGALTGASMNPARSFGPALATGNWANHWVYWVGPLAG 223

HvTIP4_3 RS--VGNLGPLLVGLVVGANVLAGGPFSGACMNPARSFGPALVSGIWACQWVYWVGPMIG 223

HvTIP5_1 KGLATSALGALVVGLVTGACVLAAGSLTGASMNPARSFGPAVVSGDFKNQAVYWAGPMIG 228

: *:: ** : * : * **** ****: : :** **: *

**TM6**

HvTIP1_1 GGLAGVIYELLFIS---------RTHEQLPTTDY-- 250

HvTIP1_2 AAIAALVYDICFIGQ--------RPHEQLPTAEY-- 252

HvTIP2_1 GGLAGFVYGDVFIA---------SYQPV-ADQDYA- 249

HvTIP2_2 GGLAGLVYGDVFIA---------SYQPVGHQQEYP- 249

HvTIP2_3 GGLAGIVYRYLYMC---------DNHTPVASNDY-- 248

HvTIP3_1 SGIAGLLYEYVVIPS-TETAAHAHQ--PLAPEDY-- 263

HvTIP3_2 AGLAGALYEFVMAEQPVEPAAAATRGLPVPAEDY-- 262

HvTIP4_1 GPLAGFVYEMVFMVK--------KTHEPLLGWDF-- 257

HvTIP4_2 GPLAVAVYEFVFAVP--------VTHQQLPVV---- 247

HvTIP4_3 GLLAGLVYDGLFMVR--------PGHQQLPSEGTAF 251

HvTIP5_1 AAVAALVHQNLVFPSAPEPLPHESRHGSVETVVV-- 262

:* ::

**NIPs**

HvNIP1_1 --------------------------------------------MAGGGDNSQTNGGAQE 16

HvNIP1_2 MEPINSRSILINTRIQTRDRDRDRDRDRQPDKERGMKGESGSARMAGGGGEHGANGLQEQ 60

HvNIP3_1 ----------------MEAAAGAGAGAETPNPSAPATPGTPAPLFAGPRVDSLSYERKSM 44

HvNIP3_2 ------------------------------------------------------------

HvNIP2_2 ---------------------------------MSVTSNTPTRANSRVNYSNEIHDLSTV 27

HvNIP2_3 ---------------------------------MSVTSNTPTRANSRVNYSNEIHDLSTV 27

HvNIP2_1 -----------------------------------MASNS--RSNSRATFSSEIHDIGTV 23

HvNIP4_1 ------------------------------------MDLDKTNTVAGDGAANGHDVEQAR 24

**TM1**

HvNIP1_1 --PRAMEEGRK-EDYDQGCG------------LAISLPFVQKIIAEIFGTYFLIFAGCGA 61

HvNIP1_2 DHAGALEEGRGGANHPAGCENSEQDLISTSNQPMISVQFVQKVLAEILGTYLLIFAGCAA 120

HvNIP3_1 PRCRCLPVEAWMSPNACVVE-----------IPSPDVSLPRKLGAEFVGTFILIFFATAA 93

HvNIP3_2 ---------------------------------------MDKATAEFLGTFILMFTQVSA 21

HvNIP2_2 QDGAPSLAPSMYYQEKSFAD-------------FFPPHLLKKVISELVATFLLVFVTCGA 74

HvNIP2_3 QDGAPSLAPSMYYQEKSLAD-------------FFPPHLLKKVISEVVATFLLVFVTCGA 74

HvNIP2_1 QN--STTPSMVYYTERSIAD-------------YFPPHLLKKVVSEVVSTFLLVFVTCGA 68

HvNIP4_1 RGQEPAPPPAGHATKGLAVG-----------------HLIRELVLEGVATFLVVFWSCVA 67

: * *::::* *

**TM3**

**HB**

**TM2**

**TM1**

HvNIP1_1 VTINK-SKGQITFPGVAIVWGLAVMVMVYSVGHISGAHFNPAVTFAFATVRRFPWRQVPA 120

HvNIP1_2 VAVNKRTAGTVTFPGICITWGLAVMVMVYSVGHISGAHLNPAVTLAFATCGRFPWRQVPA 180

HvNIP3_1 PIVNQKYGGVISPFGNAACAGLAVTTIILSTGHISGAHLNPSLTIAFAAFRHFPWLQVPA 153

HvNIP3_2 IIMDEQHDGVEGLMGIGVSVGLAVTVLVFSTIHISGCHLNPAVSIAMAVFGHLPPAHLVP 81

HvNIP2_2 ASIYGADVTRVSQLGQSVVGGLIVTVMIYATGHISGAHMNPAVTLSFACFRHFPWIQVPF 134

HvNIP2_3 ASMYGADVTRVSQLGQSLVGGLIVTVMIYATGHISGAHMNPAVTLSFAFFRHFPWIQVPF 134

HvNIP2_1 AAISAHDVTRISQLGQSVAGGLIVVVMIYAVGHISGAHMNPAVTLAFAIFRHFPWIQVPF 128

HvNIP4_1 ALMQEMHHGLT----FPTVCLVVALTVAFVLGWMGPAHLNPAVTVTFAAFRYFPWRKLPL 123

: : : : *:**::: ::* :* ::

**TM3**

**TM4**

HvNIP1_1 YVLAQMLGATLASGTLRLMFGGRHEHFPGTLP--TGSDVQSLVLEFIITFYLMFVISGVA 178

HvNIP1_2 YAAAQVVGSTAASLTLRLLFGSEPEHFFGTVP--AGSDVQSLVLEFIITFYLMFVISGVA 238

HvNIP3_1 YVTVQVLGSICAGFALKGVFHP-FLSGGVTVPDVAISTAQALFTEFIITFNLLFVVTAVA 212

HvNIP3_2 YVAAQVLGSTAASFVGKAIYHP-VNPGIATVP--SVGTVEAFAVEFIITFVLLFVITAVA 138

HvNIP2_2 YWAAQFTGAMCAAFVLRAVLHP-ITVLGTTTP--TGPHWHALVIEIIVTFNMMFITCAVA 191

HvNIP2_3 YWAAQFTGAMCAAFVLRAVLHP-ITVLGTTTP--TGPHWHALVIEIVVTFNMMFVTCAVA 191

HvNIP2_1 YWAAQFTGAICASFVLKAVLHP-ITVIGTTEP--VGPHWHALVIEVVVTFNMMFVTLAVA 185

HvNIP4_1 YVAMQIGASVLACLSVNAMMEPHEDNFYGTVPRPPGAGARLPFLLELLASAVLMIVIATV 183

* * : * : * * ::: ::::

**TM5**

**HE**

**TM6**

HvNIP1_1 TDNRAIGELAGLAVGATILLNVLIAGPVSGASMNPARTVGPALVGSEYRSIWVYVVGPVA 238

HvNIP1_2 TDNRAIGELAGLAVGATVLLNVLFAGPISGASMNPARTIGPAMVAGRYTSIWLYIVGPIS 298

HvNIP3_1 TDTRAVGELAGIAVGAAVTLNILVAGPTTGGSMNPVRTLGPAVAAGNYRQLWIYLVAPTL 272

HvNIP3_2 TDPHAVKELIAVAVGATVVMNILVAGPSTGASMNPARTIGPAIVMGRYTRIWVYLLAQPL 198

HvNIP2_2 TDSRAVGELAGLAVGSAVCITSIFAGPVSGGSMNPARTLAPAVASGVYTGLWIYFLGPVI 251

HvNIP2_3 TDSRAVGELAGLAVGSAVCITSIFAGPVSGGSMNPARTLAPAVASGVYTGLWIYFLGPVI 251

HvNIP2_1 TDTRAVGELAGLAVGSSVCITSIFAGAVSGGSMNPARTLGPALASNRYPGLWLYFLGPVL 245

HvNIP4_1 ARSSASKAVGGIAIGAAVGTLGLVIGPVSGGSMNPARSLGPAIVFGRYTSIWIYVTAPVA 243

: * : :*:*::: : * :* **** *:: **: * :*:*

HvNIP1_1 GAVAGAWAYNLIRFTNKP--LREITKSTSFLRSMSRMNSVSV---------- 278

**TM6**

HvNIP1_2 GAVAGAWAYNLIRFTNKP--LREITRTGSFLRS-ARMS-------------- 333

HvNIP3_1 GAVCGAGVYKLVKLRDVN--GETPRPQRSFRR-------------------- 302

HvNIP3_2 GAIAGAGSYVAIKL-------------------------------------- 212

HvNIP2_2 GTLSGAWVYTYIRFEEEPSVKDGPQKLSSFKLRRLQSQRSMAVD-EFDHV-- 300

HvNIP2_3 GTLSGAWVYTYIRFEEAPSVKDGPQKLSSFKLRRLQSQRSMAVD-EFDHV-- 300

HvNIP2_1 GTLSGAWTYTYIRFEDPP--KDAPQKLSSFKLRRLQSQSVAADDDELDHIPV 295

HvNIP4_1 GMLLGALCNMAVRQSDVVVGFLCGGRGASSRVVVVGRSVA------------ 283

* : ** ::

**SIPs**

HvSIP1_1 MAMGAAVREAAADGVVTFLWVLCVSTLGASTAAVTAYLSLH--EGIHYALLVTVSILALL 58

**TM2**

**TM1**

HvSIP2_1 MAPAPASSGRIRPWLVVGDLVLAVLWVCAGALVKLAVYNVLGLGGRPEGEAAKVSLSVVY 60

** * :* ** * : * : * : * **: :

**TM3**

**TM2**

HvSIP1_1 LFAFNLLCDALGGASFNPTG--VAAFYAAGLTSPSLFSIALRLPAQAAGAVGGALAISEL 116

HvSIP2_1 MFLFAWLESATGGASYNPLTAISGALASRGGPALYLFTVFVRVPAQVIGAVIGVMLMRFA 120

:* * * * ****:** *: : * : **:: :*:*** *** * : :

**TM5**

**TM4**

HvSIP1_1 MPEQYKHMLGGPSLKVDPHTGAAAEGVLTFVITFAVLCIIVKGPRNPIVKTAMLSVSTVS 176

HvSIP2_1 FPKVGK----GAALNVGVHHGALTEGLATLMVVMVSLTLKKK-EQGFFVKTWIASIWKMT 175

:*: * * :*:* * ** :**: *::: : * : * : :*** : *: ::

**TM6**

**TM5**

HvSIP1_1 LVLTGAAYTGPSMNPANAFGWAYVNNQHNTWEQLYVYWICPFIGAILAAWTFRAVFPP-- 234

HvSIP2_1 IHILSSDITGGIMNPASAFAWAYARGDHTSFDHLLVYWLAPLQATLVGVWVVTFLTKPKK 235

: : : ** **** ** *** :* ::::* ***: *: ::: * : *

HvSIP1_1 ----PAPKPKTKKA 244

HvSIP2_1 TKEQEADKNKNKKE 249

* * * **

**S2 Figure. Alignments of putative aquaporin protein sequences of barley**

Trans-membrane helices (TM1-TM6) and the two short helices forming the two NPAs (HB and HE) (shaded), P1-P5 residues [22] (shown in blue), ar/R selectivity filter residues (shown in green) and NPA motifs (highlighted in a rectangle). ‘&’ indicates a premature stop codon. Residues suggested to be involved in water transport specificity ([24]; [25]) are shown in red. The N-terminal diacidic motifs important for ER export are underlined. The residues at the position corresponding to Leu197 from SoPIP2;1, determined to be the key residue involved in gating [30] (occurring just before TM5) are shown using an arrow. The positions corresponding to Ser115 and Ser274 in SoPIP2;1 [30] are highlighted in green. The residues corresponding to Ser262 in GmNOD26 [46] are highlighted in a red rectangle. The positions corresponding to Ser283 in AtPIP2;1 [31] are highlighted in red. The residues corresponding to Cys80 from ZmPIP2;1 is highlighted in a dashed rectangle. The residues corresponding to His193 in SoPIP2;1, involved in pH-dependent gating are highlighted in yellow. Asterisks (*) identify conserved nucleotides and colons (:) are conservative nucleotide substitutions.
