# Supplementary material for: Identification and Expression Analysis of the Barley (Hordeum vulgare L.) Aquaporin Gene Family
Source: PLoS One. 2015 Jun 9;10(6):e0128025. doi: 10.1371/journal.pone.0128025 (PMC4461243; doi:10.1371/journal.pone.0128025)
Supplement: S3 Fig — (DOCX) [file pone.0128025.s003.docx]

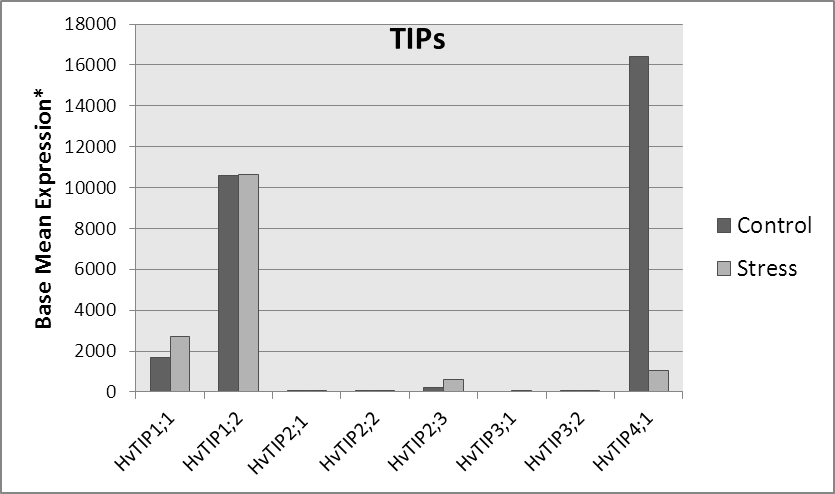

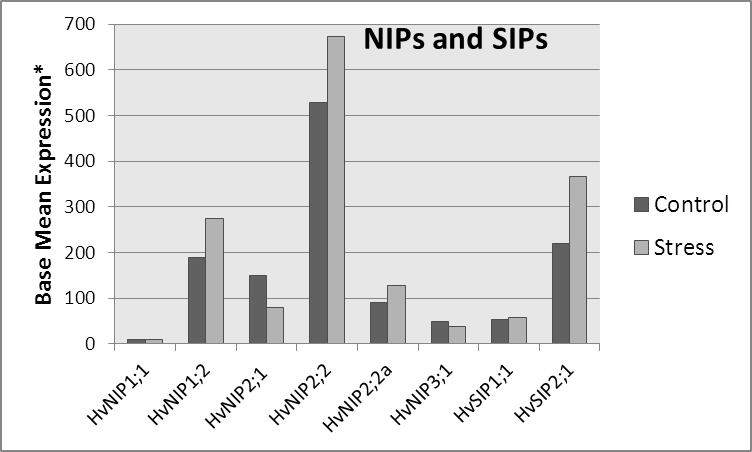

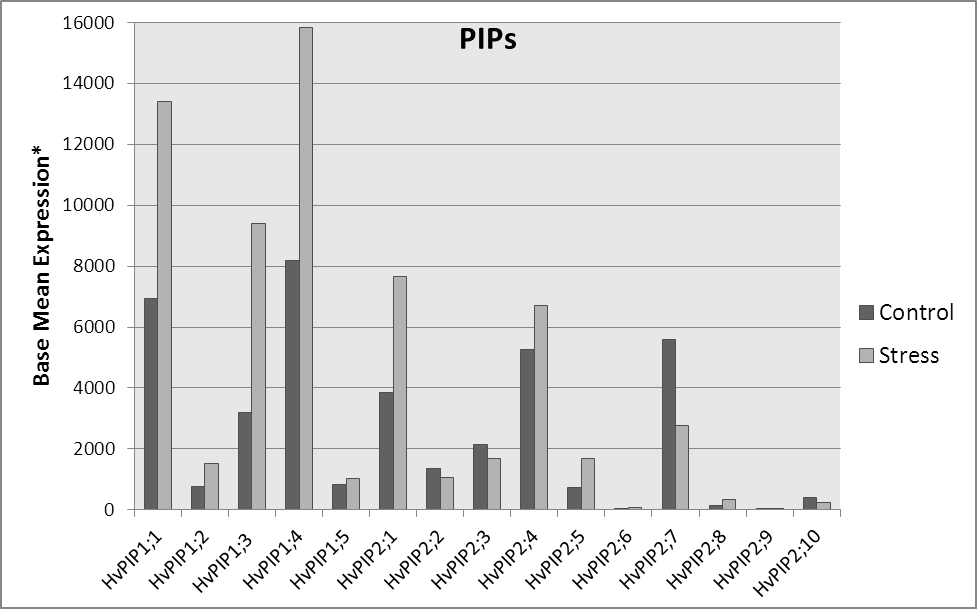

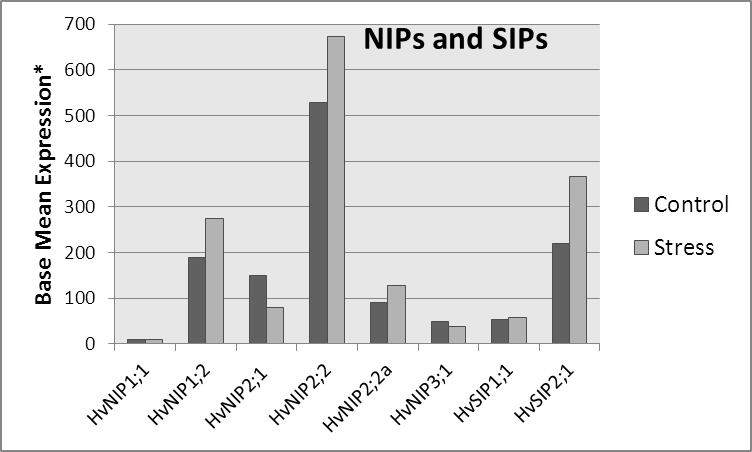


**S3 Figure. Expression levels of barley aquaporins from leaf mRNA-seq data**

*Base mean expression is the normalised sequence read counts normalised to the total library size.
