# Supplementary material for: Identification and Expression Analysis of the Barley (Hordeum vulgare L.) Aquaporin Gene Family
Source: PLoS One. 2015 Jun 9;10(6):e0128025. doi: 10.1371/journal.pone.0128025 (PMC4461243; doi:10.1371/journal.pone.0128025)
Supplement: S4 Fig — (DOCX) [file pone.0128025.s004.docx]

**
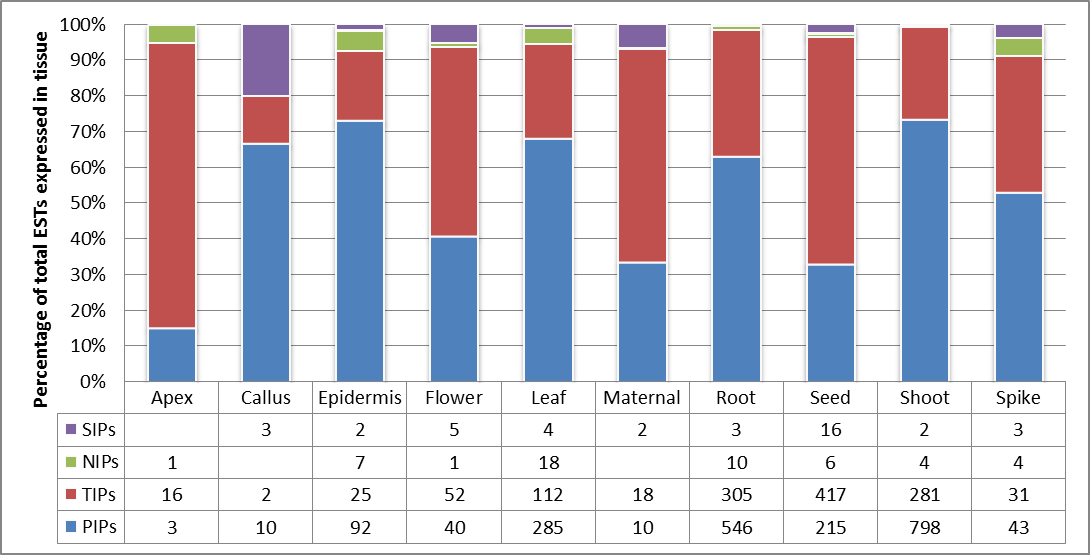
**

**S4 Figure. Summary of ESTs of the aquaporin sub-families in different tissue types.**

The numbers represent the number of ESTs identified from NCBI in a particular tissue type.
