# Supplementary material for: Identification and Expression Analysis of the Barley (Hordeum vulgare L.) Aquaporin Gene Family
Source: PLoS One. 2015 Jun 9;10(6):e0128025. doi: 10.1371/journal.pone.0128025 (PMC4461243; doi:10.1371/journal.pone.0128025)
Supplement: S5 Fig — (DOCX) [file pone.0128025.s005.docx]

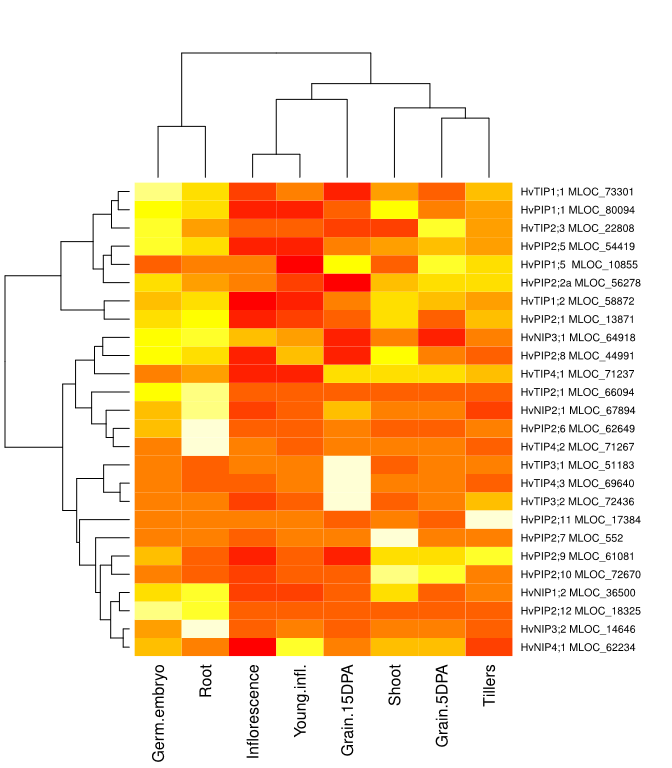


**S5 Figure. Heat map of expression levels of aquaporins from the IBGSC barley genome project RNA-seq data for 8 tissues.**

Germ.embryo: 4-day embryos dissected from germinating grains; Root: roots from the seedlings (10 cm shoot stage); Inflorescence: developing inflorescences (1-1.5 cm); Young.infl: Young developing inflorescences (5mm); Grain.15DPA: developing grain, bracts removed (15 DPA); Shoot: shoots from the seedlings (10 cm shoot stage); Grain.5DPA: developing grain, bracts removed (5 DPA); Tillers: developing tillers at six-leaf stage, third internode.
