# Supplementary material for: Identification and Expression Analysis of the Barley (Hordeum vulgare L.) Aquaporin Gene Family
Source: PLoS One. 2015 Jun 9;10(6):e0128025. doi: 10.1371/journal.pone.0128025 (PMC4461243; doi:10.1371/journal.pone.0128025)
Supplement: S1 Table — (DOCX) [file pone.0128025.s006.docx]

**S1 Table Barley aquaporins identified from leaf mRNA-seq data**

| **No.** | **Aquaporin UniGene/**  **NTC number** | **mRNA-Seq reads matching UniGene/NTC** | | **Fold change**  **under salinity** | **P-value** | **Base-mean expression** | **Corresponding NCBI cDNA sequences** | **Barley aquaporin**  **gene annotations** | |
| --- | --- | --- | --- | --- | --- | --- | --- | --- | --- |
|  |  |  |  |  |  |  | **GenBank accession number^Reference(s)^** | **Annotation** | **Reference for annotation** |
|  |  | **Control** | **Salt** |  |  |  |  |  |  |
| **PIPs** | | | | | | | | | |
| 1. | Hv.23281 | 6,858 | 13,491 | **+1.94** | 0.23 | 10,158.46 | X76911^4^; AK249573^15^; AB286964^19ds^ | HvPIP1;1 | 18^ds^ |
| 2. | Hv.20382 | 778 | 1,533 | **+1.97** | 0.01* | 1,155.5 | AB275278^19ds^ | HvPIP1;2 | 18^ds^ |
| 3. | Hv.12850 | 3,087 | 9,213 | **+2.93** | 0.05 | 6,306.80 | AB009308^7^; AK251251^15^ | HvPIP1;3 | 7 |
| 4. | Hv.23273 | 7,981 | 15,455 | **+1.93** | 0.24 | 12,009.47 | AB275279^19ds^ | HvPIP1;4 | 18^ds^ |
| 5. | Hv.11248 | 812 | 1,043 | +1.27 | 0.65 | 930.03 | AB009309^7^; AK360427^11^; AK359326^11^ | HvPIP1;5 | 7 |
| 6. | Hv.58 | 3,819 | 7,722 | **+1.99** | 0.21 | 5,762.01 | AB009307^7^; AB219366^12^; AK250654^15^ | HvPIP2;1 | 7 |
| 7. | Hv.18271 | 1,309 | 1,030 | -1.19 | 0.66 | 1,207.63 | AB377269^5ds^; AK250563^15^; AK253017^15^ | HvPIP2;2 | 5^ds^ |
| 8. | Hv.24509 | 1,847 | 1,312 | -1.17 | 0.65 | 1,920.39 | AK376080^11^; AK353861^11^; AK249631^15^; AB275280^19ds^ | HvPIP2;3 | 18^ds^ |
| 9. | Hv.23054 | 4,913 | 6,339 | +1.27 | 0.66 | 5,982.17 | AB219525^12ds^; AK252600^15^ | HvPIP2;4 | 12^ds^ |
| 10. | Hv.23400 | 693 | 1,607 | **+2.33** | 0.10 | 1,209.83 | AB377270^5ds^; AK370703^11^; AK370379^11^; GU989200^13ds^ | HvPIP2;5 | 5^ds^ |
| 11. | Hv.26580 | 5,527 | 2,772 | **-1.96** | 0.19 | 4,166.92 | GU584120^2^; AK359099^11^; AK359187^11^; AK248491^15^ | HvPIP2;7 | 2 |
| 12. | Hv.22567 | 147 | 340 | **+2.07** | 0.19 | 243.5 | AK359199^11^; AK356299^11^; ^18^AB808658 | HvPIP2;8 | 18 |
| 13. | Hv.25954 | 407 | 247 | **-1.68** | 0.32 | 328.69 | AK373720^11^ | HvPIP2;10 | This study |
| 14. | Hv.33209 | 53 | 72 | +1.22 | - | 62.5 | - | HvPIP2;6 | This study |
| 15. | Hv.15627 | 55 | 27 | -1.07 | 0.42 | 45.60 | AK361545^11^; AK361542^11^ | HvPIP2;9 | This study |
| **TIPs** | | | | | | | | | |
| 16. | Hv.10067 | 1,676 | 2,746 | **+1.62** | 0.37 | 2,210.97 | AB540221^9ds^; AK367756^11^; AK359670^11^; X80266^17^ | HvTIP1;1 | 9^ds^ |
| 17. | Hv.23472 | 10,498 | 10,689 | +1.02 | 1.00 | 10,613.05 | GU584119^1^; AB540226^6ds^; AK372282^11^; AK355942^11^; AK367251^11^; AK253104^15^ | HvTIP1;2 | 6^ds^ |
| 18. | Hv.24633 | 26 | 33 | +1.14 | - | 29.5 | AB540222^9ds^; AK251090^15^; AK250814^15^ | HvTIP2;1 | 9^ds^ |
| 19. | Hv.640 | 21 | 33 | +1.41 | - | 27.0 | AB540223^6ds^; AK363660^11^ | HvTIP2;2 | 6^ds^ |
| 20. | Hv.22828 | 230 | 604 | **+2.60** | 0.06 | 416.41 | GU584121^1ds^; AB261102^8ds^; AB540224^9ds^; EU872296^14ds^; AK248215^15^; AK249965^15^ | HvTIP2;3 | 9^ds^ |
| 21. | Hv.7640 | 0 | 2 | ND | 0.71 | 0.99 | AB540228^6ds^; AK376769^11^ | HvTIP3;1 | 6^ds^ |
| 22. | Hv.33058 | 9 | 22 | **+2.40** | 0.30 | 15.45 | AK373620^11^ | HvTIP3;2 | This study |
| 23. | Hv.3298 | 16,297 | 1,040 | **-15.89** | 5.77e^-6^* | 8,734.52 | AB540225^9ds^; AK364960^11^; AK368258^11^; AK358374^11^ | HvTIP4;1 | 9^ds^ |
| **NIPs** | | | | | | | | | |
| 24. | Hv.1160 | 9 | 9 | +1.29 | 1.00 | 9.00 | AB540230^6ds^; AK356027^11^ | HvNIP1;1 | 6^ds^ |
| 25. | Hv.20353 | 183 | 271 | **+1.58** | 0.48 | 231.62 | AB540231^6ds^; AK365010^11^ | HvNIP1;2 | 6^ds^ |
| 26. | Hv.30571 | 147 | 79 | **-1.76** | 0.26 | 113.29 | AB447482^3^; AK363953^11^; GQ496520^16^; GQ496519^16^ | HvNIP2;1 | 3; 16 |
| 27. | Hv.808 | 510 | 660 | +1.27 | 0.64 | 600.36 | AB540229^9ds^; AB447484^10ds^ | HvNIP2;2 | 10^ds^ |
| 28. | Hv.32277 | 83 | 110 | **+1.51** | 0.55 | 108.34 | AK360552^11^; AK357908^11^ | HvNIP2;3 | This study |
| 29. | Hv.12321 | 49 | 36 | -1.33 | 0.65 | 43.05 | - | HvNIP3;1 | This study |
|  | NTC10314 | 8 | 7 | -1.39 | 0.90 | 7.50 |  |  |  |
|  | NTC43512 | 16 | 6 | **-2.23** | 0.25 | 11.04 |  |  |  |
| **SIPs** | | | | | | | | | |
| 30. | Hv.9995 | 52 | 58 | +1.08 | 0.911 | 54.98 | AK355004^11^; AK252830^15^ | HvSIP1;1 | 2; This study |
| 31. | Hv.13190 | 218 | 369 | **+1.66** | 0.32 | 292.87 | AK364835^11^; AK364572^11^ | HvSIP2;1 | 2; This study |

The AQPs annotated in this study are highlighted in grey. Values considered indicative of differential gene expression (fold change ≥ +1.5 or ≤ -1.5) are shown in **bold,** while the statistically significant (p < 0.05) differentially expression is shown with an asterisk (*). ND: Fold change could not be determined due to the low number of reads matching the UniGene; ‘-’ indicates no information available.

**References for annotations:** ^ds^Direct submission; ^1^Besse *et al*., 2010 ds; ^2^Besse *et al*., 2011 J Exp Bot 62: 4127-4142; ^3^Chiba *et al*., 2008 ds; ^4^Hollenbach and Deitz, 1995 Botanica Acta 108: 425-431; ^5^Horie and Katsuhara, 2008 ds; ^6^Horie *et al*., 2010 ds; ^7^Katsuhara *et al*., 2002 Plant Cell Physiol 43: 885-893; ^8^Ligaba *et al*., 2006 ds; ^9^Ligaba *et al*., 2010 ds; ^10^Ma, 2008 ds; ^11^Matsumoto *et al*., 2011 Plant Physiol 156: 20-28; ^12^Panda *et al*., 2005 ds; ^13^Panda *et al*., 2010 ds; ^14^Pedas and Husted, 2008 ds; ^15^Sato *et al*., 2009 DNA Res 16: 81-89; ^16^Schnurbusch *et al*., 2010 Plant Physiol 153: 1706-1715; ^17^Schunmann and Ougham, 1996 Plant Mol Biol 31: 529-537; ^18^Shibasaka et al., 2012 Plant Signal Behav 7: 1648-1652; ^19^Sugimoto *et al*., 2006 ds.
