# Supplementary material for: Identification and Expression Analysis of the Barley (Hordeum vulgare L.) Aquaporin Gene Family
Source: PLoS One. 2015 Jun 9;10(6):e0128025. doi: 10.1371/journal.pone.0128025 (PMC4461243; doi:10.1371/journal.pone.0128025)
Supplement: S2 Table — (DOCX) [file pone.0128025.s007.docx]

| >MLOC_23039.1 | MAKDIEAAPPGGEYAAKDYSDPPPAPLFDAEELTKWSLYRAVIAEFVATLLFLYITVATVIGYKHQADPAGPNAADAACSGVGILGIAWAFGG |
| --- | --- |
| >MLOC_24245.1 | AVTFGLLLARKVSLPRAFFYVAAQFLGAICGAAMVRVVHGAHHYELYGGDANEVAPLQGGGIGGRGRRDVLSRLHRVLGDRPEAHGAGLPRA |
| >MLOC_37440.1 | MVHLATIPITGTGI**NPA**RSFGAAVIYNNEKAWDDHWIFWVGPFIGAAIAVAYHQYVLRASATKLGSSASFGRS |
| >MLOC_39721.2 | MFVISGVATDNRAIGELAGLAVGATVLLNVLFAGYSTITPTFTLFYSPFWTLVVNKAIF |
| >MLOC_43388.1 | sMPEQYKHMLGGPSLKVDPHTGAAAEGVLTFVITFAVLCIIVKGPRNPIVKTAMLSVSTVSLVLTGAAYTGPSM**NPA**NVHYAHSLTVFLYFHLIAKLIHGTAAPDTELSSIRMDKPLYCLFGFDIDGQLTDLVMQMTPFWHVRLELGNQILYDP |
| >MLOC_45579.2 | APGSRSRASSTRSSPAGSPSPTWPSPPPRPSSPSSSSPSTSSSSSPSWPPTPAWYVGELAGIAVGAAVTLNIVVAGPTTGGSMNPVRTLGQAVAAGNYRQLWIYV |
| >MLOC_58871.1 | MDDQKRFRLRSSVLGGGELLVRALADEADVVDEGGDGGGRQRRSRGAAPPS |
| >MLOC_67538.1 | MFTWNSTEIAVSSDSSRGFLVLIPLNCYVQAFGWAYVNNQHNTWEQLYVYWICPFIGAILAAWTFRAVFPPPAPKPKTKKA |
| >MLOC_72435.1 | QRARVLVHLPEAVDGAFGGEDEHGGGDELRQHGAGSRRQQGLRPRGPAGGETSWQHGPRCWLVKDLTSFPFSPLSLRGDPLVRDELHSSSTS |

**S2 Table AQP-like sequences identified from the low confidence gene set of the IBGSC barley genome project**
