# Supplementary material for: Identification and Expression Analysis of the Barley (Hordeum vulgare L.) Aquaporin Gene Family
Source: PLoS One. 2015 Jun 9;10(6):e0128025. doi: 10.1371/journal.pone.0128025 (PMC4461243; doi:10.1371/journal.pone.0128025)
Supplement: S3 Table — (DOCX) [file pone.0128025.s008.docx]

**S3 Table Pairwise identities between barley and rice aquaporins**

|  | | **OsPIPs** | | | | | | | | | | |  |  | **OsTIPs** | | | | | | | | | |
| --- | --- | --- | --- | --- | --- | --- | --- | --- | --- | --- | --- | --- | --- | --- | --- | --- | --- | --- | --- | --- | --- | --- | --- | --- |
|  |  | **1;1** | **1;2** | **1;3** | **2;1** | **2;2** | **2;3** | **2;4** | **2;5** | **2;6** | **2;7** | **2;8** |  |  | **1;1** | **1;2** | **2;1** | **2;2** | **3;1** | **3;2** | **4;1** | **4;2** | **4;3** | **5;1** |
| **HvPIPs**  **HvPIPs** | **1;1** | 92.3 | 91.6 | 86.5 | 62.8 | 62.6 | 63.2 | 61.7 | 63.3 | 47.4 | 56.7 | 52.6 | **HvTIPs** | **1;1** | 89.6 | 70.6 | 59.3 | 57.7 | 47.1 | 47.2 | 45.8 | 42.7 | 49.0 | 33.8 |
|  |  | 85.6 | 89.6 | 84.5 | 68.1 | 67.6 | 68.3 | 66.3 | 66.6 | 58.4 | 64.5 | 59.7 |  |  | 89.3 | 78.1 | 68.1 | 73.0 | 63.4 | 61.5 | 57.9 | 59.2 | 63.4 | 52.5 |
|  | **1;2** | 87.6 | 87.3 | 89.3 | 61.1 | 61.6 | 62.9 | 59.0 | 60.3 | 47.2 | 56.7 | 52.9 |  | **1;2** | 71.4 | 90.8 | 55.9 | 53.9 | 49.4 | 45.3 | 41.5 | 40.3 | 46.6 | 36.3 |
|  |  | 81.2 | 83.2 | 85.7 | 68.0 | 67.1 | 67.5 | 65.9 | 66.2 | 56.3 | 62.5 | 59.5 |  |  | 77.9 | 87.3 | 66.7 | 70.7 | 63.6 | 59.8 | 54.8 | 58.4 | 63.4 | 52.0 |
|  | **1;3** | 87.6 | 86.9 | 88.0 | 62.1 | 61.9 | 63.2 | 59.4 | 61.3 | 47.7 | 57.0 | 53.5 |  | **2;1** | 58.3 | 54.3 | 90.7 | 68.0 | 44.1 | 41.4 | 43.6 | 43.0 | 48.2 | 39.2 |
|  |  | 80.5 | 82.7 | 82.8 | 67.6 | 66.3 | 67.2 | 66.1 | 66.2 | 55.5 | 62.9 | 57.9 |  |  | 68.9 | 65.6 | 89.3 | 77.0 | 58.6 | 54.4 | 54.1 | 58.7 | 62.6 | 57.0 |
|  | **1;4** | 88.0 | 87.3 | 88.6 | 62.1 | 62.2 | 63.6 | 59.7 | 61.7 | 47.4 | 57.4 | 53.5 |  | **2;2** | 57.3 | 52.7 | 88.3 | 68.8 | 44.1 | 40.1 | 42.8 | 43.0 | 47.4 | 37.0 |
|  |  | 80.5 | 82.7 | 83.2 | 67.7 | 66.5 | 67.2 | 66.2 | 66.3 | 55.1 | 62.8 | 57.9 |  |  | 54.0 | 51.7 | 68.6 | 60.2 | 49.8 | 45.7 | 44.0 | 48.5 | 49.4 | 47.4 |
|  | **1;5** | 94.1 | 89.3 | 84.5 | 63.8 | 61.3 | 61.9 | 61.3 | 62.7 | 48.3 | 54.4 | 52.7 |  | **2;3** | 59.7 | 53.5 | 67.0 | 90.7 | 41.5 | 37.6 | 41.2 | 36.2 | 43.7 | 35.4 |
|  |  | 92.3 | 87.5 | 80.5 | 66.1 | 65.3 | 65.3 | 63.9 | 63.7 | 55.1 | 60.5 | 57.3 |  |  | 72.6 | 66.7 | 75.3 | 91.2 | 58.0 | 53.3 | 54.8 | 55.3 | 61.1 | 53.0 |
|  | **2;1** | 61.7 | 62.5 | 61.3 | 76.9 | 88.2 | 86.8 | 79.5 | 79.5 | 52.5 | 61.8 | 60.0 |  | **3;1** | 50.5 | 50.5 | 46.0 | 43.4 | 88.3 | 57.4 | 35.8 | 38.4 | 42.5 | 32.9 |
|  |  | 63.9 | 66.9 | 65.4 | 79.2 | 87.5 | 85.3 | 78.2 | 77.6 | 61.1 | 67.0 | 63.4 |  |  | 63.8 | 61.7 | 57.0 | 60.2 | 88.0 | 66.9 | 50.9 | 55.0 | 56.4 | 51.3 |
|  | **2;2** | 63.7 | 62.1 | 59.8 | 72.2 | 68.3 | 70.2 | 67.2 | 67.9 | 64.8 | 66.6 | 59.7 |  | **3;2** | 48.6 | 46.3 | 41.4 | 41.4 | 56.5 | 81.3 | 37.8 | 34.4 | 41.6 | 34.5 |
|  |  | 64.1 | 67.4 | 66.4 | 78.0 | 71.3 | 74.7 | 75.0 | 75.1 | 73.4 | 72.0 | 64.7 |  |  | 61.4 | 61.0 | 57.8 | 58.6 | 69.5 | 84.1 | 51.6 | 54.5 | 57.0 | 53.4 |
|  | **2;2a** | 52.5 | 52.1 | 50.3 | 56.0 | 56.5 | 57.4 | 56.1 | 56.4 | 75.5 | 54.6 | 50.0 |  | **4;1** | 42.2 | 39.9 | 42.2 | 41.0 | 34.4 | 34.5 | 80.9 | 55.3 | 55.5 | 27.6 |
|  |  | 57.1 | 60.0 | 59.5 | 67.6 | 62.9 | 65.3 | 65.1 | 65.3 | 78.3 | 67.0 | 58.1 |  |  | 54.2 | 55.3 | 52.6 | 53.4 | 48.9 | 49.1 | 81.1 | 63.5 | 64.7 | 44.5 |
|  | **2;3** | 62.0 | 62.7 | 62.0 | 80.1 | 81.7 | 89.3 | 79.3 | 79.3 | 54.8 | 62.8 | 59.9 |  | **4;2** | 45.8 | 43.3 | 45.6 | 39.9 | 38.4 | 37.8 | 60.5 | 70.2 | 60.8 | 31.8 |
|  |  | 63.2 | 66.6 | 65.7 | 81.0 | 84.8 | 90.2 | 80.0 | 78.2 | 63.2 | 67.9 | 64.1 |  |  | 63.7 | 61.9 | 63.2 | 63.6 | 57.4 | 53.2 | 68.6 | 75.0 | 71.1 | 52.6 |
|  | **2;4** | 61.7 | 62.4 | 61.7 | 80.1 | 81.0 | 88.3 | 78.9 | 78.9 | 54.3 | 62.8 | 59.9 |  | **4;3** | 35.7 | 33.8 | 33.8 | 31.3 | 30.1 | 29.7 | 39.9 | 39.8 | 52.8 | 24.6 |
|  |  | 63.2 | 66.5 | 65.6 | 81.6 | 84.5 | 90.1 | 80.6 | 79.4 | 62.6 | 67.6 | 64.4 |  |  | 48.6 | 46.5 | 48.1 | 47.1 | 46.5 | 44.8 | 52.7 | 56.9 | 63.7 | 41.5 |
|  | **2;5** | 60.9 | 61.5 | 59.5 | 90.0 | 77.3 | 81.0 | 78.3 | 77.6 | 52.8 | 63.1 | 61.4 |  | **5;1** | 35.4 | 35.8 | 40.3 | 38.3 | 32.2 | 32.2 | 28.7 | 32.3 | 36.8 | 79.2 |
|  |  | 64.2 | 68.0 | 66.3 | 90.1 | 80.5 | 82.1 | 83.6 | 83.2 | 63.9 | 69.7 | 65.3 |  |  | 54.1 | 52.1 | 58.3 | 57.5 | 53.2 | 51.5 | 47.2 | 52.6 | 53.7 | 86.1 |
|  | **2;6** | 33.8 | 33.8 | 33.2 | 44.8 | 43.1 | 41.9 | 51.0 | 48.6 | 30.7 | 32.2 | 35.8 |  |  |  |  |  | |  |  | | | | |
|  |  | 35.1 | 37.8 | 37.8 | 47.5 | 45.8 | 47.8 | 51.6 | 50.6 | 34.2 | 39.1 | 38.0 |  |  |  |  |  |  |  |  |  |  |  |  |
|  | **2;7** | 55.8 | 55.5 | 55.8 | 63.9 | 62.0 | 66.1 | 61.2 | 62.9 | 49.4 | 77.4 | 54.1 |  |  |  |  |  | |  |  | | | | |
|  |  | 60.6 | 62.8 | 64.7 | 72.8 | 70.1 | 72.4 | 70.6 | 70.9 | 63.2 | 75.0 | 61.9 |  |  |  |  | |  | |  |  |  |  |  |
|  | **2;7a** | 39.1 | 40.0 | 38.9 | 40.0 | 41.1 | 42.4 | 39.5 | 40.6 | 34.8 | 44.1 | 41.1 |  |  |  |  |  |  |  |  |  |  |  |  |
|  |  | 41.8 | 43.6 | 46.5 | 46.2 | 48.0 | 47.5 | 47.1 | 47.7 | 41.3 | 48.5 | 41.7 |  |  |  |  |  |  |  |  |  |  |  |  |
|  | **2;8** | 54.3 | 55.6 | 54.3 | 65.4 | 64.0 | 65.1 | 67.3 | 68.0 | 47.4 | 55.0 | 64.9 |  |  |  |  |  |  |  |  |  |  |  |  |
|  |  | 61.8 | 65.7 | 63.3 | 70.8 | 68.6 | 68.8 | 71.1 | 71.0 | 56.4 | 63.8 | 67.5 |  |  |  |  |  |  |  |  |  |  |  |  |
|  | **2;9** | 53.4 | 54.7 | 53.9 | 61.0 | 59.4 | 60.7 | 61.3 | 63.0 | 44.4 | 55.3 | 62.2 |  |  |  |  |  |  |  |  |  |  |  |  |
|  |  | 59.8 | 62.5 | 61.9 | 68.2 | 66.1 | 67.8 | 68.0 | 68.3 | 56.5 | 62.7 | 67.7 |  |  |  | |  |  |  |  |  |  |  |  |
|  | **2;10** | 49.0 | 49.3 | 50.3 | 52.9 | 51.7 | 55.0 | 54.5 | 55.1 | 45.0 | 63.8 | 48.9 |  |  |  |  |  |  |  |  |  |  |  |  |
|  |  | 59.0 | 62.4 | 61.1 | 65.8 | 64.9 | 66.6 | 66.1 | 65.2 | 58.2 | 76.9 | 56.7 |  |  |  |  |  |  |  |  |  |  |  |  |
|  | **2;11** | 55.4 | 57.2 | 55.7 | 65.1 | 64.7 | 66.4 | 65.3 | 65.0 | 48.8 | 58.7 | 66.1 |  |  |  |  |  |  |  |  |  |  |  |  |
|  |  | 59.7 | 62.5 | 60.5 | 68.3 | 66.7 | 67.0 | 67.3 | 67.2 | 65.9 | 63.3 | 67.8 |  |  |  |  |  |  |  |  |  |  |  |  |
|  | **2;12** | 57.4 | 59.2 | 56.6 | 70.0 | 67.1 | 68.4 | 69.8 | 69.7 | 51.1 | 59.7 | 69.5 |  |  |  |  |  |  |  |  |  |  |  |  |
|  |  | 61.8 | 65.8 | 61.9 | 74.2 | 71.2 | 73.6 | 73.9 | 73.6 | 70.9 | 67.0 | 71.3 |  |  |  | | |  |  |  |  |  |  |  |

|  | | **OsNIPs** | | | | | | | | | | | |
| --- | --- | --- | --- | --- | --- | --- | --- | --- | --- | --- | --- | --- | --- |
|  |  | **1;1** | **1;2** | **1;3** | **1;4** | **2;1** | **2;2** | **3;1** | **3;2** | **3;3** | **3;4** | **3;5** | **4;1** |
| **HvNIPs** | **1;1** | 84.9 | 51.1 | 60.2 | 50.1 | 35.9 | 35.7 | 38.1 | 28.6 | 28.8 | 23.9 | 26.2 | 24.1 |
|  |  | 83.1 | 49.7 | 66.0 | 60.1 | 49.8 | 52.0 | 47.8 | 35.0 | 38 | 38 | 34.5 | 46.0 |
|  | **1;2** | 56.4 | 48.9 | 67.8 | 41.0 | 30.1 | 30.1 | 32.0 | 30.1 | 29.5 | 24.7 | 29.4 | 22.0 |
|  |  | 58.3 | 55.6 | 69.9 | 51.4 | 41.9 | 43.7 | 47.2 | 40.0 | 37.9 | 39.4 | 41.8 | 38.3 |
|  | **2;1** | 35.3 | 31.4 | 34.8 | 33.4 | 78.9 | 73.2 | 33.7 | 24.7 | 24.8 | 20.7 | 22.4 | 23.3 |
|  |  | 51.1 | 38.3 | 47.1 | 45.7 | 84.0 | 79.0 | 43.8 | 34.3 | 35.7 | 36.7 | 34.5 | 47.0 |
|  | **2;2** | 36.5 | 32.0 | 35.8 | 33.5 | 72.3 | 87.0 | 34.4 | 23.9 | 24.3 | 19.7 | 22.1 | 24.9 |
|  |  | 52.9 | 38.6 | 49.6 | 46.8 | 78.5 | 88.5 | 45.4 | 34.3 | 36.1 | 35.9 | 34.0 | 48.2 |
|  | **2;2a** | 37.2 | 32.8 | 36.0 | 33.7 | 73.8 | 87.7 | 34.6 | 23.6 | 25.5 | 21.4 | 21.9 | 27.6 |
|  |  | 51.4 | 37.8 | 49.3 | 47.5 | 77.0 | 87.6 | 43.6 | 34.5 | 35.8 | 35.8 | 33.4 | 47.6 |
|  | **3;1** | 37.8 | 36.3 | 39.3 | 32.2 | 33.8 | 33.5 | 88.5 | 35.8 | 37.8 | 29.9 | 30.0 | 26.7 |
|  |  | 49.3 | 37.8 | 47.8 | 47.7 | 44.7 | 46.4 | 71.6 | 37.3 | 41.1 | 41.3 | 37.3 | 44.7 |
|  | **3;2** | 31.0 | 30.4 | 35.9 | 34.2 | 27.2 | 26.5 | 46.4 | 53.1 | 58.9 | 38.5 | 42.8 | 25.1 |
|  |  | 41.4 | 32.8 | 42.0 | 45.0 | 37.5 | 36.6 | 40.5 | 49.3 | 55.7 | 44.7 | 45.5 | 37.4 |
|  | **4;1** | 27.0 | 24.4 | 23.8 | 24.5 | 25.0 | 25.6 | 26.2 | 21.1 | 20.6 | 19.3 | 18.7 | 59.8 |
|  |  | 47.9 | 36.1 | 44.1 | 42.5 | 46.7 | 49.1 | 43.4 | 32.1 | 33.8 | 35.7 | 33.2 | 72.2 |

|  | | **OsSIPs** | |  |
| --- | --- | --- | --- | --- |
|  |  | **1;1** | **2;1** |  |
| **HvSIPs** | **1;1** | 85.0 | 26.7 |  |
|  |  | 83.0 | 46.3 |  |
|  | **2;1** | 26.6 | 74.5 |  |
|  |  | 46.2 | 83.7 |  |

% IDs of putative protein sequences highlighted in grey; % IDs of cDNA sequences shown in clear boxes. Alignments of the cDNA or putative protein sequences were analysed by pairwise identities in BioEdit.
