# Supplementary material for: Identification and Expression Analysis of the Barley (Hordeum vulgare L.) Aquaporin Gene Family
Source: PLoS One. 2015 Jun 9;10(6):e0128025. doi: 10.1371/journal.pone.0128025 (PMC4461243; doi:10.1371/journal.pone.0128025)
Supplement: S4 Table — (DOCX) [file pone.0128025.s009.docx]

**S4 Table Tissue types of ESTs representing barley aquaporins**

| **No.** | **Barley aquaporin gene** | **Tissue types** | | | | | | | | | | **Total** |
| --- | --- | --- | --- | --- | --- | --- | --- | --- | --- | --- | --- | --- |
|  |  | **Apex** | **Epidermis** | **Flower** | **Leaf** | **Maternal** | **Root** | **Seed** | **Shoot** | **Spike** | **Not specified** |  |
|  | **PIPs** |  |  |  |  |  |  |  |  |  |  |  |
| 1. | HvPIP1;1^ |  | 25 | 17 | 83 | 3 | 146 | 51 | 289 | 6 | 1 | **621** |
| 2. | HvPIP1;2^ |  |  |  |  |  | 27 |  |  | 2 |  | **29** |
| 3. | HvPIP1;3^ |  | 23 | 3 | 74 |  | 100 | 37 | 46 | 6 |  | **289** |
| 4. | HvPIP1;4^ |  | 4 |  | 11 | 1 | 24 | 3 | 11 | 1 |  | **55** |
| 5. | HvPIP1;5^ | 2 |  | 1 | 2 |  | 8 | 22 | 8 | 6 |  | **49** |
| 6. | HvPIP2;1^ |  | 2 |  | 51 |  | 48 | 8 | 17 |  |  | **126** |
| 7. | HvPIP2;2^ |  | 3 | 5 | 7 | 4 | 42 | 27 | 293 | 11 |  | **392** |
| 8. | HvPIP2;3^ |  | 5 | 5 | 16 |  | 60 | 9 | 20 | 1 |  | **116** |
| 9. | HvPIP2;4^ |  | 16 | 1 | 23 | 1 | 37 | 8 | 17 | 2 |  | **105** |
| 10. | HvPIP2;5^ | 1 | 14 | 7 | 12 |  | 49 | 50 | 87 | 7 |  | **227** |
| 11. | HvPIP2;6^ |  |  |  |  |  | 5 |  | 1 |  |  | **6** |
| 12. | HvPIP2;7^ |  |  |  | 4 |  |  |  | 2 |  |  | **6** |
| 13. | HvPIP2;8^ |  |  |  |  |  |  |  | 4 | 1 |  | **5** |
| 14. | HvPIP2;9^ |  |  | 1 | 2 | 1 |  |  |  |  |  | **4** |
| 15. | HvPIP2;10^ |  |  |  |  |  |  |  | 2 |  |  | **2** |
|  | **TOTAL** | **3** | **92** | **40** | **285** | **10** | **546** | **215** | **797** | **43** | **1** | **2,032** |
|  | **TIPs** |  |  |  |  |  |  |  |  |  |  |  |
| 16. | HvTIP1;1^ | 8 | 7 |  | 24 | 4 | 150 | 219 | 205 | 6 |  | **653** |
| 17. | HvTIP1;2^ |  | 5 | 3 | 24 | 5 | 47 | 17 | 6 | 1 |  | **108** |
| 18. | HvTIP2;1^ |  |  |  |  |  | 32 |  |  |  |  | **32** |
| 19. | HvTIP2;2^ |  |  |  |  |  | 12 |  | 1 |  |  | **13** |
| 20. | HvTIP2;3^ | 8 | 1 | 19 | 50 | 8 | 58 | 120 | 66 | 10 | 1 | **341** |
| 21. | HvTIP3;1^ |  |  |  | 1 |  |  | 52 |  | 13 |  | **66** |
| 22. | HvTIP3;2^ |  |  |  |  |  |  | 1 |  |  |  | **1** |
| 23. | HvTIP4;1^ |  | 12 |  | 13 | 1 | 6 | 3 | 3 | 1 |  | **39** |
| 24. | HvTIP4;3 |  |  |  |  |  |  | 5 |  |  |  | **5** |
|  | **TOTAL** | **16** | **25** | **52** | **112** | **18** | **305** | **417** | **281** | **31** | **1** | **1,258** |
|  | **NIPs** |  |  |  |  |  |  |  |  |  |  |  |
| 25. | HvNIP1;1^ | 1 | 1 | 1 | 1 |  | 1 | 3 | 1 |  |  | **9** |
| 26. | HvNIP1;2^ |  | 2 |  |  |  | 1 |  | 1 |  |  | **4** |
| 27. | HvNIP2;1^ |  |  |  |  |  | 3 |  |  |  |  | **3** |
| 28. | HvNIP2;2^ |  | 5 |  | 15 |  | 5 | 1 | 5 |  |  | **31** |
| 29. | HvNIP2;3^ |  |  |  | 12 |  |  |  |  |  |  | **12** |
| 30. | HvNIP3;1^ |  |  |  |  |  |  | 2 |  | 1 |  | **3** |
| 31 | HvNIP4;1 |  |  |  |  |  |  |  |  | 3 |  | **3** |
|  | **TOTAL** | **1** | **7** | **1** | **18** |  | **10** | **6** | **4** | **4** |  | **51** |
|  | **SIPs** |  |  |  |  |  |  |  |  |  |  |  |
| 32. | HvSIP1;1^ |  | 1 | 5 | 2 | 2 | 2 | 15 | 2 | 2 |  | **31** |
| 33. | HvSIP2;1^ |  | 1 |  | 2 |  | 1 | 1 |  | 1 |  | **6** |
|  | **TOTAL** |  | **2** | **5** | **4** | **2** | **3** | **16** | **2** | **3** |  | **37** |

The numbers represent the number of ESTs identified from NCBI in a particular tissue type. ESTs expressed in the leaf are shown in dark grey boxes, those expressed in other tissues are shown in clear boxes, and tissue types where no ESTs were found for a particular AQP are shown in grey boxes. AQPs with no corresponding ESTs are not included in table. ^AQPs identified from mRNA-seq data.
