# Supplementary material for: Identification and Expression Analysis of the Barley (Hordeum vulgare L.) Aquaporin Gene Family
Source: PLoS One. 2015 Jun 9;10(6):e0128025. doi: 10.1371/journal.pone.0128025 (PMC4461243; doi:10.1371/journal.pone.0128025)
Supplement: S5 Table — (DOCX) [file pone.0128025.s010.docx]

**S5Table Treatment conditions of ESTs representing the barley aquaporins**

| **Barley aquaporin gene** | **Treatment conditions** | | | | | | | | | | | | | **Total** |
| --- | --- | --- | --- | --- | --- | --- | --- | --- | --- | --- | --- | --- | --- | --- |
|  | **Abiotic** | | | | | | | | | | **Biotic** |  | **No treatment** |  |
|  | **Cold** | **Drought** | | **Etiolation** | | **Low nitrogen** | **Salt** | | | **Waterlogging** | **Fungal infection** |  |  |  |
| **PIPs** | | | | | | | | | | | | | | |
| HvPIP1;1^ | 8 | 19 | 261 | |  | | | 1 | 2 | | 21 | 309 | | **621** |
| HvPIP1;2^ |  | 1 | 5 | |  | | | 2 | 4 | | 2 | 15 | | **29** |
| HvPIP1;3^ | 4 | 12 | 37 | | 1 | | | 4 | 8 | | 25 | 198 | | **289** |
| HvPIP1;4^ |  | 5 | 6 | | 1 | | |  |  | | 3 | 40 | | **55** |
| HvPIP1;5^ | 2 | 2 | 5 | |  | | |  | 1 | | 1 | 38 | | **49** |
| HvPIP2;1^ |  | 5 | 25 | | 1 | | | 2 | 2 | | 4 | 87 | | **126** |
| HvPIP2;2^ | 2 | 6 | 266 | |  | | |  | 2 | | 2 | 114 | | **392** |
| HvPIP2;3^ | 1 | 7 | 24 | | 2 | | | 1 | 3 | | 4 | 74 | | **116** |
| HvPIP2;4^ | 2 | 5 | 16 | |  | | | 2 | 3 | | 15 | 62 | | **105** |
| HvPIP2;5^ | 3 | 3 | 71 | | 1 | | | 1 | 1 | | 18 | 129 | | **227** |
| HvPIP2;6^ |  |  | 1 | |  | | |  | 1 | |  | 4 | | **6** |
| HvPIP2;7^ |  |  | 2 | |  | | |  |  | | 1 | 3 | | **6** |
| HvPIP2;8^ |  |  | 1 | |  | | |  |  | |  | 4 | | **5** |
| HvPIP2;9^ |  |  |  | |  | | |  |  | | 2 | 2 | | **4** |
| HvPIP2;10^ |  |  | 2 | |  | | |  |  | |  |  | | **2** |
| **TOTAL** | **22** | **65** | **722** | | **6** | | | **13** | **27** | | **98** | **1,079** | | **2,032** |
| **TIPs** | | | | | | | | | | | | | | |
| HvTIP1;1^ | 1 | 13 | 142 | | 2 | | |  | 12 | | 6 | 477 | | **653** |
| HvTIP1;2^ | 1 | 7 | 7 | |  | | | 1 | 3 | | 9 | 80 | | **108** |
| HvTIP2;1^ |  | 3 | 5 | | 1 | | | 2 | 2 | |  | 19 | | **32** |
| HvTIP2;2^ | 1 | 3 | 3 | |  | | |  | 1 | |  | 5 | | **13** |
| HvTIP2;3^ | 5 | 5 | 44 | |  | | |  |  | | 8 | 279 | | **341** |
| HvTIP3;1^ |  |  |  | |  | | |  |  | | 1 | 65 | | **66** |
| HvTIP3;2^ |  |  |  | |  | | |  |  | |  | 1 | | **1** |
| HvTIP4;1^ |  | 3 | 4 | |  | | |  |  | | 18 | 14 | | **39** |
| HvTIP4;3 |  |  |  | |  | | |  |  | |  | 5 | | **5** |
| **TOTAL** | **8** | **34** | **205** | | **3** | | | **3** | **18** | | **41** | **947** | | **1,258** |
| **NIPs** | | | | | | | | | | | | | | |
| HvNIP1;1^ |  | 1 | 1 | |  | | |  |  | | 1 | 6 | | **9** |
| HvNIP1;2^ |  | 1 | 1 | |  | | |  |  | | 2 |  | | **4** |
| HvNIP2;1^ |  |  |  | |  | | |  | 1 | |  | 2 | | **3** |
| HvNIP2;2^ |  |  | 5 | |  | | |  |  | | 11 | 15 | | **31** |
| HvNIP2;3^ |  |  |  | |  | | |  |  | | 3 | 9 | | **12** |
| HvNIP3;1^ |  |  |  | |  | | |  |  | |  | 3 | | **3** |
| HvNIP4;1 |  |  |  | |  | | |  |  | |  | 3 | | **3** |
| **TOTAL** |  | **2** | **5** | |  | | |  | **1** | | **10** | **33** | | **51** |
| **SIPs** | | | | | | | | | | | | | | |
| HvSIP1;1^ |  |  |  | |  | | |  |  | |  | 31 | | **31** |
| HvSIP2;1^ |  | 1 |  | |  | | |  |  | | 2 | 3 | | **6** |
| Total |  | **1** |  | |  | | |  |  | | **2** | **37** | | **37** |

The numbers represent the number of ESTs identified under a particular treatment condition. Treatment conditions where no ESTs were expressed for a particular AQP are shown in grey blocks. AQPs with no corresponding ESTs are not included in table. ^AQPs identified from mRNA-seq data.
